# Supramolecular Engineering of Narrow Absorption Bands by Exciton Coupling in Pristine and Mixed Solid-State Dye Aggregates

**DOI:** 10.1021/acscentsci.4c02157

**Published:** 2025-03-14

**Authors:** Tim Schembri, Julius Albert, Hendrik Hebling, Vladimir Stepanenko, Olga Anhalt, Kazutaka Shoyama, Matthias Stolte, Frank Würthner

**Affiliations:** † Universität Würzburg, Institut für Organische Chemie, Am Hubland, Würzburg 97074, Germany; ‡ Universität Würzburg, Center for Nanosystems Chemistry (CNC), Theodor-Boveri-Weg, Würzburg 97074, Germany

## Abstract

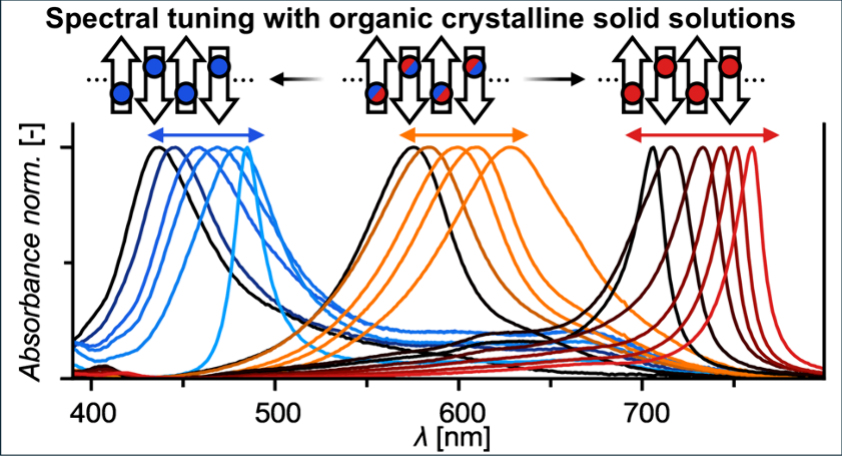

Tunability of functional properties in a continuous manner is desired but challenging to accomplish for organic solid-state materials. Herein, we describe a method for tuning optoelectronic properties of solid-state aggregates with narrow absorption bands. First, we systematically shift the absorption maxima of highly dipolar merocyanine dyes in solution by chemical alterations of their chromophore cores. This leaves their solid-state packing arrangements unchanged, affording similar J- and H-coupled aggregate absorption bands at different wavelengths. Next, mixing these isostructural dyes leads to a spectral fine-tuning of the mixed layers, which could be characterized as crystalline organic solid solutions and utilized in narrowband color-selective organic photodiodes. Finally, we devise a semiempirical model, which explains the observed spectral tuning in terms of the molecular exciton theory. Thus, we demonstrate narrowband absorbing solid-state aggregates spanning the wavelength range of 437–760 nm, whose absorption can be fine-tuned over 40% of the visible light range.

## Abstract 2

Spectral tuning of absorption properties for color sensing is achieved by design of isostructural compounds and binary mixtures thereof, which self-assemble into identical supramolecular aggregates.

## Introduction

Narrowband absorption, emission and spectral tunability are desired features for a plethora of applications of photonic and optoelectronic materials, including displays,^1^ high-resolution imaging^2^ and spectroscopy,^3^ as well as light communication.^4^ State-of-the-art organic photodiodes (OPDs) realize a highly selective spectral response (<100 nm) by either inducing a tunable narrowband photoresponse in otherwise broadband materials through sophisticated device engineering approaches such as μC resonance structures^5,6^ and charge collection narrowing,^7,8^ or by designing intrinsically narrowband absorbing photoactive materials. Particularly, stacked OPDs with high selectivity will enable multispectral or hyperspectral image sensing which is desirable for biomedical applications, environmental monitoring, and machine vision.^9^

To accomplish narrow absorption (or emission) bands, a single optical excitation, mostly S_0_ → S_1_, should prevail over a large spectral range, e. g. visible, and vibrational coupling to the electronic transition should be minimized. The dyes best fulfilling these criteria are polymethine dyes which include cyanine dyes,^10,11^ squaraine dyes,^12,13^ BODIPY dyes,^14,15^ merocyanine dyes close to the cyanine limit,^16,17^ some acceptor–donor–acceptor dyes,^18^ and recently developed multiresonance dyes.^19,20^ For the best case, i. e. a Franck–Condon transition only involving the vibrational lowest levels (*A*_00_ transition), the absorption band in solution can be as narrow as ≈400 cm^–1^ at room temperature.^21^ However, even for the best cases, a contamination of the color purity is mostly observed by a weak second *A*_01_ absorption band that is attributable to the excitation of a carbon–carbon stretching vibration in the first excited state.^22^

Furthermore, for most applications, dyes are not applied in dilute solutions but at higher concentrations in polymeric matrices or even as bulk materials, e. g. in thin films, where the absorption bands of dyes aggregates are strongly influenced by dye–dye interactions.^23−25^ As a consequence, isolated as well as narrow absorption bands which ensure highly selective photoresponses are only preserved in exceptional cases, e.g. for some J-aggregates,^26,27^ while for the majority of solid-state materials undesired band broadening or the splitting of the original band into multiple excitonic bands is observed.^28−30^ What makes the design of narrow bands for dye aggregates even more challenging is the fact that the specific arrangement of the dyes in a three-dimensional solid-state structure is rather unpredictable due to the nondirectionality of dispersion interactions, as exemplified by the coexistence of often multiple polymorphs for color pigments.^31^ Further, the understanding of excitonic coupling between neighboring dyes in aggregates reached only in recent years the level that allows for the prediction of spectral shifts and the involvement of vibrational transitions.^32−34^ Accordingly, rational approaches toward functional three-dimensional solid-state materials such as pigments (nanoparticles consisting of crystalline solid materials) and thin films with tailored optical properties^35,36^ remain far less explored compared to those for more simple one-dimensional dye aggregates.^37−42^

Taking inspiration from Desiraju’s supramolecular synthon approach in crystal engineering,^43,44^ which defines structural units within supramolecular assemblies or crystals that self-assemble by tailored intermolecular interactions, here we design solid-state materials with predictable packing structures driven by strong electrostatic interactions between dipolar merocyanine dyes. In particular, we realize precise spectral tunability^45^ through heteroatom doping for the utilized dyes and through physical mixing of structurally similar dyes. Given sufficiently similar crystal lattice energies, several combinations between two compounds were shown to form organic crystalline solid solutions (CSSs).^46,47^ CSSs describe statistically mixed cocrystals with a packing isostructural to that of the pristine compounds, enabling a variation of stoichiometry as well as physicochemical properties herein for the first time in a continuous manner.^48−50^

**Figure 1:**
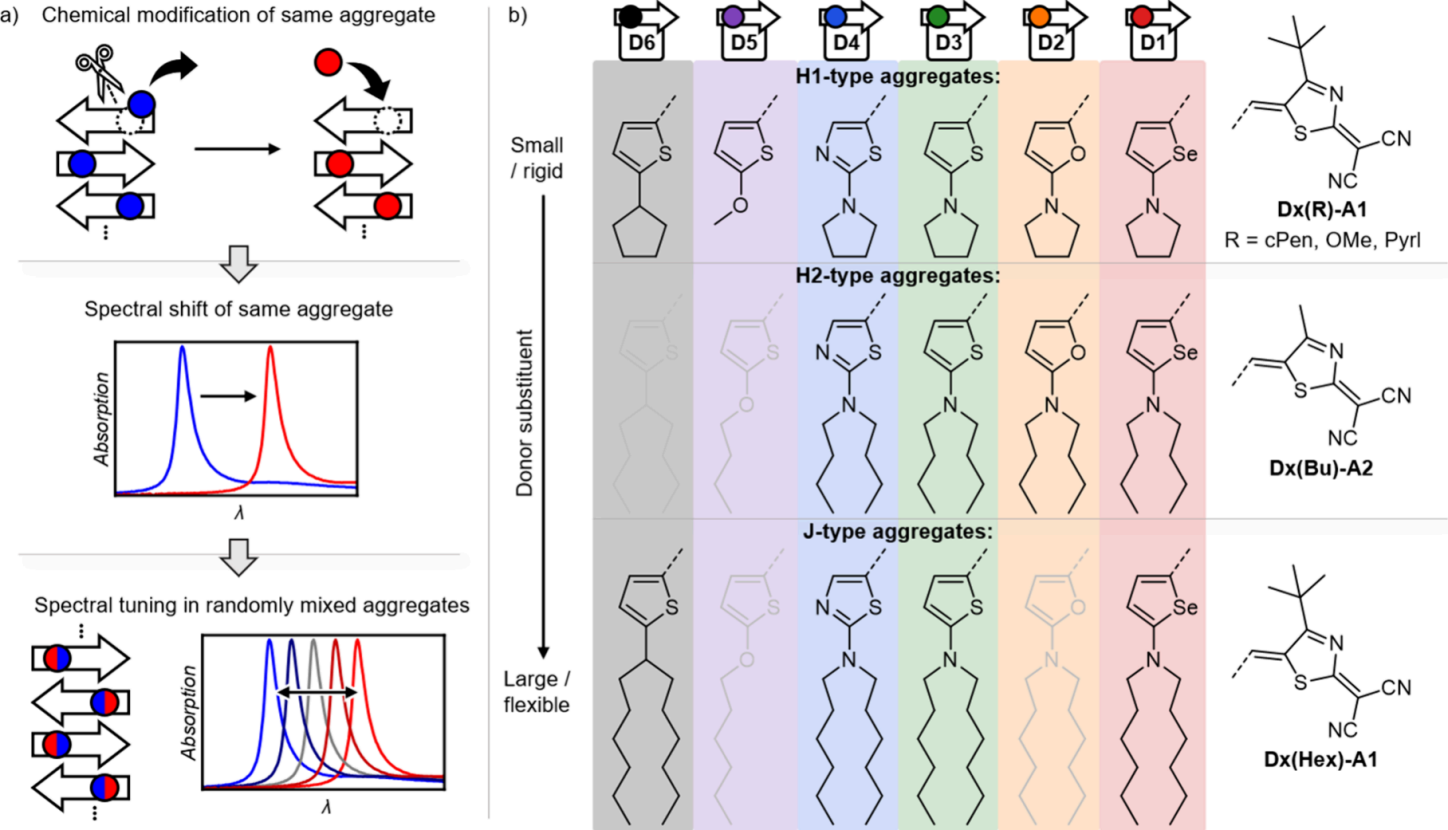
a) Schematic depiction of our concept for tuning of the position of an aggregate’s narrow absorption band by minor chemical modification (colored circles) at the dipolar chromophore core (arrows) resulting in a similar aggregate structure absorbing at different wavelengths as well as the additional possibility of spectral fine-tuning resulting from the statistical mixing of two isostructural chromophores. b) Chemical structures of investigated merocyanine dyes, which self-assemble into three distinct packing arrangements with respective exciton coupling (H1, H2, J); The dyes nomenclature (**Dx(R)-A1/A2**) represents the combination of the six donor heterocycles **D1**-**D6** with each respective substituent (**R**) in combination with one of the two acceptor units **A1** (*t*Bu) or **A2** (Me); the four greyed out structures correspond to molecules within this compound matrix which were not investigated due to findings from the complete H1-type series (*vide infra*).

The starting point for our research were merocyanine dyes **D3(Pyrl)-A1** and **D3(Hex)-A1** (Figure 1) which form crystalline solid-state semiconductor materials characterized by strong exchange narrowing due to intermolecular Coulomb coupling of the transition dipole moments (μ_eg_) of neighboring dyes.^23^ Upon self-assembly, driven by strong dipole–dipole interactions (ground-state dipole moment: μ_g_ ≈ 14 D), these dyes form either H- or J-coupled aggregates with ultranarrow absorption bands showing full-width-at-half-maximum (*fwhm*_opt_) values that could be applied for color-selective OPDs with narrow photoresponses at around 480 nm (21 nm, 900 cm^–1^) and 750 nm (11 nm, 200 cm^–1^).^51,52^ However, a spectral variation of such exchange-narrowed solid-state absorption bands could so far not be realized in our research despite of significant efforts during the last ten years dedicated to many other merocyanine dyes. Accordingly, we contemplated that the unique packing patterns observed for ultranarrow exciton-coupled dyes **D3(Pyrl)-A1** and **D3(Hex)-A1** might be transferred to other structurally similar dyes for the desired expansion of the wavelength range. As we will show in this article, this approach was highly successful and allowed us to gain insight into the exciton-coupling induced spectral changes in the solid state for homoaggregates of a larger variety of merocyanine dyes with rather different absorption characteristics in solution. Next, mixtures of different isostructural chromophores afforded so far unprecedented CSSs (= heteroaggregates), which enabled additional spectral tunability of their color-selective absorption bands and did not show any phase separation. This allowed for spectral fine-tuning covering about half of the visible wavelength range with narrow *fwhm*_opt_ values of down to 18 nm (350 cm^–1^) in the NIR at 749 nm, which could be exploited in OPD devices. A schematic overview of our concept along with the investigated compounds is displayed in Figure 1. The formation of CSSs is verified by X-ray crystal analyses and their spectral tuning is rationalized with the support of quantum chemical calculations, expanding the classical Kasha exciton coupling theory to mixed solid-state systems.

## Results and Discussion

### Materials Design

This study is based on three hypotheses for enabling the tuning of narrowband solid-state absorption of dipolar merocyanine dyes over a large wavelength range in a continuous manner (Figure 1a): I) Shape-complementary dyes, even if absorbing at rather different wavelengths as monomers in solution (λ_00_), will afford the same supramolecular packing structures in the solid state as long as their intermolecular interactions—herein μ_g_—are of sufficient magnitude to enable the isostructural packing arrangement. Thus, we assume that Desiraju’s supramolecular synthons concept^43,53^ is applicable to these dyes with dipole–dipole interactions as the structure-determining force for compounds of very similar shape and equipped with the identical alkyl substituents. II) For such isostructurally packed dyes, the resulting absorption band of the crystalline solid will shift according to the shift of λ_00_ observed for the monomeric dyes in solution. For similarly large μ_eg_ values also the magnitude of the shift will be similar and predictable based on Kasha’s molecular exciton theory considering only Coulomb coupling. III) Shape-complementary dyes with different molecular absorption properties but isostructural supramolecular organization will coassemble in mixtures to give CSSs. Thereby, it will become possible for mixtures of such isostructural dyes to afford aggregation-induced spectral shifts by the Coulomb coupling of their similarly large transition dipole moments (μ_eg_), enabling fine-tuning of absorption maxima in a continuous manner in mixed thin films.

Thus, a series of in total 14 merocyanine dyes were synthesized and characterized, ten of which are novel. First, the push–pull merocyanine dyes **D3(Pyrl)-A1** and **D3(Hex)-A1**, composed of a 2-[4-(*tert*-butyl)thiazol-2(3*H*)-ylidene]malononitrile acceptor (**A1**) and a 2-amino-thiophene donor (**D3**) moiety,^54^ were chemically modified by either changing the heteroatom in their donor heterocycle (**D1** to **D4**) or replacement of their donor amine substituent by less electron-donating substituents (**D5** to **D6**) (Figure 1b). The chosen chemical variations were based on quantum chemical (time-dependent) density functional theory ((TD-)DFT) calculations, predicting both bathochromically (**D1** and **D2**) and hypsochromically (**D4** to **D6**) shifted absorption bands (Figures S2–S3, Table S3). Additionally, for modification of the supramolecular packing structure we synthesized a novel series of dyes bearing butyl chains as donor substituents and a 2-(4-methylthiazol-2(5*H*)-ylidene)-malononitrile acceptor unit (**A2**). The three resulting series of dyes are classified according to their respective solid-state packing arrangements (H-aggregates: **Dx(Pyrl/cPen/OMe)-A1** = H1-type, **Dx(Bu)-A2** = H2-type; J-aggregates: **Dx(Hex)-A1**). All dyes were synthesized by Knoevenagel condensation reaction^55^ and characterized by ^1^H-, ^13^C-NMR, and UV–vis–NIR absorption spectroscopy, electro-optical absorption measurements (EOAM), high-resolution mass spectrometry, differential pulse voltammetry (DPV), and their melting temperature (*T*_melt_). Details on all compounds, syntheses and the corresponding characterizations are given in the Supporting Information.

### Molecular Properties

**Figure 2:**
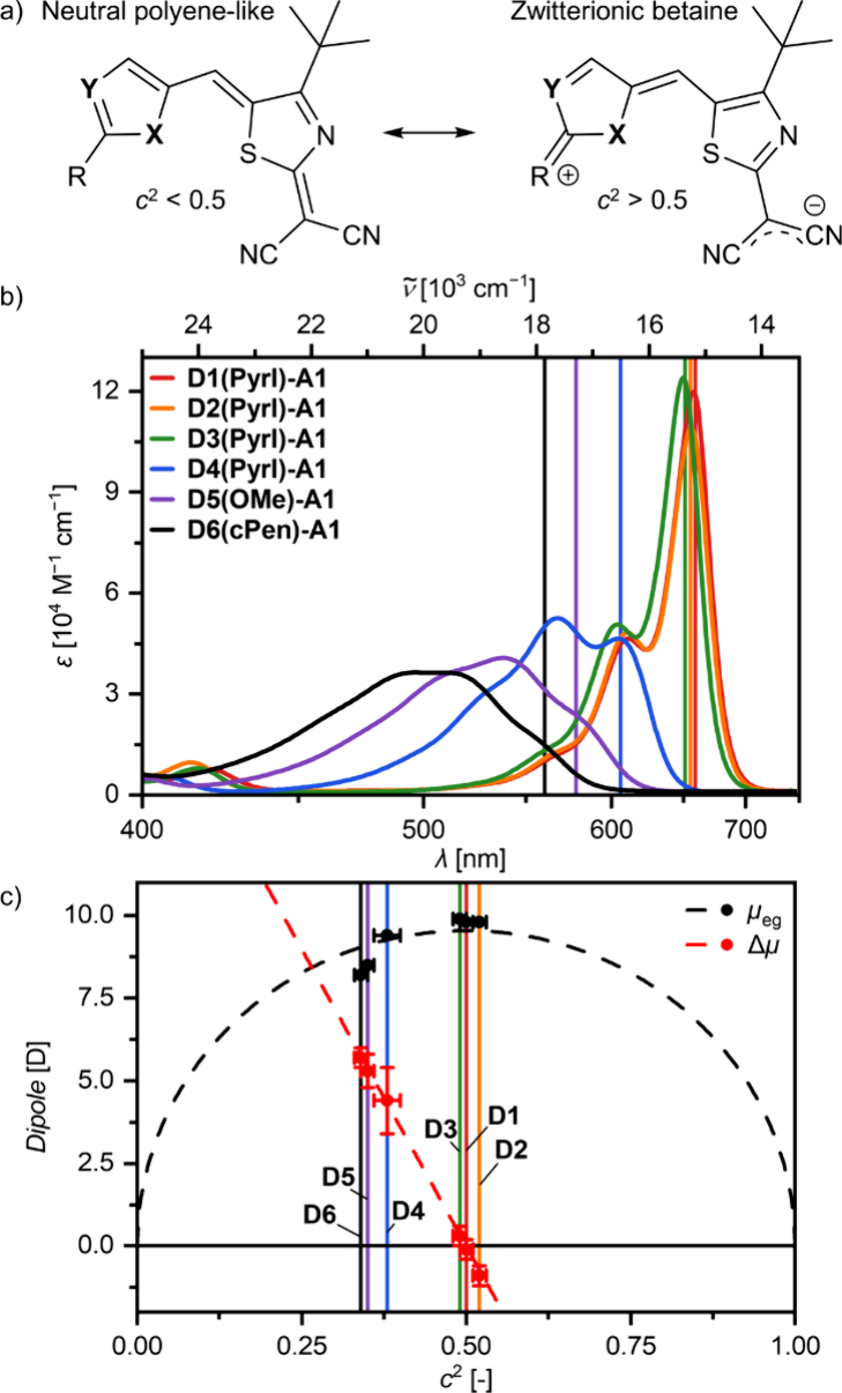
a) Resonance structures of the investigated merocyanine dyes with donor substituents in dependence on *c*^2^. b) UV–vis–NIR absorption spectra of dyes with different donor moieties (**D1** to **D6**) in 10^–5^ M CHCl_3_ solution at 298 K; the colored vertical lines mark the respective *A*_00_ transitions of the S_0_ → S_1_ charge transfer absorption bands. c) Experimental values of μ_eg_ (black) and Δμ (red) in dependency on *c*^2^; the corresponding arbitrarily scaled dashed lines serve as guides to the eye and follow theoretical trends according to ref (58)

The molecular structural and functional properties of the investigated merocyanine dyes can be understood from the resonance structures shown in Figure 2a. Depending on the electron donating strength of the donor unit, such chromophores exhibit a more neutral polyene-like (left structure) or a more zwitterionic betaine-like (right structure) character. To investigate these and the related optical and dipolar properties of the dyes in their molecular state and evaluate the spectral shifts occurring through the chemical donor modifications, UV–vis–NIR absorption spectroscopy (Figure 2b, Table 1) as well as EOAM (Figure 2c, Table S6, Figures S7–S8) in polar CHCl_3_ solutions were carried out. This allows for the determination of μ_eg_, μ_g_, as well as the dipole difference (Δμ = μ_e_ – μ_g_) upon optical excitation, whereby μ_e_ corresponds to the excited-state dipole moment. In turn, this enables the calculation of the resonance parameter *c*^2^, which describes the resonance structure of a merocyanine dye.^16,17,56^

**Table 1:** Spectroscopic and Electronic Properties of Merocyanine Dyes Sorted by Their Respective Solid-State Aggregate Types (H1, H2, or J) in 10^–5^ M CHCl_3_ Solutions at 298 K as well as in Pristine Spin-Coated Annealed Thin Films on Quartz Substrates

	Solution							Annealed thin film^a^			
Dye	λ_00_ [nm]	*ṽ*_00_ [cm^–1^]	*A*_00_/*A*_01_ [-]	ε_max_^b^ [M^–1^ cm^–1^]	μ_g_^c^ [D]	*c*^2^^c^ [-]	μ_eg_^d^[D]	λ_opt_ [nm]	*ṽ*_max_ [cm^–1^]	*fwhm*_opt_^e^ [nm, cm^–1^]	≈*ṽ*_shift_ [cm^–1^]
**D1(Pyrl)-A1**	659	15 175	2.6	119 000	12.4	0.50	9.8	485	20 625	18, 760	+5 450
**D2(Pyrl)-A1**^f^	657	15 225	2.3	108 000	14.3	0.52	9.7	478	20 925	30, 1 300	+5 700
**D3(Pyrl)-A1**	651	15 350	2.5	124 000	13.0	0.49	10.1	477	20 950	19, 810	+5 600
**D4(Pyrl)-A1**	606	16 500	0.9	52 000	9.5	0.38	9.3	464	21 550	29, 1 320	+5 050
**D5(OMe)-A1**^g^,^h^	578	17 300	0.6	40 000	8.7	0.35	8.5	450	22 225	76, 3 575	+4 925
**D6(cPen)-A1**^h^	554	18 050	0.4	36 000	9.8	0.34	8.7	437	22 900	54, 2 770	+4 850
**D1(Bu)-A2**	655	15 275	2.8	119 000	12.4	0.50	9.8	629	15 900	89, 2 275	+625
**D2(Bu)-A2**^f^,^i^	654	15 300	n/a	123 000	14.3	0.52	9.8	n/a	n/a	n/a	n/a
**D3(Bu)-A2**	648	15 425	2.7	137 000	13.0	0.49	10.3	604	16 550	68, 1 925	+1 125
**D4(Bu)-A2**	610	16 400	1.0	54 000	9.5	0.38	9.4	575	17 400	61, 1 850	+1 000
**D1(Hex)-A1**	662	15 100	2.7	120 000	12.4	0.50	9.8	760	13 150	20, 350	–1 950
**D3(Hex)-A1**	652	15 325	3.1	144 000	13.0	0.49	9.9	749	13 350	19, 350	–1 975
**D4(Hex)-A1**	612	16 350	0.9	52 000	9.5	0.38	9.4	706	14 150	23, 475	–2 200
**D6(Hex)-A1**^h^,^j^	554	18 050	0.4	34 000	9.8	0.34	8.2	537	18 625	n/a	n/a

a Annealing temperatures of spin-coated films correspond to 130, 130, and 140 °C for the dye series **Dx(Pyrl/cPen/OMe)-A1**, **Dx(Bu)-A2**, and **Dx(Hex)-A1**, respectively; only **D4(Hex)-A1** was annealed at 170 °C due to its significantly higher *T*_melt_.

b Determined from ≥2 independent measurements with a deviation of <2 000 M^–1^ cm^–1^.

c Values determined by EOAM of dyes **D1(Hex)-A1**, **D2(Pyrl)-A1**, **D3(Hex)-A1**, **D4(Hex)-A1**, **D5(OMe)-A1**, and **D6(Hex)-A1** as representative compounds for their respective donor groups; a more detailed overview of EOAM data is given in Table S6.

d Determined from the experimental monomer spectrum in CHCl_3_ solution.

e The *fwhm*_opt_ values of thin-film spectra are always determined as the full width of the unsymmetric absorption bands.

f Thin film data obtained by spin-coating **D2(Pyrl)-A1** under inert and dark conditions due to photoinstability of the dye; accordingly, thin-film data of **D2(Bu)-A2** is not available.

g Thin films deposited by vacuum sublimation onto substrates kept at 60 °C.

h Monomeric band(-shape) properties in solution determined from Franck–Condon band shape analysis according to Figure S6.

i Optical properties in solution as determined in 10^–5^ M CH_2_Cl_2_ solutions.

j Thin-film aggregate properties not obtainable as **D6(Hex)-A1** does not show J-type coupling.

As exemplarily shown by the UV–vis–NIR spectra of the H1-type dye series, the donor variation induces both a significant spectral shift of the 0–0 transition (*A*_00_) of the lowest energy transition band (total spanned range of 105 nm/2 875 cm^–1^) as well as a change in the overall charge transfer band shape upon variation between **D1** and **D6**. While the *fwhm*_00_ of the first vibronic *A*_00_ transitions just vary by a factor of 2 from ≈730 cm^–1^ (**D1**-**D3**) to ≈1 300 cm ^–1^ (**D4**-**D6**) for chromophores at or below the cyanine limit, respectively, the total *fwhm*_opt_ increases 5-fold between **D1(Hex)-A1** and **D6(Hex)-A1** to ≈4 000 cm ^–1^ (Figure 2b, Table S6). Chromophores with a selenophene (**D1(Pyrl)-A1**) donor unit exhibit the most bathochromic absorption wavelength (λ_00_) at 659 nm, showing an intense and narrow (36 nm, 825 cm^–1^) absorption band with a molar decadic extinction coefficient (ε_max_) of 119 000 M^–1^ cm^–1^. Dyes containing a furan (**D2(Pyrl)-A1**) or thiophene (**D3(Pyrl)-A1**) donor show only a minor hypsochromic shift of λ_00_ with almost analogous monomeric properties to dyes with **D1**. Those with **D2** are not further investigated due to photoinstability of this donor configuration in solution under ambient light in the presence of water. Overall, donors **D1** to **D3** show very similar optical properties, with μ_eg_ values of 9.7–10.1 D as well as intense and narrow charge transfer absorption bands. However, we note a still quite intense vibronic progression (ratio of *A*_00_/*A*_01_ = 2.3–2.6) despite of the realization of an almost perfect polymethine state with *c*^2^ values of 0.49–0.52 and small |Δμ| values of ≤0.9 D determined by EOAM in CHCl_3_. Thus, even at the cyanine limit (*c*^2^ ≈ 0.5), the purity of the absorption band with regard to color selectivity is not ideal.^17,56^

Substituting the donor by a weaker electron-donating thiazole (**D4(Pyrl)-A1**) unit induces a significant hypsochromic shift of the *A*_00_ transition to 606 nm alongside a reduction of the *A*_00_/*A*_01_ ratio from ≥2.3 to 0.9 and of *c*^2^ from around 0.50 to 0.38. As a consequence, the number and intensity of the vibronic progressions is now significantly increased, leading to a rather broad band (≈110 nm, 3 200 cm^–1^) at 606 nm. However, μ_eg_ is only reduced from values of 9.7–10.1 D for **D1**, **D2**, and **D3** to 9.3 D for **D4**. As expected for such a merocyanine dye, the absorption spectra show a bathochromic shift in solvents of increasing polarity (positive solvatochromism, see Figure S5, Table S5).^57^

Upon modification of the thiophene donor heterocycle by replacing the electron-donating amino-substituent with either an alkyloxy (**D5(OMe)-A1**) or aliphatic (**D6(cPen)-A1**) substituent, λ_00_ (*ṽ*_00_) is further hypsochromically shifted to 578 nm (17 300 cm^–1^) and 554 nm (18 050 cm^–1^), respectively. In both cases, the absorption spectra lose their well-resolved vibronic progression structure and show significant band broadening with *fwhm*_opt_ up to ≈4 000 cm^–1^ (Table S6). This occurs due to significant structural changes between the ground and the excited state structures as evident from the large Δμ value upon optical excitation (Figure 2c). The broadened spectra can thus be readily explained by reduced *c*^2^ values of 0.35–0.34, indicating that the dyes drop significantly below the cyanine limit and adopt a more neutral and polyene-like structure.^16,17^ The *A*_00_ transition wavelength was therefore determined by a Franck–Condon band deconvolution of the broad S_0_ → S_1_ absorption band (Figure S6).

As deduced from the prevalence of large ground state dipole moments for the whole series of dyes (Figure 2c), based on the supramolecular synthon hypothesis^43,53^ these isostructural dyes should self-assemble into excitonically coupled solid-state materials with similar solid-state packing arrangements (≈μ_g_^2^) and comparable coupling strengths (≈μ_eg_^2^), with both μ_eg_ and Δμ following a theoretically expected trend in dependence on *c*^2^ (Table S6).^58^ Both the spectral shifts and changes in μ_eg_ linearly correlate to those predicted by quantum chemical calculations (Figures S2–S3, Table S3).

Analogous spectral changes for the monomers in solution are also observed for the other two investigated dye series, **Dx(Hex)-A1** (designed for J-type slipped-stack packing) and **Dx(Bu)-A2** (designed for H2-type cofacial stacking), as the influence of alkyl-substituents at the donor and acceptor moieties on the molecular optoelectronic properties is only marginal. The sterical periphery of the dyes rather impacts the solubility as well as solid-state packing arrangements. An overview of all monomeric absorption properties is given in Table 1 and Figure S4. All optical changes are reflected by the electrochemical redox potentials deduced by DPV in CH_2_Cl_2_ (Figure S9–S10, Table S7). These revealed a shift in the highest occupied (HOMO) and lowest unoccupied molecular orbital (LUMO) energies corresponding to the optical shift of λ_00_. It is noted that the difference between the electrochemically determined bandgap energy (*E*_gap_) and the optical bandgap (*E*_opt_, Figure S11) is only marginal (<0.2 eV, 1600 cm^–1^), as is common for such merocyanine chromophores.^59^

### Pristine Aggregate Properties

**Figure 3:**
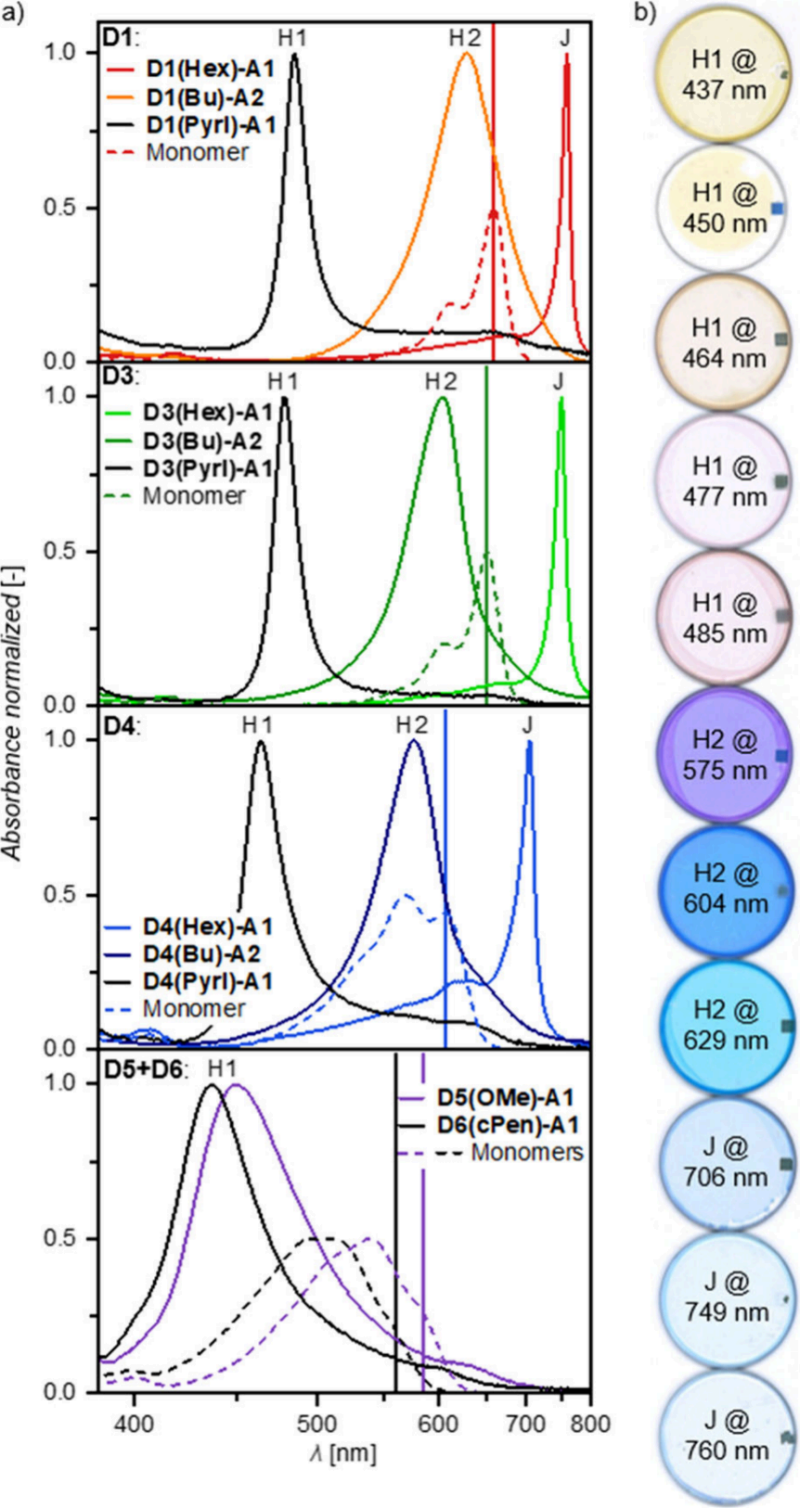
a) Normalized UV–vis–NIR absorption spectra of pristine spin-coated and annealed thin films on quartz substrates sorted by their donor units (**D1** to **D6**); the dashed lines correspond to the respective **Dx** H1-type series monomer absorption spectra in CHCl_3_; the colored vertical lines mark the respective monomeric *A*_00_ transitions. b) Photoscan of the pristine thin films of all narrowband absorbers from Table 1 sorted by their λ_opt_.

To explore the coloristic effect of donor strength in the solid-state, all dyes were deposited as thermally annealed thin films (thickness ≈10 nm)^52^ onto quartz substrates by spin-coating from CHCl_3_ solutions. The annealing temperatures required for an almost complete conversion of the films to the crystalline state were adjusted for each dye individually according to their *T*_melt_ (Table 1, Table S4). This resulted in three series with distinct H- or J-type coupled packing arrangements (H1-, H2-, and J-type) with a significant corresponding blue- or red-shift of the resulting thin-film absorption band, respectively (see Table 1, Figure 3, and Figure S12 for an overview of all pristine thin-film spectral properties).

Within the H1-type aggregate series, all dyes show a narrow and strongly hypsochromically shifted (spectral shift *ṽ*_shift_ between +4 850 and +5 600 cm^–1^) absorption band in their respective thin films (Figure 3a, black lines). The thin-film absorption maxima λ_opt_ of the H1-type band hereby sequentially shift just as the λ_00_ values in solution and range from the light blue at 485 nm (**D1(Pyrl)-A1**) down to the UV at 437 nm (**D6(cPen)-A1**). It is noted, that due to the low solubility of dye **D5(OMe)-A1**, this compound was processed by thermal sublimation in vacuum. This method yields the same distinct absorption bands and is also applicable to the other H1-type dyes. Throughout the H1-type dye series, it is observable that the donor heterocycles **D1**-**D3** provide similar *ṽ*_shift_ values for their H1-aggregates compared to the respective monomer absorption positions (*A*_00_) upon excitonic coupling of around +5 600 cm^–1^, which is reduced to around +5 000 cm^–1^ for donor units **D4** to **D6**. Assuming identical solid-state packing arrangements (*vide infra*), the reduced value of *ṽ*_shift_ can be explained by the reduced μ_eg_ of dyes with **D4** to **D6** (8.5–9.3 D) compared to **D1** to **D3** (9.7–10.1 D), which would account for a correspondingly reduced Coulomb coupling strength within the point-dipole approximation according to Kasha’s theory.^60^ Additionally, minor changes in the overall similar solid-state packing arrangement might also be influencing *ṽ*_shift_. Furthermore, it is observed that a reduction of the *c*^2^ parameter away from the cyanine limit coincides with a broadening of the aggregate absorption bands. While donors **D1** and **D3** with a *c*^2^ of ≈0.50 show the narrowest H1-type bands at about 485/477 nm with *fwhm*_opt_ values of 18–19 nm (760–810 cm^–1^), which is almost identical to the *fwhm*_opt_ of their monomers in solution, the H1-type band is broadened to 29 nm (1 320 cm^–1^) for **D4** (464 nm) with a *c*^2^ of 0.38. The broader absorption band originating from the excitation into higher vibrational states for the monomeric dyes in solution (3 200 cm^–1^ compared to 825 cm^–1^) thus also leads to a broader density of states (DOS) with an increased *fwhm*_opt_ in the solid-state H1-type aggregate.

**Figure 4:**
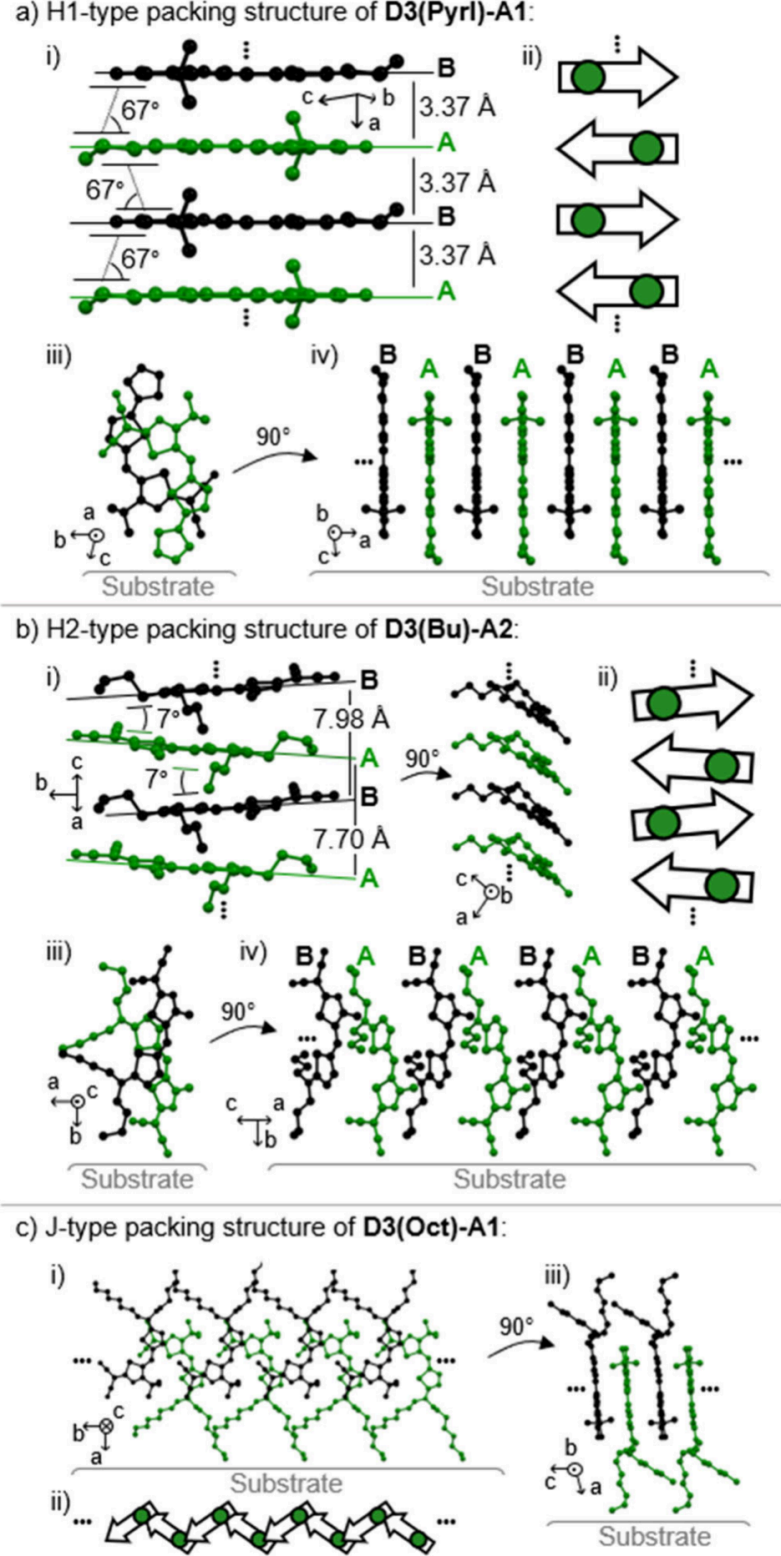
a) H1- and b) H2-type packing arrangements from the single-crystal structures of **D3(Pyrl)-A1** and **D3(Bu)-A2**, respectively; these include (i) a side view of a tetramer π-stack with (ii) a corresponding schematic depiction of the arrangements of μ_g_ (arrows) in this solid-state packing, as well as (iii) a top and (iv) a side view of an individual π-stack in respect to the substrate surface. c) Packing structure of J-type coupled dye **D3(Oct)-A1** showing (i) a top view of an individual J-strand, (ii) a corresponding schematic depiction of the arrangements of μ_g_ in this solid-state packing, and (iii) a side view onto two neighboring J-strands oriented parallel to the substrate surface.

To verify that despite of donor variations identical solid-state packing arrangements prevail for the whole series of dyes, single-crystals of the H1-type dyes (Figure 4a, Figure 6a, Figure S17–S18, Table S10–S13) were successfully grown and compared to those for **D3(Pyrl)-A1**.^54^ The latter was assigned by a combination of thin-film X-ray diffraction (TF-XRD) and selected area electron diffraction (SAED) experiments to be the same polymorph present in the investigated annealed thin films.^54^ All dyes in this series show similar extended 1D π-stacks with equidistant neighboring dyes oriented in an antiparallel manner to cancel out their large μ_g_ values. The individual single-crystals exhibit identical shapes, birefringence, and polarization dependencies in their absorption properties (Figure S20). Thus, all arrangements are fairly isostructural despite a significant difference in their μ_g_^2^ values (76–169 D^2^), which scale linearly with the attractive Coulombic interactions between the dyes. For the cofacially stacked dyes similar π–π distances (*d*_π–π_) of 3.36–3.42 Å and lateral slip angles (θ_slip_) of 62–70° are observed (Table S18). Additional TF-XRD measurements revealed 2θ values of 6.0–7.1° for thin films of all compounds, corresponding to similar out-of-plane lattice distances (*d*_TF-XRD_) of 12.5–14.7 Å (Table S19). This proved that all compounds, in addition to showing identical packing arrangements in the crystalline solid state, also adopt the same orientation on quartz substrates. This behavior perfectly corroborates our first hypothesis, considering the negligible change in the molecular sterical periphery and shape. The reduction of the *c*^2^ parameter from values around 0.50 for **D1** to 0.34 for **D6** is reflected in the single-crystal structures, whereby an inversion of the bond-length alternation pattern especially around the central methine bridge is observed, indicating the shift toward a more polyene-like structure (Figure S21).^56^ As thus demonstrated for the **Dx(Pyrl)-A1** H1-type series, our crystal engineering approach of utilizing specific donor variations at the chromophore core units without modifying the steric environment of the dyes provided both the expected identical packing arrangements in the solid state (proof of hypothesis I) and the predicted similar spectrally shifted λ_opt_ and *fwhm*_opt_ values (proof of hypothesis II).

In a similar vein, isostructural packing arrangements are already suggested by the absorption spectra of the second dye series **Dx(Bu)-A2** (Figure 3a, H2 series). Thin films of this series also revealed H-type coupled absorption bands spanning the visible light range from 629 nm (orange; **D1**) over 604 nm (**D3**) to 575 nm (yellow; **D4**). These absorption bands show smaller spectral shifts compared to the H1-type series (*ṽ*_shift_ ≈ + 1 000 cm^–1^ vs +5 000 cm^–1^) and roughly doubled *fwhm*_opt_ values of ≈2 000 cm^–1^ (Figure 3a, Table 1). A comparative discussion on *ṽ*_shift_ and *fwhm*_opt_ values of the different aggregate series is given later. To elucidate the underlying packing arrangement for this intermolecular coupling, single-crystal analysis of **D3(Bu)-A2** was performed (Figure 4b, Figure S19, Table S17). Thin-film analyses by TF-XRD and SAED confirmed this polymorph to correspond to the H2-type packing arrangement (Figure S22, Table S19). **D3(Bu)-A2** hereby adopts a slightly laterally slipped 1D π-stacking arrangement with μ_g_ vectors oriented in an antiparallel manner, but at an increased *d*_π–π_ of ≈3.88 Å compared to **D3(Pyrl)-A1** with 3.37 Å and a tilt of neighboring π-planes of ≈7° toward each other, resulting in an overall lower and less-pronounced Coulomb coupling (*vide infra*). Analogous to similar single-point TD-DFT calculations previously performed for **D3(Pyrl)-A1**,^54^ it was verified for individual π-stacks from this crystal structure, that this packing arrangement indeed results in H2-type coupled aggregates (details for our calculations are described in the corresponding section in the Supporting Information, Figure S23, Table S20).

Finally, the **Dx(Hex)-A1** dye series with long and flexible aliphatic chains was investigated in spin-coated and thermally annealed thin films (Figure 3a). Correspondingly, as the original dye **D3(Hex)-A1** shows an ultranarrow J-type absorption band at 749 nm with a *ṽ*_shift_ of −1 975 cm^–1^, also dyes **D1**- and **D4(Hex)-A1** show J-type coupling. With similar *ṽ*_shift_ values of around −2 000 cm^–1^, this results in J-bands at 760 and 706 nm for **D1(Hex)-A1** and **D4(Hex)-A1**, respectively. Like for the H1-type series, also here the thiazole substitution (**D4**) with a broadened monomeric absorption in solution leads to broader thin-film absorption bands. This results in *fwhm*_opt_ values of 23 nm (475 cm^–1^) at 706 nm compared to those of the corresponding selenophene- (**D1**, 760 nm) or thiophene-containing (**D3**, 749 nm) dyes with narrower *fwhm*_opt_ of 19–20 nm (≈350 cm^–1^), which is less than half of the *fwhm* of the monomers in solution (≈850 cm^–1^). For **D4(Hex)-A1** we note a significant increase in intensity for higher energy excitonic states, thereby reducing the color purity compared to the aggregates formed by **D1** and **D3** donor units. It is here, however, noteworthy that for **D4(Hex)-A1** the annealing temperature had to be raised from 130 to 170 °C compared to the other two dyes to induce the appropriate J-coupled packing arrangement due to the higher *T*_melt_ of this dye (Table S4).

The packing arrangement of the J-type aggregates was determined from the single-crystal structure of **D3(Oct)-A1**, a novel derivative bearing longer octyl instead of hexyl chains but otherwise identical spectral properties. Details on compound **D3(Oct)-A1** and considerations taken into account for determining the J-type crystal structure are given in the corresponding section of the Supporting Information (Section S10 beginning on page S38). The crystal structure of the J-aggregates revealed a zigzag arrangement of the dimerized chromophores separated as lamellar sheets containing isolated J-type coupled strands (Figure 4c). Unfortunately, the isostructural **D6(Hex)-A1** with the weakest donor moiety, as opposed to its analogue **D6(cPen)-A1**, does not adopt the same exchange-narrowed J-type packing arrangement. Instead, as-cast thin films reveal a very broad and hypsochromically shifted absorption feature at a similar spectral position as the H1-type aggregate, which is changed into broadband amorphous/monomeric absorption band upon thermal annealing (Figure S13). **D6(Hex)-A1** thus reveals the limitation of our dipole–dipole interaction-guided crystal engineering approach. We assume that the sterical demand by the different hybridization of the substituent C atom (sp^3^) compared to the original N atom (sp^2^) is responsible for the change in aggregation behavior. The enhanced entropy through rotational freedom of the substituent presumably allows the hexyl-chains to arrange in a manner, which can facilitate more extended H-type aggregation.

When comparing the three different aggregate series (H1-, H2-, and J-type), large differences in their *ṽ*_shift_ and *fwhm*_opt_ values can be observed. Dyes of the H1-type series show large *ṽ*_shift_ values of ≈+5 000 cm^–1^ and *fwhm*_opt_ values similar to those of their respective monomers in solution of ≈800 cm^–1^ in the blue spectral region. The large *ṽ*_shift_ values can be explained by the tight cofacial π–π-stacking arrangement of the dyes, leading to large Coulomb couplings. Showing two equally close nearest neighbors within the π-stack and thus a resultingly highly symmetric and periodic arrangement, these dyes retain their monomeric *fwhm*_opt_ values also in the aggregated solid state with less contamination from absorption to higher vibrational states. Comparatively, the H2-type aggregates show reduced *ṽ*_shift_ values of ≈+1 000 cm^–1^. These can be allocated to the increased π–π-stacking distances and lateral displacement, resulting in a lower coupling strength. Coincidingly, the H2-aggregates with λ_00_ between 575 and 629 nm show *fwhm*_opt_ values increased to ≈–2 000 cm^–1^. This can be explained by a different orientation (distance, tilt angle) of the two nearest neighbors within the π-stack with only one strongest-coupled nearest neighbor.^61,62^ The H2-type geometry thus reduces the overall coherence length and broadens the resulting spectra. For the J-type aggregates with a zigzag-type arrangement a *ṽ*_shift_ value of ≈–2 000 cm^–1^ is observed but with *fwhm*_opt_ values of ≈400 cm^–1^ for λ_00_ between 706 and 760 nm reduced to less than half of those of the respective monomers in solution (**D1**-**D3**). The J-aggregates show multiple equally coupled nearest neighbors within J-strands which are isolated from each other through the aliphatic donor substituents in the lamellar aggregate structure. The leads to a high coherence length of the aggregate with reduced out-of-stack parasitic coupling, resulting in the narrow *fwhm*_opt_ values. The reduced *ṽ*_shift_ is explained by the increased center-to-center distance of neighboring chromophores compared to the structure of the H1-aggregates.

Thus, based on the first two hypotheses, we could develop three series of isostructural merocyanine dyes composed of identical acceptor heterocycles and different donor moieties affording solid-state color-selective absorbers with λ_opt_ values ranging from the UV (437 nm) to the NIR (760 nm) with narrow *fwhm*_opt_ values below 100 nm (Figure 3). We were able to verify that the packing structures of the aggregates indeed only depend on the chromophore’s sterical demand and not its specific electronic core. Rigid and small substituents with a well accessible π-surface afforded 1D arrangements with H-type coupling (series H1 and H2) through dimer synthons aligned in an antiparallel manner.^43,63^ In contrast, sterically more demanding and flexible substituents at the donor heterocycle prohibited such extended 1D π-stacking and directed the arrangement into slip-stacked J-type coupled aggregates (Figure 4). The *ṽ*_shift_ and *fwhm*_opt_ values of the three aggregate types could be related by the molecular exciton model to their stacking arrangements.

### Mixed Aggregate Properties

**Figure 5:**
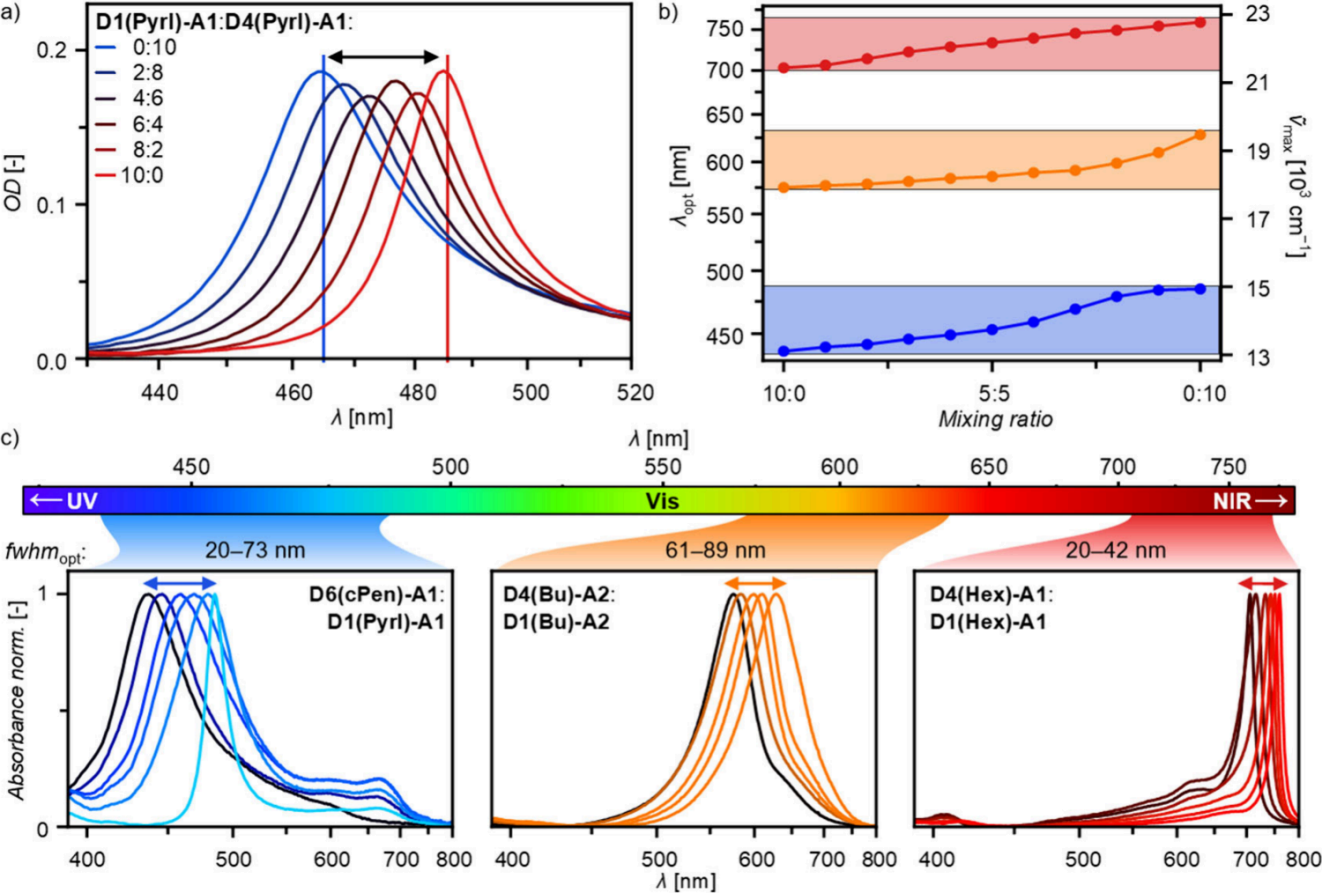
a) Representative UV–vis absorption spectra of spin-coated and annealed mixed thin films of **D1(Pyrl)-A1**:**D4(Pyrl)-A1** on quartz substrates at different molar mixing ratios. b) Thin film absorption band maxima (λ_opt_; *ṽ*_max_) of annealed mixed thin films on quartz substrates in dependence of the mixing ratio; the colored areas highlight those spectral regions which can be tuned through mixing of the two dyes with the furthest apart absorption maxima within the respective dye groups, namely **D6(cPen)-A1**:**D1(Pyrl)-A1** for H1- (blue), **D4(Bu)-A2**:**D1(Bu)-A2** for H2- (orange), and **D4(Hex)-A1**:**D1(Hex)-A1** for J-type dyes (red). c) Normalized UV–vis–NIR aggregate spectra as an overview of all spectral ranges which can be tuned through the mixing of two narrowband absorbing merocyanine dyes with the same color scheme as in b).

For the evaluation of our third hypothesis, mixed aggregates had to be prepared whose absorption maxima should be tunable in a continuous manner just by the mixing ratio of the two utilized dyes in the solid state which have almost identical packing arrangements. While heteroaggregates for photophysical studies can be obtained by covalent linkage^64,65^ or in DNA-templated arrangements^66,67^ as e.g. the well-defined DNA Holliday junctions,^68^ they remain far less explored in the solid state, due to the strong tendency of pigments to phase separate. This hypothesis was based on recent research toward understanding the properties of organic CSSs^69−73^ which, however, has not yet been applied to mixed organic dye aggregates.^74,75^ To address our goal of fine-tunable absorption bands in a continuous manner, we investigated thermally annealed mixed thin films of the isostructural dyes spin-coated from a CHCl_3_ solution (4 × 10^–3^ M). Upon mixing of, e. g., compounds **D1(Pyrl)-A1** (μ_g_ = 14.2 D, μ_eg_ = 9.7 D) and **D4(Pyrl)-A1** (μ_g_ = 10.9 D, μ_eg_ = 9.4 D), a spectral shift of a singular narrow absorption band was observed, whose λ_opt_ continuously shifted between the respective neat λ_opt_ at 485 and 464 nm in dependence on the molar mixing ratio (Figure 5a). This interesting finding excludes a possible scenario of narcissistic phase separation often encountered for crystalline functional materials like in our previous study,^52^ whereby two individual H-type absorption bands would be expected (Figure S14). Instead, by social self-sorting (i.e., mixing),^52,76^ λ_opt_ steadily shifts with the ratio of the mixture, with rather constant *OD* and *fwhm*_opt_ values (Table S8). It is noted that spectral tuning could analogously be achieved for mixtures of the other dyes and for all three aggregate series (H1, H2, and J), and even for a slower solution shearing process (deposition time ≈4 min; Figure S16 and Table S9). The mixed aggregates thus seem to form under thermodynamic and not kinetic control. This interesting observation for dipolar merocyanine dyes allows for a spectral tuning of narrow absorption bands for almost 50% of the visible spectral range with all the while low *fwhm*_opt_ values of <100 nm as is required for OPD applications (Figure 5b-c, Figure S15).

### H-Aggregate (Co-)Crystal Analysis

**Figure 6:**
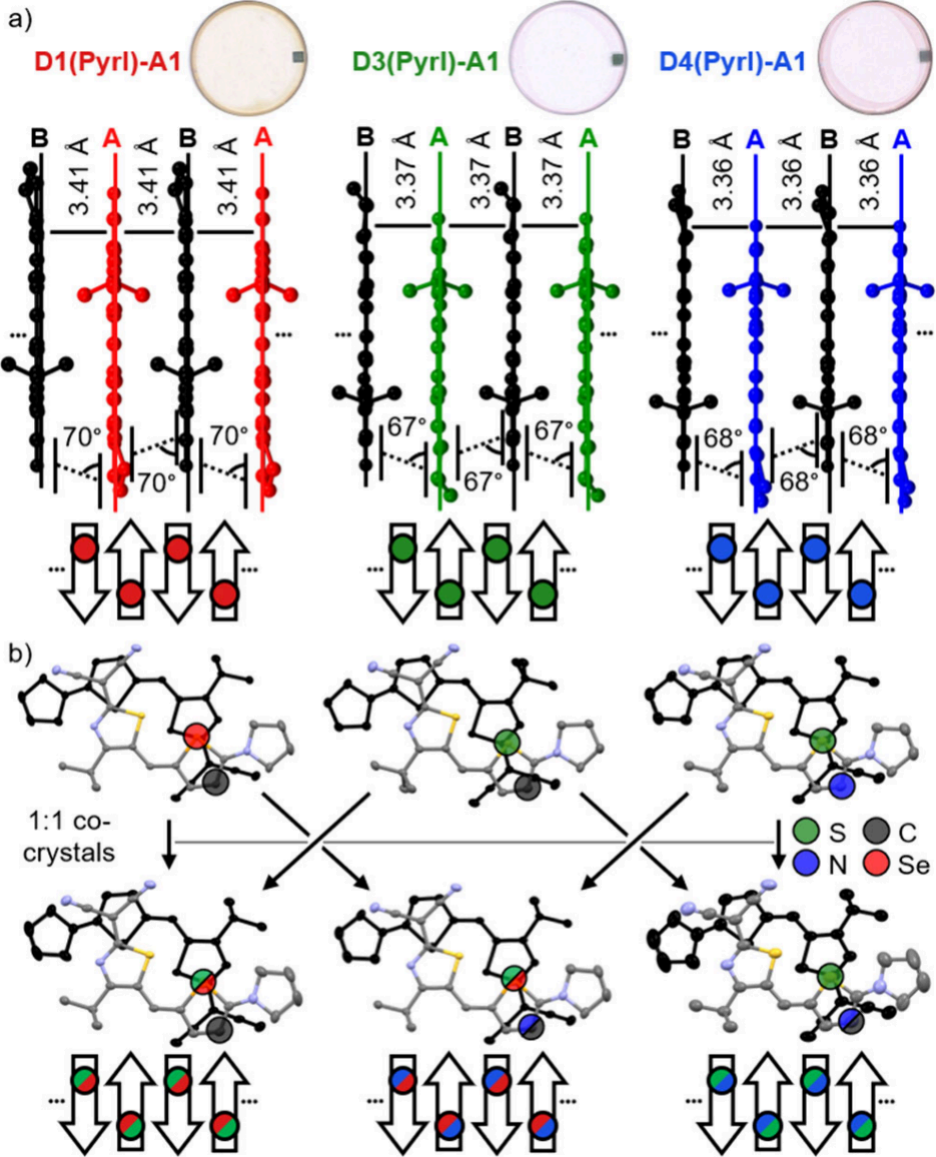
a) Side views onto tetramer π-stacks from the single-crystal structures of **D1**-, **D3**-, and **D4(Pyrl)-A1** including a photoscan of corresponding thin films (top), the measured π–π-distances and slip angles between the donor–acceptor-bridge carbon atoms (middle), and a schematic of the dipolar packing arrangement with μ_g_ represented as arrows (bottom). b) Top view onto dimer units within the π-stack of single-crystals of **D1**-**D4(Pyrl)-A1** (top) and their respective statistical cocrystals (bottom) with thermal ellipsoids set to 50% probability including a schematic of their dipolar packing arrangements; hydrogen atoms and solvent molecules were omitted for clarity; the shaded circles highlight the positions of relevant heteroatom positions within the donor unit, whereas positions marked in one color contain one atom with 100% occupation probability, while those marked in two colors contain two atoms with about 50% each.

The observed tunability of the λ_opt_ of the mixed aggregates formed by social self-sorting^52,76^ is distinct from other previously reported dye-based heteroaggregates. These either demonstrated a splitting into multiple absorption bands upon coupling of chromophores with different frontier orbital levels and μ_eg_ strengths,^65,77,79^ or led to phase separation due to narcissistic self-sorting,^76^ thereby affording absorption spectra composed of the respective contributions from the two homoaggregates.^80^ To thus explain the herein observed spectral tuning for mixed aggregates, we took great efforts to obtain all three possible cocrystals of 1:1 mixtures of **D1**-, **D3**-, and **D4(Pyrl)-A1** by slow diffusion of methanol into an acetone solution thereof. Analogously to their pristine single-crystal structures (Figure 6a), the resulting cocrystals (molar ratio) of all three mixtures reveal an identical packing arrangement into 1D π-stacks (Figure 6b). All pristine and mixed (co)crystals show similar 1D card-stack-like ABAB-type packing with almost identical unit cell parameters (Tables S14–S16, Table S18). Within these binary cocrystals, no specific sequence of the two isostructural compounds is apparent. Instead, the dipolar dyes arrange themselves into completely statistical cocrystals bearing only one single crystalline phase. The statistical nature of these crystals was determined by the electron density at the specific positions of donor heteroatom substitution (C, N, S, Se). In the case of **D3**:**D4(Pyrl)-A1** for example, the electron density of the donor heterocycle S atom is 100%, while that for the CH/N position amounts to 50% for each molecule within the crystal structure. The mixed cocrystals of these structurally similar compounds are thus classified as organic CSSs, which is additionally supported by calculations of crystal lattice energies (Table S22).^81,82^ The latter scale well with μ_g_ as the main driving force for the formation of H-type coupled dimer synthons oriented in an antiparallel manner.^83^ A CSS describes cocrystals with a packing isostructural to the pristine compounds, but with a totally statistical arrangement, enabling a variation of stoichiometry as well as physicochemical properties in a continuous manner.^48,70,71,84^ We refrain from the other common terminology “organic alloy”, as the presence of electrostatic interactions leading to the tunable properties does not fulfill the requirements for an alloy mixture.^85^ The isostructurality of the solid solutions and their respective pristine single-crystals was further verified for all dye series (H1, H2, and J) by additional TF-XRD measurements (Table S19). These conclusively revealed the same *d*_TF-XRD_ of pristine and mixed films in all cases, e. g., with *d*_TF-XRD_ values of 14.5–14.7 Å for the H1-type dye series investigated crystallographically in great detail.

### Transistor and Photodiode Devices

The statistical nature of the CSSs is further supported by p-type charge carrier mobility (μ_p_) measurements in organic thin-film transistor (OTFT) devices using both pristine as well as mixed active layers. As theoretically described by Baranovskii et al.^69^ and also experimentally shown for solid solutions of (fluorinated) zinc phthalocyanines by K. Leo and co-workers,^73^ the mobility of a binary mixed system will reach its minimum when the compositional disorder reaches its maximum at a 1:1 ratio. Accordingly, OTFTs of **Dx(Hex)-A1** dyes, the investigated series with the highest μ_p_, were fabricated on Si/SiO_2_ substrates using a bottom-gate and top-contact architecture (Figure S31, Table S26). Pristine devices hereby result in μ_p_ values of ≈30 × 10^–4^ cm^2^ V^–1^ s^–1^ for all dyes, showing that the lateral mobility is mainly dependent on the equal packing arrangement. Upon mixing of the thiazole- (**D4(Hex)-A1**) and selenophene-containing (**D1(Hex)-A1**) dyes, μ_p_ is reduced to about 6% (1.6 × 10^–4^ cm^2^ V^–1^ s^–1^) for 75:25/25:75 and to 2% (0.6 × 10^–4^ cm^2^ V^–1^ s^–1^) for 50:50 molar mixture ratios. This result coincides with the behavior of related systems reported in literature.^73^

**Figure 7:**
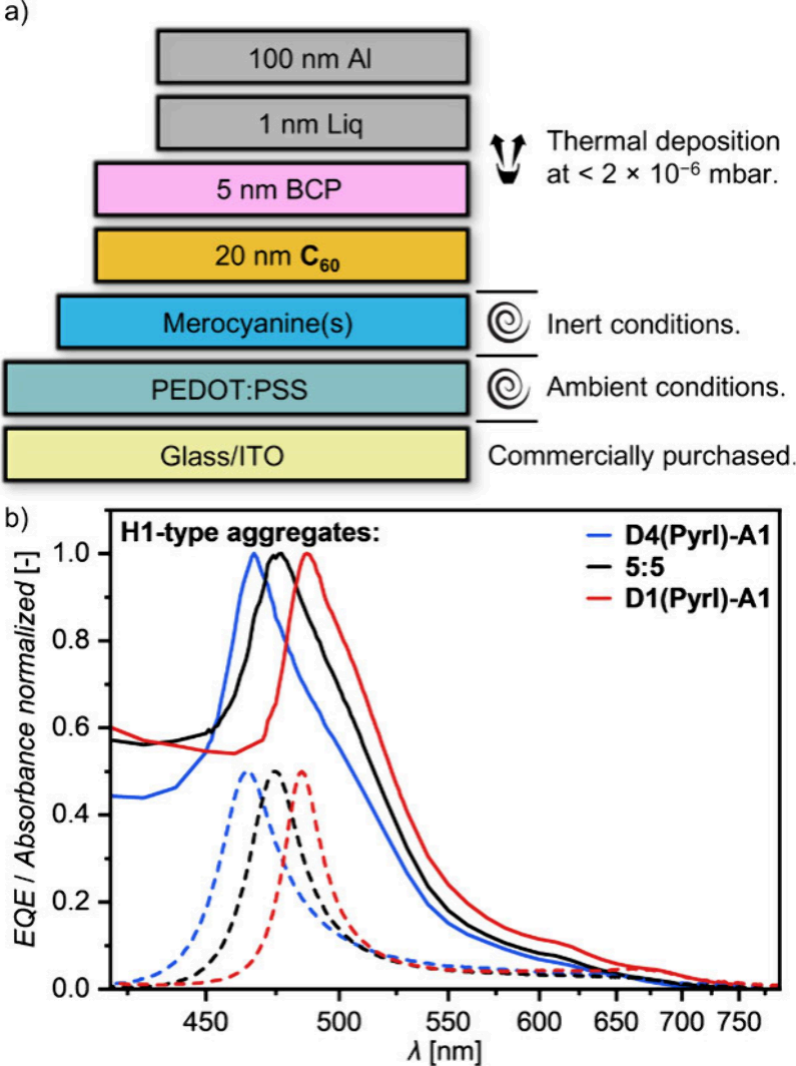
a) Schematic depiction of the employed OPD device architecture. b) Normalized OPD *EQE* spectra (solid lines) compared to the normalized absorption spectra (dashed lines) of the respective pristine/mixed thin films of the H1-type aggregate series.

With regard to applications, narrowband photoresponses are highly desired in OPDs.^86−89^ Due to a lack of suitable narrowband absorbers for the majority of wavelengths, they are often generated in otherwise broadband-absorbing materials through sophisticated device engineering approaches.^5,7,8^ To demonstrate the promising perspectives originating from the herein described spectral tunability of aggregate absorption bands for sensing applications, OPDs with materials with donor units **D1**, **D4**, and mixtures thereof in combination with fullerene **C**_**60**_ as an electron acceptor in a planar heterojunction device architecture were fabricated for all three aggregate series (Figure 7, Figure S32, Table S27). The OPDs all showed color-selective photoresponses of their external quantum efficiencies (*EQE*) at wavelengths closely matching those of the respective thin films with responsivity (*R*_max_) values of 21–88 mA W^–1^. This showcases, that the spectral fine-tuning of molecular as well as solid-state absorption properties can be translated to device application without optical performance losses.

### Extension of Kasha’s Theory for Mixed Solid-State Aggregates

**Figure 8:**
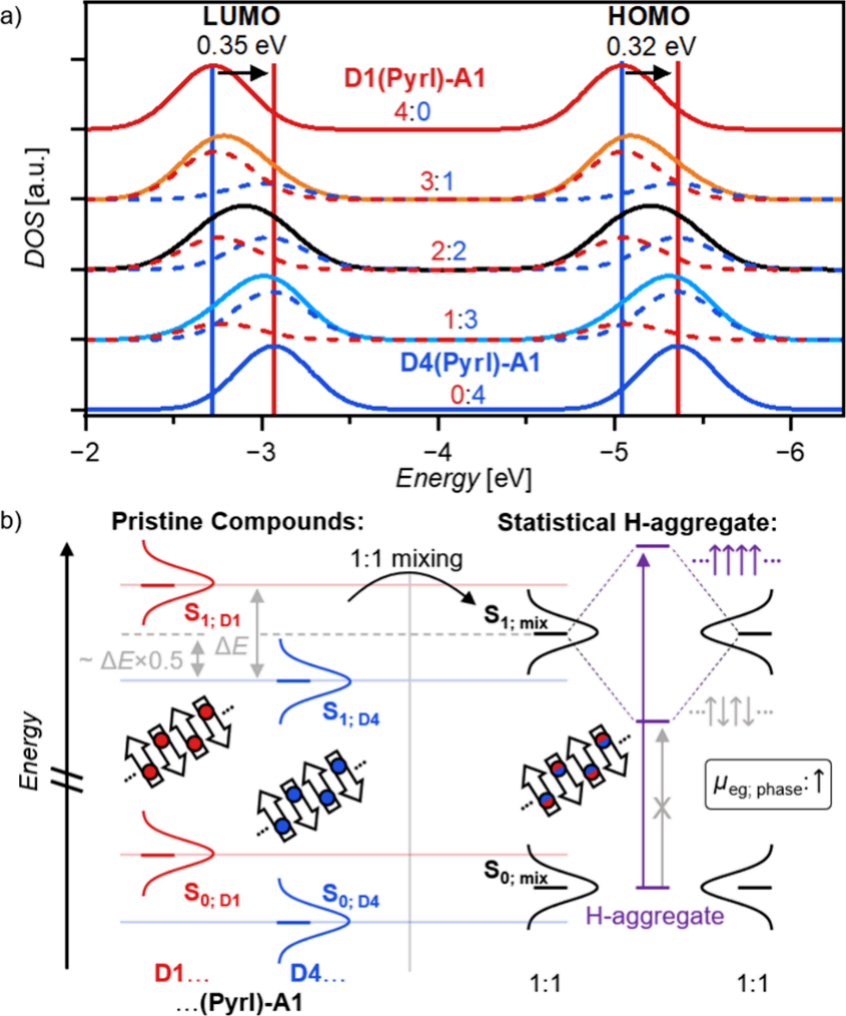
a) Representative energetic positions of HOMO and LUMO levels of tetramer π-stacks of **D1(Pyrl)-A1** (blue) and **D4(Pyrl)-A1** (red) as determined by DFT calculations at the B3LYP/def2SVP level of theory. b) Schematic energy diagram to illustrate the merging (black states) of the pristine ground-state DOS and the formation of solid-solution exciton-coupled aggregate states (violet states) upon mixing of the pristine states of **D1(Pyrl)-A1** (blue states) and **D4(Pyrl)-A1** (red states) at a 1:1 ratio; it is noted that while the state mixing is based on the quantum chemical calculations and previous literature findings,^73^ the splitting into the statistical H1-aggregate states is solely semiempirically based on excitonic coupling within the point-dipole approximation of the Kasha theory.

To rationalize our results for the continuous tunability of absorption bands in mixed aggregates, we devised an extended model of the molecular exciton theory applicable to solid-solution aggregates supported by quantum chemical calculations. Thus, we performed DFT calculations at a B3LYP/def2SVP level of theory for tetramer stacks of **D1(Pyrl)-A1**:**D4(Pyrl)-A1** generated from the single-crystal structure of **D1(Pyrl)-A1** (Figure 8a). Hereby, for each tetramer arrangement, the specific lower (**D1**) and higher energy (**D4**) frontier orbitals were always respectively only localized on one of the two compounds (Figure S25). Accordingly, at each mixing ratio, a mean HOMO and LUMO level for each type of arrangement within the mixed stacks could be determined, resulting in a continuous shift of an overall singular HOMO and LUMO DOS between those of the pristine compounds. This corresponds well to the experimentally observed tunable λ_opt_ values of extended π-stacks within the crystalline thin films. Similar findings for a shift in frontier orbital energy of binary organic mixtures have been described by K. Leo and co-workers.^90,73^

Supported by these theoretical calculations and the experimental spectroscopic results, we thus propose a model explaining the formation of a single excitonically coupled absorption band upon mixing of two isostructural dyes with different frontier orbital energies but similar μ_g_ and μ_eg_ values (Figure 8b). This model is able to explain the spectral fine-tuning upon social self-sorting, which describes the incorporation of one dye into the crystal structure of another, into CSSs simply by Coulomb coupling within the point-dipole-approximation. Based on our calculations, the CSSs always possess only one predominant energy level for each frontier orbital at all mixing ratios. As experimentally shown by UV–vis–NIR spectroscopy, the dyes also possess similar values of μ_eg_ (9.3/9.8 D for **D1**/**D4**). In the statistical binary mixtures, thus Coulomb coupling into a single and strongly optically allowed excited state according to the H-type 1D card-stack arrangement can occur, with its excitation energy in between those of the pristine materials. As upon variation of the mixing ratio the frontier orbital shifts, but the magnitude of the coupling remains rather similar (no change in μ_eg_), the resulting experimentally observed shift of λ_opt_ is obtained, as shown by the experimental optical data of the mixed layers (Figure 5a).

## Conclusion

In this study we were able to demonstrate spectral tunability for thin films of (mixed) solid-state aggregates of dipolar merocyanine dyes from the UV (437 nm) up to the NIR (760 nm) spectral range with *fwhm*_opt_ values down to 760 and 350 cm^–1^ by excitonic coupling at 485 and 760 nm, respectively. For three series of dyes, forming either H- or J-coupled aggregates and each bearing peripheral alkyl substituents with different sterical demands, we could in a first tuning step spectrally shift the monomeric absorption bands by chemical modifications through heteroatom substitution of their chromophore cores. The identical molecular packing arrangement within each aggregate series was next elucidated by single-crystal X-ray crystallography. Spin-coated thin films of these dyes yielded three distinct sets of color-selective aggregate absorption bands in the solid-state, which form irrespective of the chromophore core structure. They are solely dependent on the sterical demand of the alkyl substituents and show analogous spectral shifts as their monomers in solution. Their spectral shifts and bandwidths could be related to the symmetry, coherence length, and number of equal nearest neighbors in their respective aggregate stacking arrangements.

Mixtures of two dyes with isostructural packing arrangements cocrystallized in a statistical fashion, leading to organic crystalline solid solutions without any sign of phase separation. The identical packing arrangement of the pristine as well as the mixed thin films of all H1-aggregate materials could be crystallographically verified. For these materials we could next establish the fine-tuning of the color-selective absorption bands in the solid state. Hereby, the mixed thin films composed of socially self-sorted H- or J-aggregates only showed one singular narrowband absorption feature located between those of its two pristine components. This allowed for a fine-tuning of the aggregate absorption band by simply varying the binary mixture ratio. Supported by quantum chemical calculations, we could explain the observed tuning behavior through a simple semiempirical model based on Coulomb coupling in the point-dipole approximation within Kasha’s molecular exciton model. The spectral tunability of mixed aggregates could be translated to narrowband OPDs, with the devices showing response wavelengths analogous to the absorption maxima of both pristine and mixed merocyanine layers.

We envision that this approach of continuous spectral tuning of (hetero)aggregate absorption bands by minor chemical modification of a chromophore core with an unchanged sterical periphery is widely applicable to a multitude of functional compounds showing distinct excitonic coupling in the solid state. This should allow researchers to develop innovative types of solid-state materials for applications requiring narrowband optical absorption or—by means of simple binary mixing and through supramolecular control—be able to even modify and fine-tune the optical properties of already established material systems.

## Data Availability

Additional data underlying this study are openly available in the Zenodo data repository at 10.5281/zenodo.12818890.

## Supporting Information Available

Experimental methods, synthetic procedures, and characterization of new compounds; additional spectroscopic data; thin-film as well as single- and cocrystal X-ray analyses; quantum-chemical calculations; organic electronics device data (PDF)
