# Supplementary material for: Supramolecular Engineering of Narrow Absorption Bands by Exciton Coupling in Pristine and Mixed Solid-State Dye Aggregates
Source: ACS Cent Sci. 2025 Mar 14;11(3):452–64. doi: 10.1021/acscentsci.4c02157 (PMC11950854; doi:10.1021/acscentsci.4c02157)
Supplement: Supplementary file 1 — oc4c02157_si_001.pdf [file oc4c02157_si_001.pdf]

Supporting Information

Supramolecular Engineering of Narrow Absorption Bands by Exciton Coupling in Pristine and Mixed Solid-State Dye Aggregates

Tim Schembri[1,2], Julius Albert[1], Hendrik Hebling[2], Vladimir Stepanenko[1,2], Olga Anhalt[2], Kazutaka Shoyama[1,2], Matthias Stolte[1,2], and Frank Würthner[1,2,]*

[1]Universität Würzburg, Institut für Organische Chemie, Am Hubland, Würzburg 97074, Germany. E-mail: wuerthner@uni-wuerzburg.de

[2]Universität Würzburg, Center for Nanosystems Chemistry (CNC), Theodor-Boveri-Weg, Würzburg 97074, Germany.

Table of Contents:

1 Investigated Compounds S2

2 Materials & Methods S5

3 Calculations of Molecular Properties S13

4 Molecular & Pristine Solid-State Properties S14

5 Mixed Thin-Film Spectroscopy S22

6 Single-Crystal and Co-Crystal Structures S25

7 Thin Film Diffraction S32

8 Calculated Absorption Spectra of Solid-State Assemblies S33

9 Calculated Frontier Orbitals of Co-Crystals S36

10 Supporting Data for J-Type Crystal Structure S38

11 Organic Electronics Devices S43

12 General Synthetic Schemes S45

13 Precursor Syntheses and Characterization S46

14 Merocyanine Syntheses and Characterization S57

15 Supporting References S70

1 Investigated Compounds

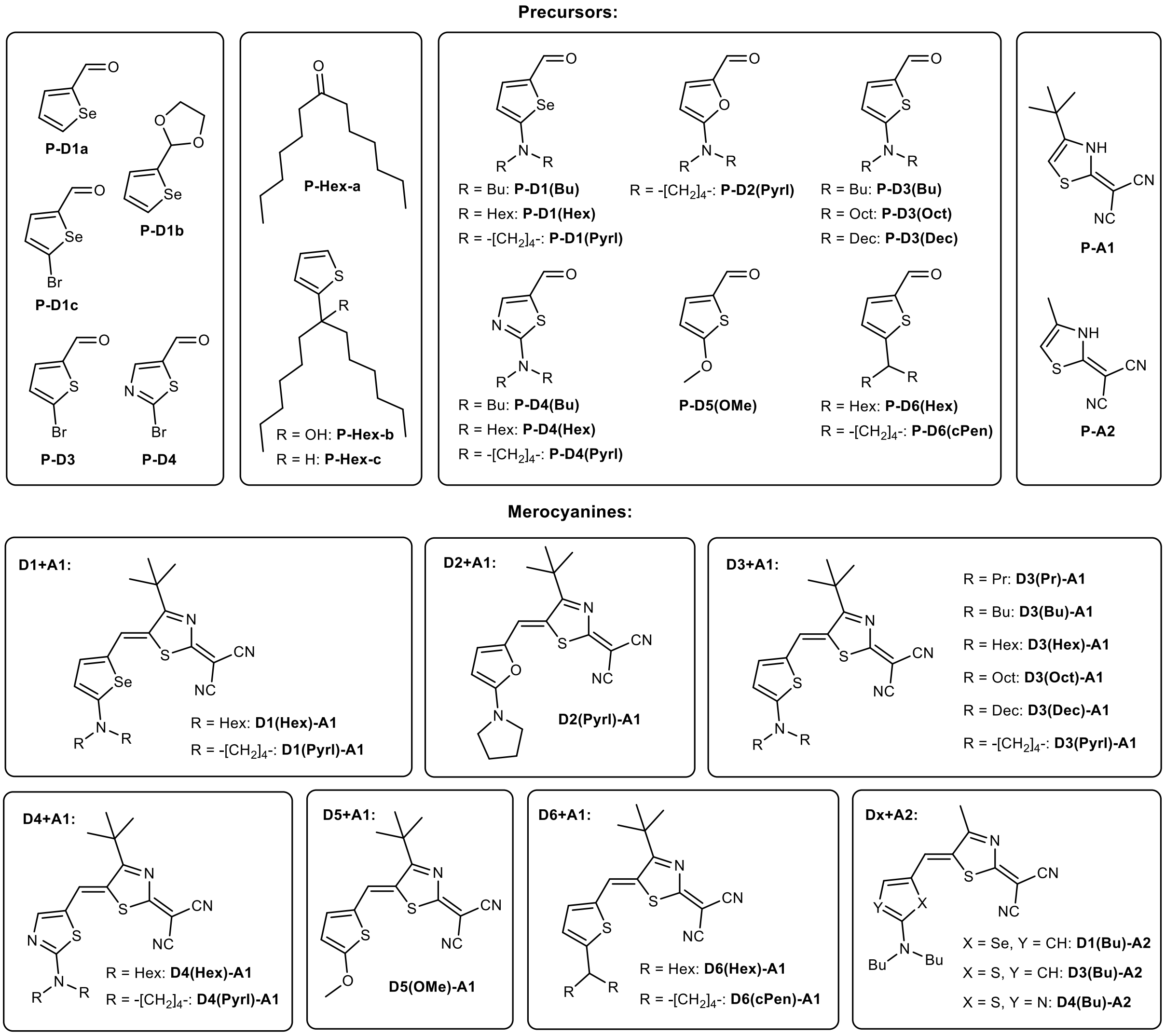

Figure S1. Overview of the chemical structure of all compounds including all merocyanine dyes as well as all synthesized and purchased precursor molecules.

Table S1. Overview of all merocyanine dyes used within this study alongside their synthetic yield, information regarding their novelty, and other information.

Compound	Yield	Novelty	Notes
**D1(Hex)-A1**	92 %	Novel	-
**D1(Pyrl)-A1**	78 %	Novel	-
**D2(Pyrl)-A1**	79 %	Novel	No ^{13}C NMR data available, as the compound is unstable against ambient light in solution.
**D3(Pr)-A1**	60 %	Reported in ref. S1	Synthesized according to literature.
**D3(Bu)-A1**	68 %	Reported in ref. S2	Synthesized according to literature.
**D3(Hex)-A1**	58 %	Reported in ref. S3	Synthesized according to literature.
**D3(Oct)-A1**	65 %	Novel	-
**D3(Dec)-A1**	74 %	Novel	-
**D3(Pyrl)-A1**	43 %	Reported in ref. S1	Synthesized according to literature.
**D4(Hex)-A1**	43 %	Novel	-
**D4(Pyrl)-A1**	31 %	Novel	-
**D5(OMe)-A1**	50 %	Novel	Due to low solubility, the compound was purified by gradient sublimation.
**D6(Hex)-A1**	6 %	Novel	-
**D6(cPen)-A1**	70 %	Novel	-
**D1(Bu)-A2**	75 %	Novel	-
**D3(Bu)-A2**	56 %	Reported in ref. S4	Compound fully characterized, as no detailed characterization and synthetic data was available in literature.
**D4(Bu)-A2**	46 %	Novel	-

Table S2. Overview of all precursor compounds used within this study alongside their synthetic yield, information regarding their novelty, and other information.

Compound	Yield	Novelty	Notes
**P-D1a**	-	-	Commercially available.
**P-D1b**	94 % [1)]	Novel	Compound not purified. Due to reversible nature of the reaction, significant amounts of **P-D1a** are still present in product mixture.
**P-D1c**	69 %	Reported in ref. S5	Synthesized using a novel synthetic route. Literature yield: n/a.
**P-D3**	-	-	Commercially available.
**P-D4**	-	-	Commercially available.
**P-Hex-a**	-	-	Commercially available.
**P-Hex-b**	62 %	Novel	Reaction procedure adapted from the synthesis of analogous compound bearing -Pr instead of -Hex substituents from ref. S6.
**P-Hex-c**	29 %	Novel	-
**P-D1(Bu)**	68 %	Novel	-
**P-D1(Hex)**	83 %	Novel	-
**P-D1(Pyrl)**	91 %	Reported in ref. S7	Synthesized according to literature.
**P-D2(Pyrl)**	-	-	Commercially available.
**P-D3(Bu)**	69 %	Reported in ref. S7	Synthesized according to literature.
**P-D3(Oct)**	34 %	Reported in ref. S8	Synthesized according to literature. Literature yield: n/a.
**P-D3(Dec)**	36 %	Novel	-
**P-D4(Bu)**	88 %	Novel	-
**P-D4(Hex)**	74 %	Reported in ref. S9	Synthesized using a novel synthetic route. Literature yield: 63 %.
**P-D4(Pyrl)**	-	-	Commercially available.
**P-D5(OMe)**	67 %	Reported in ref. S10	Synthesized according to literature.
**P-D6(Hex)**	72 %	Novel	-
**P-D6(cPen)**	-	-	Commercially available.
**P-A1**	87 %	Reported in ref. S2	Synthesized according to literature.
**P-A2**	85 %	Reported in ref. S11	Synthesized according to literature.

[1)] Total yield of product mixture roughly containing a 2.5:1.0 ratio of **P-D1b**:**P-D1a** according to the ^{1}H NMR spectrum shown in Figure S34, which would accordingly roughly amount to a yield of 67 % for **P-D1b**.

2 Materials & Methods

Synthesis and purification:

All reactions were performed in standard glass equipment. Chemicals and solvents were purchased from chemical suppliers and used without further purification. Anhydrous diethyl ether was obtained from a *Pure Solv MD-5* solvent purification system (*Innovative Technology*). An overview of all precursors and merocyanine chromophores used and / or synthesized is given in Figure S1, Table S1 and Table S2. All synthetic procedures are described in detail in the respective part of the synthesis section. Column chromatography was performed using either glass columns of different sizes packed with silica gel 60 M (particle size: 0.04 to 0.063 mm from *Merck*) or using a *puriFlash® 450* flash chromatography system (*interchim® Inc.*) equipped with pre-packed HP-silica (particle size: 0.03 mm) columns. Thin layer chromatography was performed using pre-coated *ALUGRAM® Xtra SIL G/UV254* chromatography sheets with a layer thickness of 0.2 mm (*MACHEREY-NAGEL GmbH & Co. KG*). Due to its low solubility, compound **D5(OMe)-A1** was additionally purified by thermal gradient sublimation at a base pressure of ~ (1–10) × 10^{-6} mbar using a custom-built gradient sublimation setup.

NMR (nuclear magnetic resonance) spectroscopy:

^{1}H NMR and proton decoupled ^{13}C NMR spectra were recorded using a *Bruker Avance III HD 600* or *Avance III HD 400* spectrometer at 295 K. Chemical shifts (δ) are stated in parts per million (ppm) relative to the residual undeuterated solvent signal[S12] and coupling constants (J) in Hz. The following abbreviations were applied to describe signal multiplicities: s = singlet, d = doublet, t = triplet, quin = quintet, sext = sextet, and m = multiplet. For a correct assignment of 1D NMR peaks, if required, additional 2D NMR ^{1}H ^{1}H-COSY (^{1}H ^{1}H correlation spectroscopy), HSQC (heteronuclear single-quantum correlation), or HMBC (heteronuclear multiple-bond correlation) spectra were recorded.

High-resolution mass spectrometry:

High-resolution matrix-assisted laser desorption/ionization time-of-flight (MALDI-TOF) spectra were recorded using a *UltrafleXtreme* from *Bruker Daltonics GmbH* using *trans*-2-[3-(4-*tert*-butylphenyl)-2-methyl-2-propenylidene]malononitrile as matrix material. The matrix and a $CHCl_3$ or CH_2Cl_2 solution of the compound ($c_0 \approx 1$ mg mL^{-1}) were mixed in a 3:1 volume ratio and then co-deposited. High-resolution direct insertion probe (DIP) spectra were recorded using a *micOTOF-Q III* from *Bruker Daltonics GmbH*. MALDI-TOF spectra were used to characterize merocyanine dyes, while DIP mass spectrometry was used to characterize the lower molecular weight precursors.

Melting points:

Melting points (T_{melt}) were determined with a *Stuart® SMP50* digital automatic melting point apparatus. All given melting points are reported uncorrected.

Differential scanning calorimetry (DSC):

Thermal analyses by DSC were performed using a *PerkinElmer DSC 8000* equipped with a *PerkinElmer Intracooler 2* cooling system. The measurements were conducted in Aluminum pans at heating and cooling rates of 5 K min^{-1}.

Solution UV-Vis-NIR absorption spectroscopy:

UV-Vis-NIR absorption spectroscopy in solution was conducted using a two-beam *Jasco V770* UV-Vis-NIR spectrophotometer. All solution spectra were measured in 10 mm quartz cuvettes (*SUPRASIL®*, *Hellma® Analytics*) using spectroscopic grade solvents at 10^{-5} M and at 298 K.

Electro-optical absorption measurements (EOAM):

Dipole moments of the ground state μ_g and the dipole moment differences $\Delta\mu = \mu_e - \mu_g$ (μ_e = excited state dipole moment) of chromophores were determined by EOAM with a home-built instrument.[S13] The single beam spectrometer is composed of a Xenon lamp as light source in combination with a double monochromator (*Horiba Jobin Yvon*) followed by a Hanle depolarizer as well as a Glan polarizer to obtain linear polarized light with appropriate spectral bandwidth ($\Delta\lambda < 1$ nm). This polarized light passes through a sample solution ($OD \leq 1.2$) in a stainless-steel sample cell, with two flat electrodes at a distance of about 3 mm. The difference of absorption of a solution with ($\varepsilon^E(\varphi,\tilde{\nu})$) and without ($\varepsilon(\tilde{\nu})$) an externally applied electric field **E** was measured with light parallely ($\varphi = 0°$) and perpendicularly ($\varphi = 90°$) polarized to the direction of **E**.[S14-S15] For uniaxial phases, induced in a solution by both an alternating and a constant electric field of about 3×10^6 V m^{-1}, the dichroism "$\varepsilon^E(\varphi,\tilde{\nu}) - \varepsilon(\tilde{\nu})$" depends on the orientational order of the molecules due to their ground state dipole moment μ_g, the shift of the absorption band proportional to the dipole moments difference $\Delta\mu$, and on the electric field dependence of the electric transition dipole moment $\mu_{eg}(\mathbf{E})$. The linear combination L_p^{tu}, corrected by the contribution of the first ($t(\tilde{\nu})$) and second ($u(\tilde{\nu})$) derivative of the UV-Vis-NIR spectrum, should be independent of $\tilde{\nu}$ and proportional to μ_g^2 in case of a uniformly polarized transition band. UV-vis-NIR spectra, required for the evaluation of the integral absorption (μ_{eg}^2) as well as the determination of $t(\tilde{\nu})$ and $u(\tilde{\nu})$, were recorded with a *Perkin-Elmer Lambda 950* spectrophotometer at 298 K. All EOA measurements were carried out in anhydrous $CHCl_3$.

Differential pulse voltammetry (DPV):

DPV was performed using a *BASi Epsilon*™ potentiostat under ambient conditions. A Pt disc electrode and a Pt wire electrode were used as working and counter electrode, respectively, while an AgCl/Ag electrode acted as a reference electrode. The measurement was conducted in 2×10^{-5} M solutions with tetrabutylammonium hexafluorophosphate ((n-Bu)$_4$NPF$_6$; 0.1 M) as an electrolyte and ferrocene as the internal standard. As solvent, CH_2Cl_2 dried with a commercial solvent purification system *PS-M6-6/7-En* from *inert technologies* was used. All sweeps were performed from negative to positive voltages in

increments of 0.004 V with a scanning rate of 100 mV s^{-1}. Frontier orbital values were calculated by calibrating the data to the ferrocene/ferrocenium (Fc/Fc^+) couple of −5.15 eV.[S16,S17,S18]

Spin-coating:

Spin-coated thin films were deposited under ambient conditions onto quartz plates (*SUPRASIL*®, *Hellma*® *Analytics*) from a $CHCl_3$ (anhydrous grade, *Sigma Aldrich*®) solution at 4×10^{-3} M for mixed film studies and 1.5 mg mL^{-1} pristine film studies. The films were deposited using 200 µL of solution and a static dispense method with 3 333 rpm s^{-1} and 1 000 rpm for 30 s. Subsequentially, all thin films were thermally annealed on a precision hot plate (PZ28-2, *Harry Gestigkeit GmbH*).

Vacuum thin-film deposition:

For thermal sublimation procedures an *OPTIvap-XL* system (*CreaPhys GmbH*) equipped with 6 MHz sensing crystals (*umicore*) was used. All sublimated merocyanines were previously purified by gradient sublimation and then deposited at a base pressure of $< 2 \times 10^{-6}$ mbar with sublimation rates of 0.05 Å s^{-1} and a general estimate density of 1.33 g cm^{-3}. The ratio in co-sublimated thin films was adjusted using the mass flow measured by two independent sensing crystals.

Thin-film UV-Vis-NIR absorption spectroscopy:

UV-Vis-NIR absorption spectra of thin films were recorded in transmission mode using a *PerkinElmer Lambda 950* spectrophotometer equipped with a 150 mm integration sphere. The initial light beam was depolarized using the spectrometer's internal common beam depolarizer and, when measuring solution-sheared thin films, re-polarized using an integrated mechanically-controlled polarizer.

Single- and co-crystal growth:

All single- and co-crystals for crystal structure determination were grown from 1 mg mL^{-1} acetone solutions using the solvent diffusion method with methanol as a counter solvent. Prior to crystallization, the solutions were filtered through 0.21 µm hydrophobic polytetrafluoroethylene syringe filter (*Kinesis group*). For co-crystals, a 1:1 (molar ratio) solution was used. The only exceptions were **D4(Pyrl)-A1**, which was grown with *n*-hexane instead of methanol as counter solvent, and **D3(Bu)-A2**, which was grown from $CHCl_3$ with *n*-hexane as counter solvent using the vapor diffusion instead of the solvent diffusion method.

For single-crystal growth on a surface, cleaned quartz plates (*SUPRASIL*®, *Hellma*® *Analytics*) or Si/SiO_2 wafers were used as substrates. The wafers were cleaned by successively rinsing them with toluene, acetone, and isopropanol, which was followed up by a 30 min UV/ozone treatment (*UVO-Cleaner*®, *Jetlight Company Inc.*). The substrates were covered with a stock solution of the respective dye at 1 mg mL^{-1} in $CHCl_3$ (filtered using a 0.21 µm hydrophobic polytetrafluoroethylene syringe filter, *Kinesis group*). Crystal growth occurred in a sealed container with 400 µL of methanol as counter

solvent for 24 h, after which the container was opened and residual $CHCl_3$ left to evaporate under ambient conditions.

Single- and co-crystal X-ray diffraction (XRD):

XRD measurements for the determination of single- and co-crystal structures were performed either using a *Bruker D8 Quest Kappa* diffractometer with a *Photon II* detector using $Cu_{K\alpha}$ radiation (λ = 1.5406 Å) at 100 K (for **D1(Pyrl)-A1**, **D3(Pyrl)-A1**:**D1(Pyrl)-A1**, **D4(Pyrl)-A1**:**D1(Pyrl)-A1**, **D6(cPen)-A1**, **D3(Bu)-A2**, and **D5(OMe)-A1**) or at the *P11* synchrotron beamline at *DESY* (for **D4(Pyrl)-A1** and **D3(Pyrl)-A1**:**D4(Pyrl)-A1**). The synchrotron diffraction data were collected by a single 360° ϕ scan at 100 K and then indexed, integrated, and scaled using the XDS program package.[S19] The structures were solved using SHELXT,[S20] expanded with Fourier techniques and refined using SHELXL.[S21] All non-hydrogen atoms in the main residue were refined anisotropically. Constraints and restraints using standard SHELX commands (RIGU, DELU, ISOR, CHIV, SIMU, SAME, DFIX, DANG, EADP, and SADI) were applied to stabilize the refinement around disordered moieties and solvent molecules. Crystallographic data for the structures reported in this Article have been deposited at the *Cambridge Crystallographic Data Centre* (*CCDC*) under deposition numbers CCDC 2362598 (**D1(Pyrl)-A1**), 1496525 (**D3(Pyrl)-A1**),[S1] 1496527 (**D3(Pr)-A1**),[S1] 2362602 (**D3(Oct)-A1**), 2362601 (**D3(Bu)-A2**), 2362610 (**D4(Pyrl)-A1**), 2362599 (**D5(OMe)-A1**), 2362600 (**D6(cPen)-A1**), 2362603 (**D1(Pyrl)-A1**:**D3(Pyrl)-A1**), 2362604 (**D1(Pyrl)-A1**:**D4(Pyrl)-A1**), and 2362611 (**D3(Pyrl)-A1**:**D4(Pyrl)-A1**). Copies of these data can be obtained free of charge from the *CCDC* via http://www.ccdc.cam.ac.uk/structures/.

Thin-film X-ray diffraction (TF-XRD):

TF-XRD measurements were collected on a *Bruker D8 Discover* diffractometer with a *LynxEye-1D* Detector and $Cu_{K\alpha}$ (λ = 1.5406 Å) radiation. The measurements were performed with 40 kV × 40 mA power using a 0.6 mm beam slit. The diffractograms were referenced against blank quartz substrates.

Transmission electron microscopy (TEM) and selected area electron diffraction (SAED):

TEM images and SAED patterns were obtained with an *FEI Titan 80-300* transmission electron microscope operated at 300 kV. The thin films for the TEM/SAED experiments were prepared by depositing the merocyanine layers onto glass substrates coated with PEDOT:PSS (= poly(3,4-ethylenedioxythiophene):polystyrene sulfonate). The PEDOT:PSS films were prepared by spin-coating a colloidal suspension of PEDOT:PSS in H_2O with a solid content of 1.3–1.7 % (PEDOT:PSS 1:6 weight ratio, *Heraeus*). The suspension was ultrasonicated at room temperature for 30 min and filtered through a 0.45 µm hydrophilic polyvinylidene fluoride syringe filter (*Kinesis group*) prior to spin-coating (*POLOS*™, *SPS-Europe spin-coater*) in a lamellar flowbox (*WIBO barrier® BAKVO 180/91*, *Weisstechnik*). The substrates were covered with the suspension and spin-coated at 2500 rpm with

4000 rpm s^{-1} acceleration for 30 s using a static dispense method. The substrates were then thermally annealed on a precision heating stage (*Harry Gestigkeit GmbH*) for 30 min at 240 °C. The organic layers were then deposited either by thermal sublimation or by spin-coating a 1.5 mg mL^{-1} solution from $CHCl_3$ (solution volume: 120 µL; static dispense method; acceleration: 3000 rpm s^{-1}; spin speed: 3000 rpm; spin duration: 30 s; thermal annealing: 130 °C for 5 min). Afterwards, the substrates were immersed in purified H_2O and the floating merocyanine film was taken from the water with carbon-covered copper grids for TEM (lacey carbon films on 200 mesh Copper grids, *Agar Scientific Ltd.*). It was verified by UV-Vis-NIR absorption spectroscopy that the films show the desired and identical aggregate absorption properties before and after immersion in H_2O.

Polarized optical microscopy (POM):

POM was conducted using a *Carl Zeiss Axio Imager A2m* optical polarization microscope equipped with a *Carl Zeiss* halogen lamp (380059-1660-000) and an *Ocean Optics MAYA2000-Pro* diode array spectrometer. Polarized absorption spectra at different angles were recorded with fixed polarizer and analyzer positions by rotating the substrate to avoid parasitic optical losses.

Calculations for estimation of the shift in absorption upon donor substitution:

Quantum chemical density functional theory (DFT) and time-dependent DFT (TD-DFT) calculations for estimation of the shift in absorption upon changing the donor moiety of merocyanine dyes bearing the **A1** acceptor unit and different donor units **D1-D6** with only methyl (Me) substituents for reduced computational effort were performed using the *Gaussian 09* program package[S22] with the hybrid exchange–correlation functional CAM-B3LYP by Handy and co-workers[S23] and the valence double-zeta with polarization basis set 6-31G(d).[S24] The molecular structures were optimized with a DFT calculation and the excited state absorption properties successively calculated with a TD-DFT calculation. For the latter, the molecular charges were fit to the electrostatic potential according to the Merz-Singh-Kollman scheme with an additional constrain to reproduce the dipole moment.[S25]

Amsterdam Density Functional (ADF) calculations:

ADF was used to calculate the effective hole and electron transfer integrals t_h and t_e, respectively, between nearest neighbors in π-stacks of **D3(Bu)-A2** in its single-crystal structure for an estimation of the possible charge-transfer coupling J_{CT}.[S26,S27] Therefore the ADF program[S28,S29] with the PW91 functional[S30] and a TZP basis set[S31] within the unique fragment approach[S32] was used. The t_h was then calculated according to ref. S33.

Mixed-stack frontier orbital calculations:

To study the energetic shift of the highest occupied molecular orbital (HOMO) and lowest unoccupied molecular orbital (LUMO) upon mixing structurally similar merocyanines into solid solution aggregates, DFT single-point calculations with the B3LYP[S34,S35] functional and the def2SVP[S36] basis set

were performed on mixed **D4(Pyrl)-A1**:**D1(Pyrl)-A1** stacks of four merocyanines. The stacks were constructed by taking a π-stacked arrangement of four **D1(Pyrl)-A1** molecules from its single-crystal structure and manually changing the necessary atoms to form the required **D4(Pyrl)-A1** molecules (see Figure S25 for an overview of all stack arrangements used as well as the corresponding calculation results). In these model geometries, the Kohn-Sham energies of the HOMO / LUMO orbitals of **D4(Pyrl)-A1** only differ by < 0.07 / 0.09 eV compared to those calculated from the actual single-crystal structure of **D4(Pyrl)-A1**. This demonstrates that the manual replacement of atoms along with the coinciding small geometrical distortion of the **D4(Pyrl)-A1** molecules does not significantly influence their orbitals. For each fixed molecular ratio in the stack (4:0, 3:1, 2:2, 1:3, 0:4), the first four HOMO and LUMO orbitals were allocated to **D4(Pyrl)-A1** or **D1(Pyrl)-A1** according to their position in the four-stack. The resulting mean individual HOMO and LUMO energies were scaled according to the mixing ratio and convoluted with a Gaussian broadening of 500 meV, to yield the DOS of the individual chromophore types within the stacks. These were then summed up, to yield the total DOS of the solid solution or pristine dye stack. These calculations were performed following similar calculations by Leo *et al.* performed on **F_xZnPc**:**ZnPc** solid solutions.[S37]

Lattice energy (E_{latt}) calculations:

Calculations of E_{latt} were performed using the *CrystalExplorer* software (https://crystalexplorer.net/) at a CE-B3LYP/6-31G(d,p) level of theory.[S38] Therefore a cluster of atoms within a 30 Å radius around a center molecule was generated and all fragmented molecules belonging to these atoms then completed. The interaction between the center molecule and all its surrounding molecules was calculated as the sum of electrostatic, polarization, dispersion, and exchange repulsion interactions. All interactions were scaled according to factors optimized by Spackman and co-workers for the wavefunction at this level of theory.[S39] The E_{latt} could then be calculated as half the sum of all these interactions.

Organic thin-film transistor (OTFT) fabrication & characterization:

OTFTs were fabricated and characterized under inert conditions (*M.Braun Inertgas Systeme GmbH*, *UNIlab Pro*, $c(O_2) < 1$ ppm, $c(H_2O) < 1$ ppm) using Si/SiO_2 wafers as substrates. The wafers were cleaned by successively rinsing them with toluene, acetone, and isopropanol, which was followed up by a 30 min UV/ozone treatment (*UVO-Cleaner®*, *Jetlight Company Inc.*). The active layer was spin-coated from a $CHCl_3$ (anhydrous grade, *Sigma Aldrich®*) solution at 4×10^{-3} M using a static dispense method with 3 333 rpm s^{-1} and 3 000 rpm for 30 s. Subsequentially, all thin films were thermally annealed on a precision hot plate (PZ28-2, *Harry Gestigkeit GmbH*). The top Au (99.99 %, *umicore*) electrode was evaporated in an *OPTIvap-XL* (*CreaPhys GmbH*) thermal evaporation system equipped with 6 MHz sensing crystals (*umicore*) at a rate of 0.1 to 0.3 Å s^{-1} and a pressure of $< 5 \times 10^{-6}$ mbar using a shadow mask resulting in OTFTs with 200 × 200 μm² contacts and 25 μm channel length.

Prior to measurement, individual OTFTs were isolated from each other by removing the organic semiconductor layer around the device with the needle of a micromanipulator. Transfer characteristics were recorded from +10 V to −50 V with 400 mV increments using an *Agilent 4055C* semiconductor parameter analyzer and a *Cascade EPS150* probe station. Mobility data was determined in the saturation regime and averaged over at least ten devices across two substrates.

Organic photodiode (OPD) fabrication & characterization:

For OPD fabrication, 2.5 × 2.5 cm^2 glass/ITO (*Soluxx GmbH*, sheet resistance of 15 Ω □$^{-1}$, 16 mm diameter circular ITO pattern) substrates were used. The substrates were cleaned by ultrasonication (*VWR Ultrasonic Cleaner*) in acetone for 15 min and then mechanically brushed using a *Kärcher WB 120* disk-brush for 5 min at 60 rpm with light contact pressure in an aqueous detergent solution (*Mucasol*™, 1 vol% in deionized water). They were successively ultrasonicated in a new detergent solution (15 min), deionized water (3 × 10 min) and isopropanol (15 min). This was followed up by a 30 min ultraviolet/ozone treatment (*UVO-Cleaner*®, *Jetlight Company Inc.*).

The substrates are then coated with PEDOT:PSS as hole-transporting layer prepared by spin-coating a colloidal suspension of PEDOT:PSS in water with a solid content of 1.3–1.7 % (PEDOT:PSS 1:6 *w*:*w*, *Heraeus*). The suspension was ultrasonicated at room temperature for 30 min and filtered through a 0.45 µm hydrophilic polyvinylidene fluoride syringe filter (*Kinesis group*) prior to spin-coating (*POLOS*™, *SPS-Europe spin-coater*) in a lamellar flowbox (*WIBO barrier*® *BAKVO 180/91*, *Weisstechnik*). The substrates were covered with the suspension and spin-coated at 2500 rpm with 4000 rpm s^{-1} acceleration for 30 s using a static dispense method. The substrates were then thermally annealed on a precision heating stage (*Harry Gestigkeit GmbH*) for 30 min at 240 °C. Next, the merocyanine donor layers were spin-coated under inert conditions (*M.Braun Inertgas Systeme GmbH*, *UNIlab Pro*, $c(O_2) < 1$ ppm, $c(H_2O) < 1$ ppm) according to the standard spin-coating procedure (*vide supra*).

The substrates were then transferred to an *OPTIvap-XL* (*CreaPhys GmbH*) thermal evaporation system equipped with 6 MHz sensing crystals (*umicore*). All interlayer and active layer materials were deposited at a pressure of < 2 × 10^{-6} mbar using a shadow mask at deposition rates of 0.1, 0.2, and 0.1 Å s^{-1} for bathocuproine (BCP, 99.99 %, trace metal basis, *Sigma Aldrich*), fullerene $\mathbf{C_{60}}$ (2x sublimed, 99.9 %, *CreaPhys GmbH*), and 8-quinolinolato lithium (Liq, 1x sublimed, > 99 %, *Ossila*), respectively. The substrate holder was rotated at 10 rpm during deposition to ensure more homogeneous layer deposition. The Al (99.99 %, *MaTeck*) top electrode was evaporated at rates of 0.1 to 3.0 Å s^{-1} at a pressure of < 5 × 10^{-6} mbar using a shadow mask, resulting in OPDs with 7.1 mm^2 active device area. Resultingly, device stacks of glass/ITO/PEDOT:PSS/merocyanine/20 nm $\mathbf{C_{60}}$/5 nm BCP/1 nm Liq/100 nm Al were fabricated. The device fabrication procedures are closely adapted from previous OPDs with this class of merocyanines without any further device optimization.[S40]

External quantum efficiency (*EQE*) measurements of the OPDs were performed at 0 V sample bias under inert conditions using a custom sample holder, a 300 W Xe ozone-free lamp (*6258*, *Oriel OPS-A500* Power Supply, *Newport*) as light source and an *Oriel 70710* (Gain = 10^5) pre-amplifier with a lock-in amplifier (*70104*, *Merlin*) for signal detection. Monochromic light was generated using an *Oriel Cornerstone*™ (*260 ¼ m*, *74125*) monochromator with a chopping frequency of 180 Hz and the measurement referenced to a calibrated Si detector (*70356_70316NS*, Gain = 10^5). From the measured *EQE*, the *R* was calculated as $R = EQE \times \lambda \times e \times h^{-1} \times c_{\text{light}}{}^{-1}$, where *e* represents the elementary charge, *h* the Planck constant, and c_{light} the speed of light in vacuum.[S41]

3 Calculations of Molecular Properties

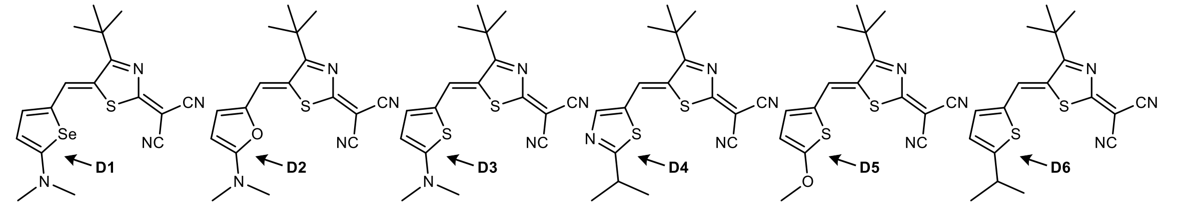

Figure S2. Chemical structures of merocyanine dyes used for (TD-)DFT calculations at a CAM-B3LYP/6-31G(d) level of theory bearing the **A1** acceptor unit and different donor units **D1-D6** with only methyl (-Me) substituents for reduced computational effort to estimate the shift in absorption upon changing the donor moiety.

Table S3. Calculation results of (TD-)DFT calculations at a CAM-B3LYP/6-31G(d) level of theory of merocyanine dyes shown in Figure S2 bearing the **A1** acceptor unit and different donor units **D1-D6** with methyl (Me) substituents for reduced computational effort to estimate the shift in absorption upon changing the donor moiety. Shifts in absorption are calculated relative to the original and previously investigated thiophene-containing **D1** for better comparability.

**Donor**	**HOMO** [eV]	**LUMO** [eV]	$\mu_{g;\,DFT}$ [D]	$\mu_{eg;\,TD\text{-}DFT}$ [D]	$\lambda_{TD\text{-}DFT}$ [nm]	$\tilde{\nu}_{TD\text{-}DFT}$ [cm^{-1}]	$\Delta\tilde{\nu}_{vs.\,D3}$ [cm^{-1}]	**Experimental shift of $\tilde{\nu}_{00}$ in solution vs. original donor D3** [cm^{-1}]
**D1**	−6.43	−1.83	14.9	9.7	452	22 146	−279	−175
**D2**	−6.41	−1.82	13.4	9.0	453	22 086	−339	−125
**D3**	−6.48	−1.84	14.6	9.6	446	22 425	0	0
**D4**	−6.75	−1.96	12.3	9.3	426	23 459	+1 034	+1 150
**D5**	−6.94	−2.04	15.3	9.1	413	24 213	+ 1 788	+1 850
**D6**	−7.13	−2.12	13.9	8.8	405	24 699	+ 2 274	+2 700

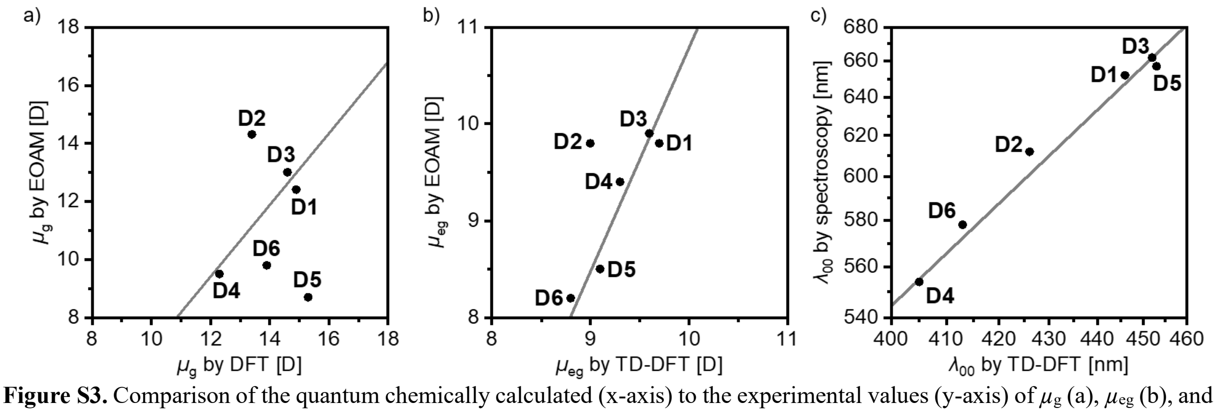

Figure S3. Comparison of the quantum chemically calculated (x-axis) to the experimental values (y-axis) of μ_g (a), μ_{eg} (b), and λ_{00} (c). The experimental data are taken for chromophores **D1(Hex)-A1**, **D2(Pyrl)-A1**, **D3(Hex)-A1**, **D4(Hex)-A1**, **D5(OMe)-A1**, and **D6(Hex)-A1**, as determined by EOAM or UV-Vis-NIR absorption spectroscopy. The calculated data are taken from Table S3 for the structures shown in Figure S2. The grey lines show an arbitrary linear trend as a guide to the eye.

4 Molecular & Pristine Solid-State Properties

Melting temperatures:

Table S4. Melting temperatures T_{melt} (uncorrected) of investigated merocyanine dyes determined using a digital automatic melting point apparatus.

Dye	T_{melt} [°C]
**D1(Pyrl)-A1**	260
**D3(Pyrl)-A1**	270
**D4(Pyrl)-A1**	290
**D5(OMe)-A1**	215
**D6(cPen)-A1**	215
**D1(Bu)-A2**	160
**D3(Bu)-A2**	165
**D4(Bu)-A2**	190
**D1(Hex)-A1**	155
**D3(Hex)-A1**	155
**D4(Hex)-A1**	205
**D6(Hex)-A1**	155

Molecular absorption properties in solution:

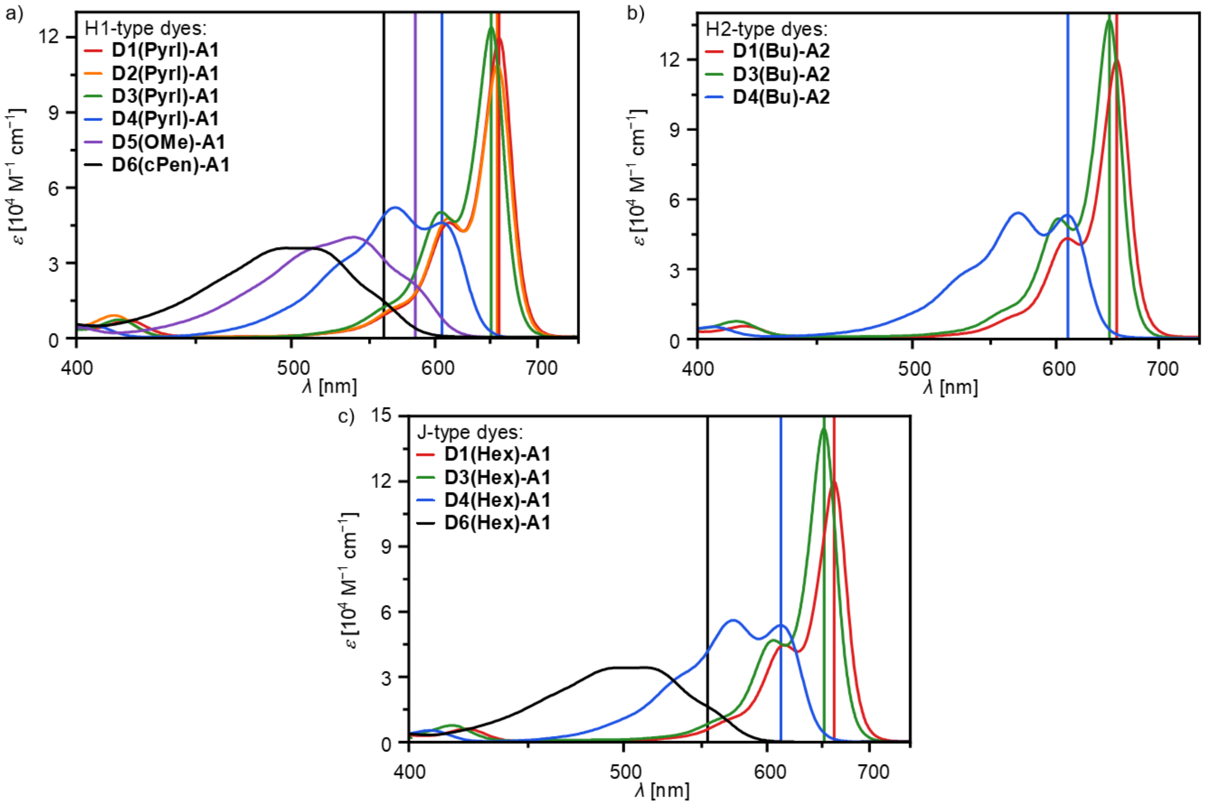

Figure S4. UV-Vis-NIR absorption spectra of merocyanine dyes in $CHCl_3$ solution at 10^{-5} M and 298 K sorted by their chemical structures / aggregate types, namely H1- (a), H2- (b), and J-type (c) aggregating dyes. The colored vertical lines mark the respective A_{00} transitions of the individual merocyanine dyes bearing a **D1** (red), **D2** (orange), **D3** (green), **D4** (blue), **D5** (violet), or **D6** (black) donor moiety.

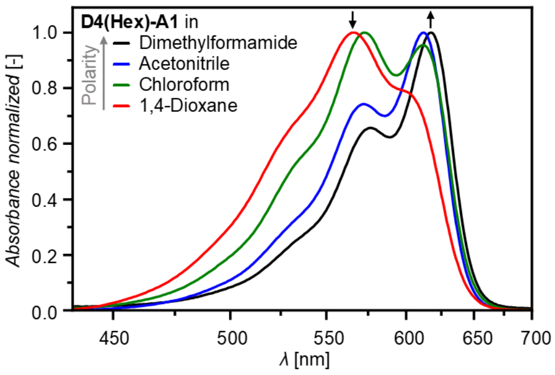

Figure S5. Normalized UV-Vis-NIR absorption spectra of **D4(Hex)-A1** in solvents of varying polarity; the black arrows indicate the change of the band shape upon increase of solvent polarity.

Table S5. Solvent-dependency of the absorption band shape of **D4(Hex)-A1** in solvents with increasing relative permittivity ε_r.[S42]

Solvent	ε_r [-]	λ_{00} [nm]	$\tilde{\nu}_{00}$ [cm^{-1}]	A_{00}/A_{01} [-]
1,4-Dioxane [a)]	2	605	16 525	0.7
$CHCl_3$	5	612	16 350	0.9
Acetonitrile	36	612	16 350	1.3
N,N-Dimethylformamide	37	617	16 200	1.5

[a)] The values in 1,4 dioxane were estimated from the absorption spectrum due to the broadened absorption band shape.

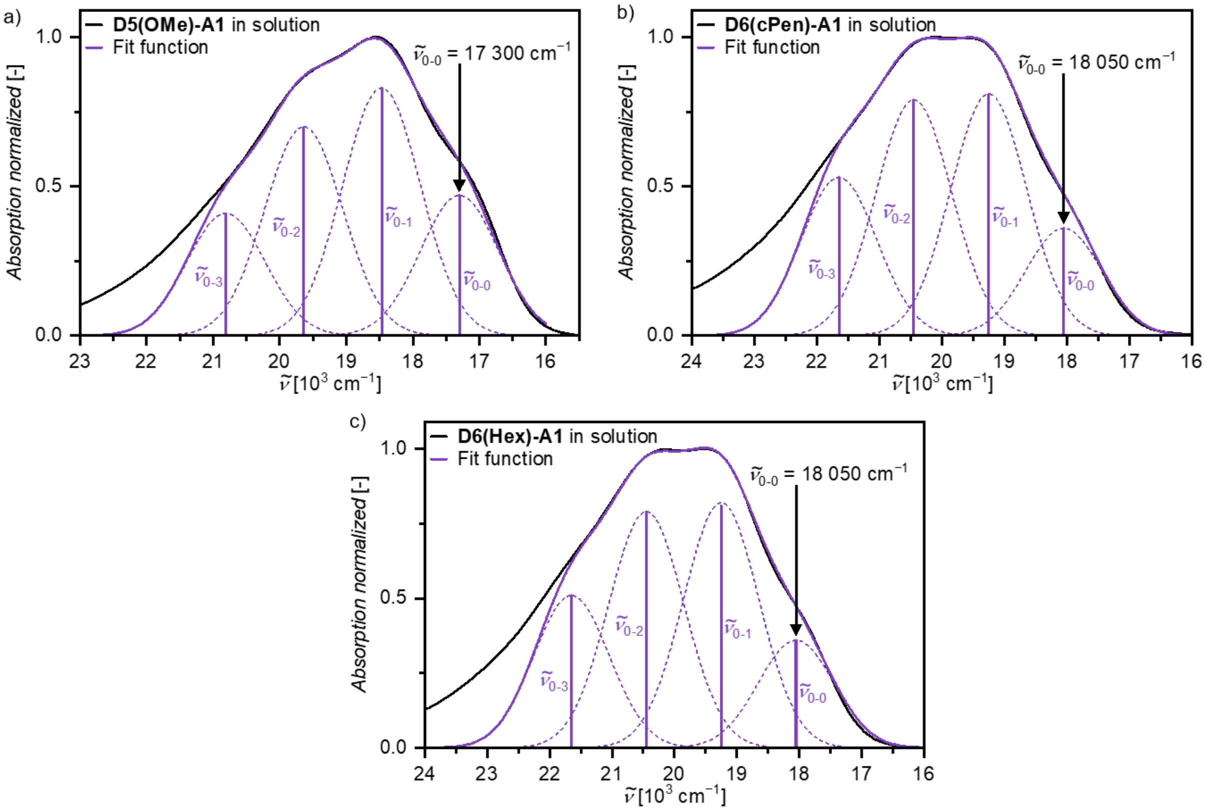

Figure S6. Franck-Condon band fitting analysis of the $S_0 \rightarrow S_1$ absorption bands of **D5(OMe)-A1** (a), **D6(cPen)-A1** (b), and **D6(Hex)-A1** (c) in 10^{-5} M $CHCl_3$ solutions at 298 K. The experimental spectra are shown in black and the fitted data in violet. The respective fit parameters used for the Gaussian fit functions for **D5(OMe)-A1**, **D6(cPen)-A1**, and **D6(Hex)-A1** were as follows: 0-0 transition – 17 300, 18 050, and 18 050 cm^{-1}; *fwhm* – 830, 860, and 855 cm^{-1}; band distance – 1 170, 1 200, and 1 200 cm^{-1}; relative peak intensities (0-0:0-1:0-2:0-3) – 0.47:0.83:0.70:0.41, 0.36:0.81:0.79:0.53, and 0.36:0.82:0.79:0.52. The violet vertical lines mark the transition peaks scaled according to the determined relative peak intensities, the violet dashed lines show the individual transition Gaussian fit functions, and the violet solid lines represent the sum of all four respective fit functions.

Electro-optical absorption measurements (EOAM):

Table S6. Optical and electro-optical properties of merocyanine dyes as investigated by UV-Vis-NIR absorption spectroscopy and EOAM in $CHCl_3$ at 298 K.

Dye	λ_{max} [a] [nm]	λ_{00} [b] [nm]	***fwhm*** $fwhm_{opt}$ [c] [nm, cm⁻¹]	$fwhm_{00}$ [d] [nm, cm⁻¹]	μ_{eg} [D]	μ_{eg}^2 [D²]	μ_g [D]	μ_g^2 [D²]	$\Delta\mu$ [D]	c^2 [-]	$\mu_{eg; TD-DFT}$ [e] [D]	$\mu_{g; DFT}$ [e] [D]
**D1(Hex)-A1**	662	662	35, 810	32, 735	9.8	96	12.4 ± 0.4	153.8 ± 9.9	−0.1 ± 0.3	0.50 ± 0.01	9.7	14.9
**D2(Pyrl)-A1**	657	657	36, 840	32, 745	9.8	96	14.3 ± 0.4	204.5 ± 11.4	−0.9 ± 0.3	0.52 ± 0.01	9.0	13.4
**D3(Hex)-A1**	652	652	35, 830	30, 710	9.9	98	13.0 ± 0.3	169.0 ± 7.8	0.3 ± 0.3	0.49 ± 0.01	9.6	14.6
**D4(Hex)-A1**	573	612	101, 3 010	42, 1 125	9.4	88	9.5 ± 0.5	90.3 ± 9.5	4.4 ± 1.0	0.38 ± 0.02	9.3	12.3
**D5(OMe)-A1**	540	578	107, 3 825	47, 1 395	8.5	72	8.7 ± 0.1	75.7 ± 1.7	5.3 ± 0.5	0.35 ± 0.01	9.1	15.3
**D6(Hex)-A1**	513	554	99, 3 975	43, 1 405	8.2	67	9.8 ± 0.1	96.0 ± 2.0	5.7 ± 0.3	0.34 ± 0.01	8.8	13.9

[a] Absorption maximum of the UV-Vis-NIR absorption spectrum; [a] Position of the A_{00} transition; [c] The $fwhm_{opt}$ value of the UV-Vis-NIR absorption spectrum in solution is determined as the full width of the complete unsymmetric absorption band; [d] The $fwhm_{00}$ value of UV-Vis-NIR absorption spectrum is determined as the full width of the symmetric A_{00} transition; [e] Values determined from similar compounds with methyl donor substituents by quantum chemical calculations (see Figure S2).

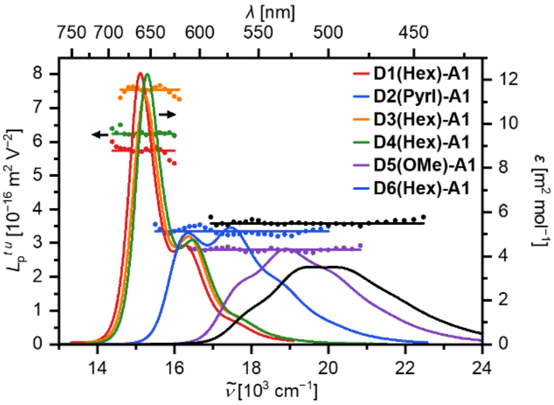

Figure S7. UV-Vis-NIR absorption spectra (solid lines) along with the linear combination L_p^{tu} (symbols), which is proportional to μ_g^2 (horizontal lines), determined by EOAM in $CHCl_3$ at 298 K for merocyanine dyes **D1(Hex)-A1** (red), **D2(Pyrl)-A1** (orange), **D3(Hex)-A1** (green), **D4(Hex)-A1** (blue), **D5(OMe)-A1** (violet), and **D6(Hex)-A1** (black).

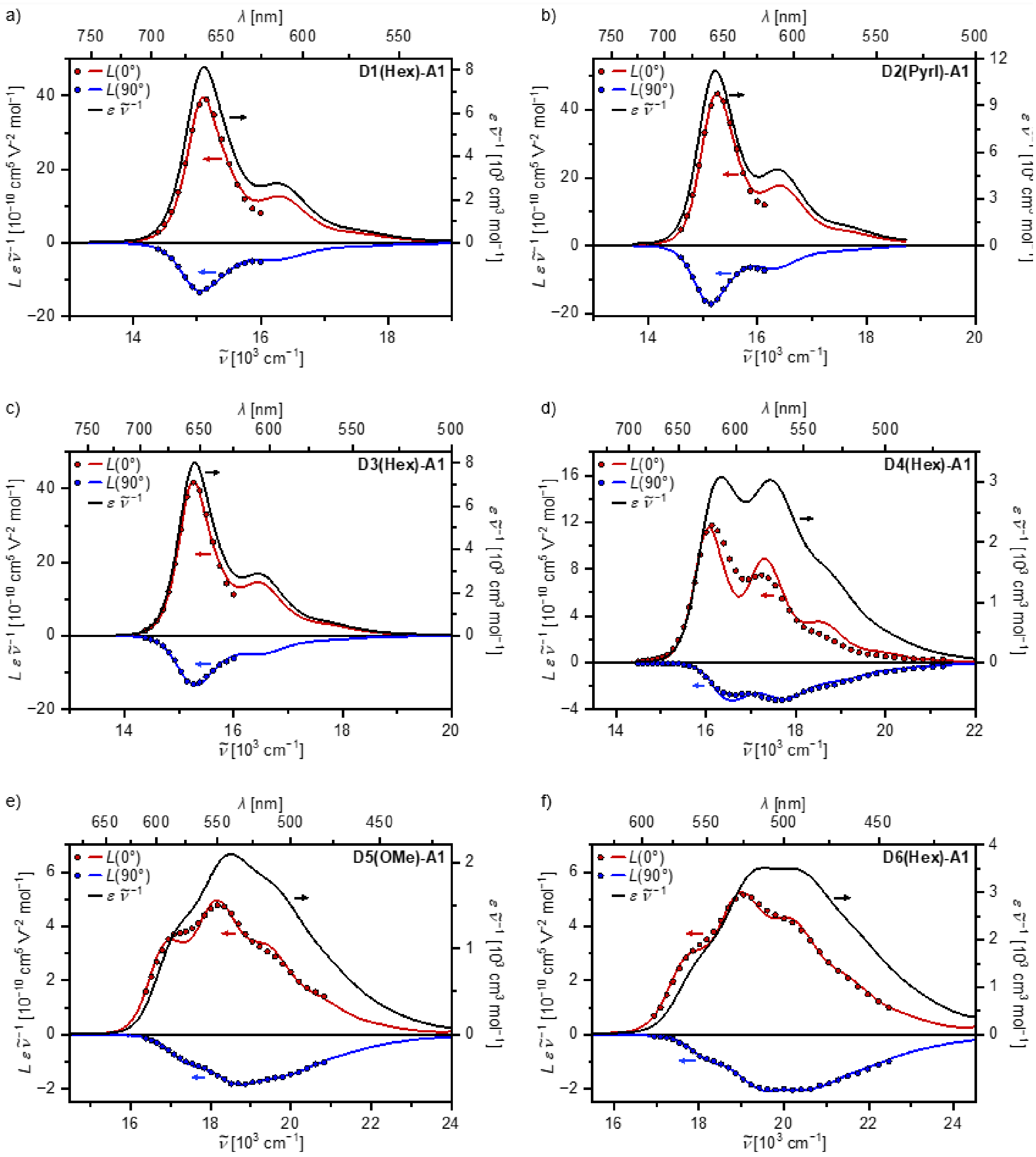

Figure S8. EOAM and UV-Vis-NIR absorption spectra of merocyanine dyes **D1(Hex)-A1** (a), **D2(Pyrl)-A1** (b), **D3(Hex)-A1** (c), **D4(Hex)-A1** (d), **D5(OMe)-A1** (e), and **D6(Hex)-A1** (f) in $CHCl_3$ at 298 K. The EOAM signal (circles) is shown for light parallely (0°; red) and perpendicularly (90°; blue) polarized to the direction of the externally applied electric field **E** and includes the corresponding multilinear regressions (solid red and blue lines). The UV-Vis-NIR absorption spectra are shown as black solid lines.

Differential pulse voltammetry (DPV):

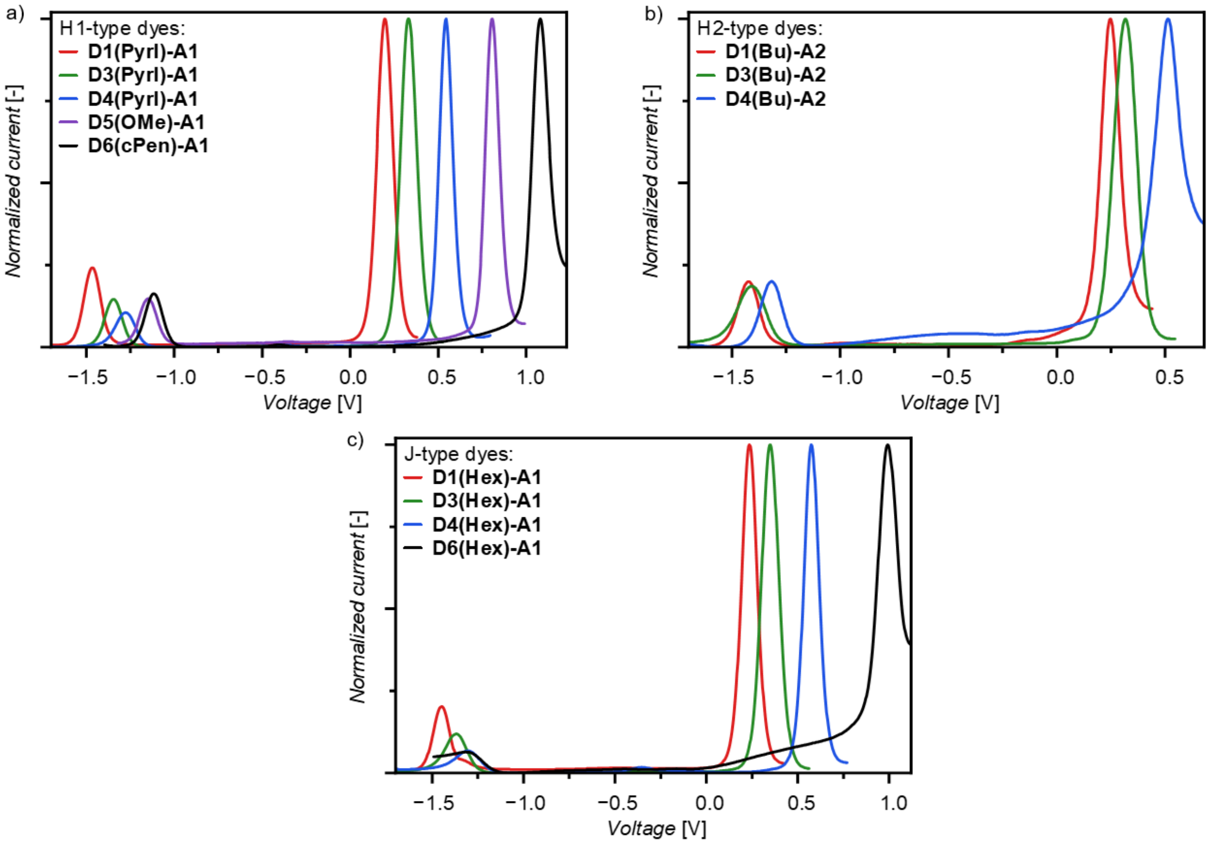

Figure S9. DPV data of merocyanine dyes determined in 2×10^{-5} M CH_2Cl_2 solutions under ambient conditions sorted by their chemical structures / aggregate types, namely H1- (a), H2- (b), and J-type (c) dyes. All data were calibrated to the ferrocene/ferrocenium (Fc/Fc^+) couple of −5.15 eV with a scan rate of 100 mV s^{-1} (voltage steps of 0.004 V) and tetrabutylammonium hexafluorophosphate ($(n\text{-}Bu)_4NPF_6$; 0.1 M) as an electrolyte.

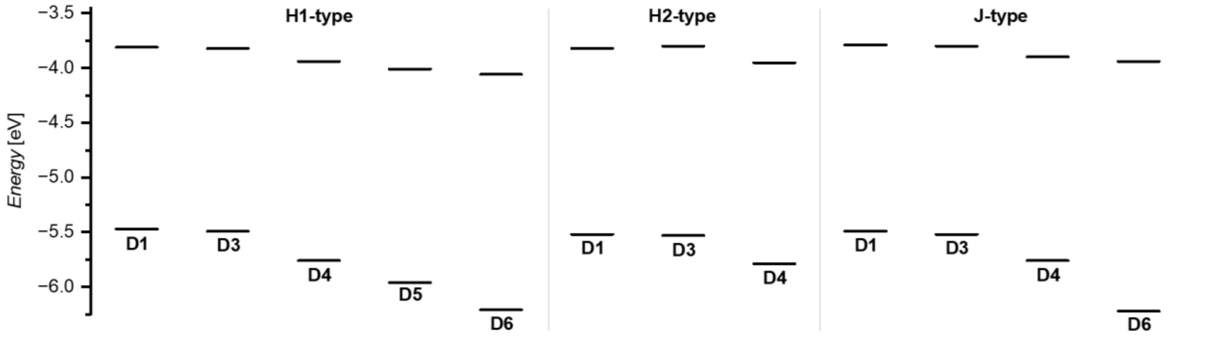

Figure S10. Frontier orbital (HOMO / LUMO) levels of merocyanine dyes as calculated from DPV data in Figure S9 and Table S7 calibrated to the ferrocene/ferrocenium (Fc/Fc^+) couple of −5.15 eV.

Table S7. Oxidation and reduction potentials (E_{ox}, E_{red}),[a] frontier orbital levels (HOMO, LUMO),[b] and the electrochemical bandgap (E_{gap})[c] of merocyanine dyes determined at 2×10^{-5} M in CH_2Cl_2 under ambient conditions at 298 K. The optical bandgap (E_{opt}), determined as exemplarily shown in Figure S11 from the respective monomeric absorption spectra from Figure S4, and the energy difference compared to E_{gap} are given for comparison.

	Dye	E_{ox} [V]	E_{red} [V]	HOMO [eV]	LUMO [eV]	E_{gap} [eV]	E_{opt} [eV]	$E_{opt} - E_{gap}$ [eV]
H1-type	**D1(Pyrl)-A1**	0.32	−1.34	−5.47	−3.81	1.66	1.81	0.1
	**D3(Pyrl)-A1**	0.34	−1.33	−5.49	−3.82	1.67	1.83	0.2
	**D4(Pyrl)-A1**	0.61	−1.21	−5.76	−3.94	1.82	1.93	0.1
	**D5(OMe)-A1**	0.81	−1.14	−5.96	−4.01	1.94	2.01	0.1
	**D6(cPen)-A1**	1.06	−1.09	−6.21	−4.06	2.16	2.10	−0.1
H2-type	**D1(Bu)-A2**	0.37	−1.33	−5.52	−3.82	1.70	1.82	0.1
	**D3(Bu)-A2**	0.38	−1.35	−5.53	−3.80	1.73	1.84	0.1
	**D4(Bu)-A2**	0.64	−1.20	−5.79	−3.95	1.84	1.93	0.1
J-type	**D1(Hex)-A1**	0.34	−1.36	−5.49	−3.79	1.70	1.80	0.1
	**D3(Hex)-A1**	0.37	−1.35	−5.52	−3.80	1.72	1.83	0.1
	**D4(Hex)-A1**	0.61	−1.25	−5.76	−3.90	1.86	1.92	0.1
	**D6(Hex)-A1**	1.07	−1.21	−6.22	−3.94	2.28	2.11	−0.2

[a] E_{ox} and E_{red} determined by differential pulse voltammetry (DPV) with a scan rate of 100 mV s^{-1} (voltage steps of 0.004 V) and tetrabutylammonium hexafluorophosphate ($(n\text{-Bu})_4NPF_6$; 0.1 M) as an electrolyte; it is noted that all reduction processes are irreversible as determined by corresponding cyclic voltammograms using identical measurement parameters. [b] HOMO and LUMO values were calculated by calibrating the data to the ferrocene/ferrocenium (Fc/Fc^+) couple of −5.15 eV. [c] E_{gap} was calculated as the energetic difference between the electrochemically determined values for the HOMO and LUMO.

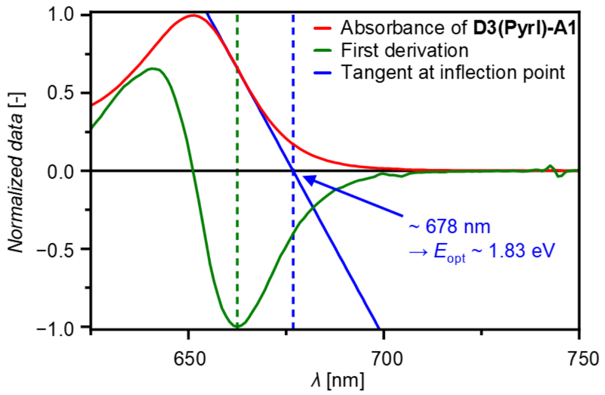

Figure S11. Exemplary depiction of the determination of the optical bandgap (E_{opt}) from the monomeric UV-Vis-NIR absorption spectrum of **D3(Pyrl)-A1** (solid red) measured in $CHCl_3$ (see Figure S4 for all monomeric spectra). The E_{opt} was determined by first calculating the first derivative of the absorption spectrum (solid green) and then forming the tangent of the absorption spectrum (solid blue) at the wavelength of the lowest-energy minimum of the first derivative (= lowest-energy inflection point of the absorption spectrum; vertical dashed green). The bandgap wavelength is then taken as the intersection of the tangent with the x-axis (vertical dashed blue). E_{opt} for all other dyes was determined analogously and is listed in Table S7 for comparison with the electrochemically determined E_{gap}.

Pristine Thin Film Absorption Spectra:

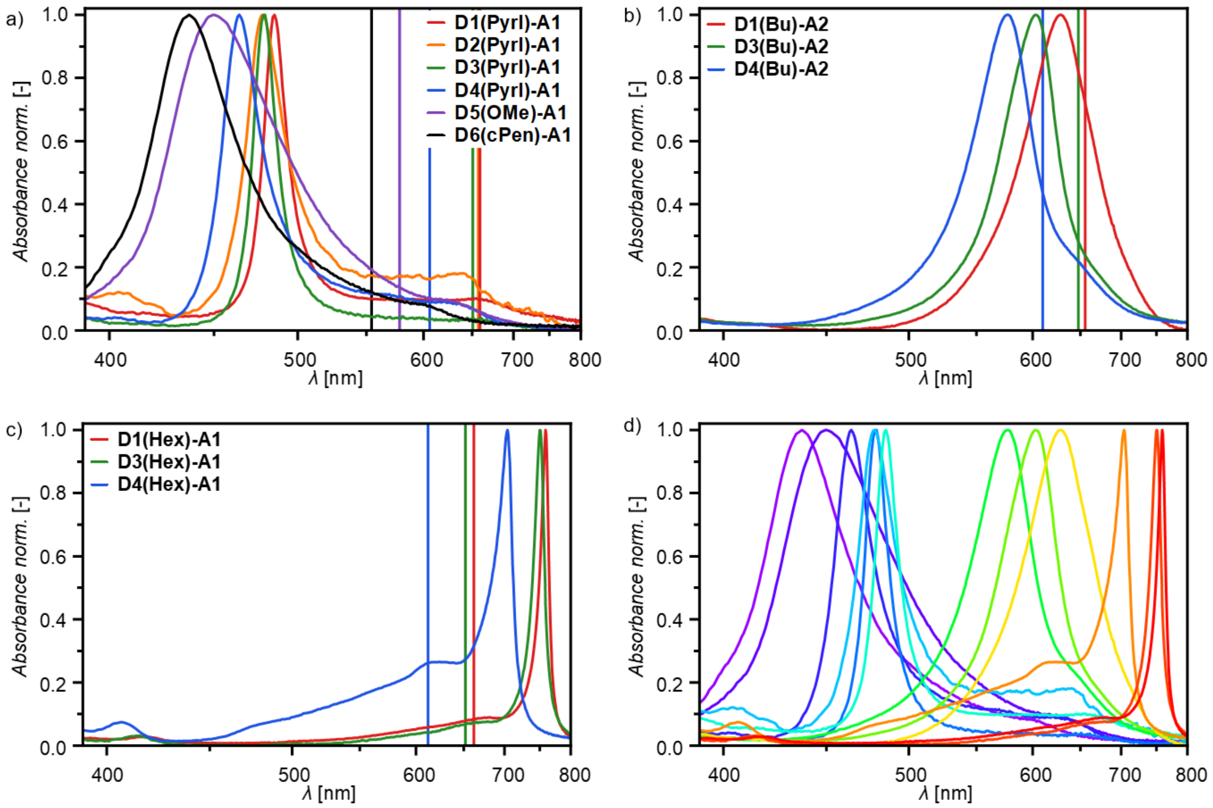

Figure S12. Normalized absorption spectra of annealed pristine thin films of merocyanine dyes sorted by their aggregate types, namely H1- (a), H2- (b), and J-type aggregates (c), alongside a spectral overview of the aggregate absorption spectra of all investigated merocyanine dyes (d). The colored vertical lines in a)-c) mark the corresponding monomeric A_{00} transitions in $CHCl_3$ solution.

D6(Hex)-A1 Thin Film Absorption Spectra:

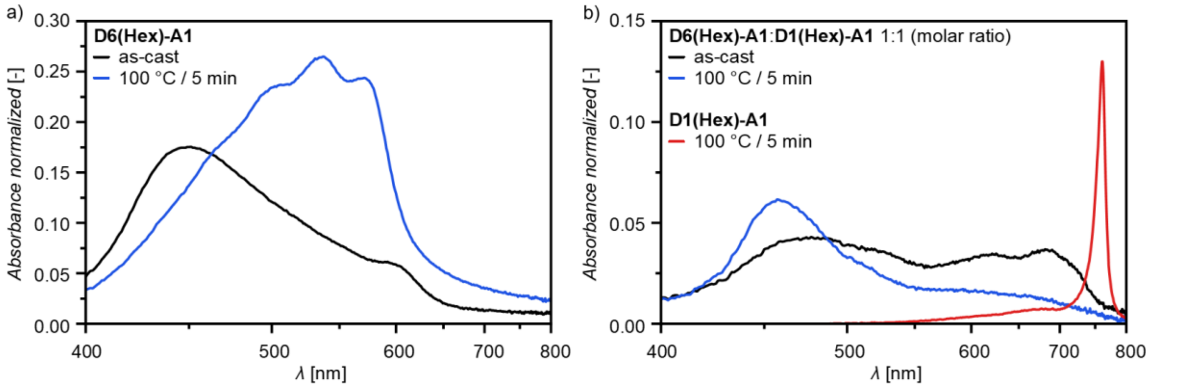

Figure S13. Thin film UV-Vis-NIR absorption spectra of **D6(Hex)-A1** in pristine films (a) as well as in films mixed with **D1(Hex)-A1** at a 1:1 molar ratio (b). Both films are shown as-cast (black) and after thermal annealing at 100 °C for 5 min (blue). In b) a scaled spectrum of the pristine J-aggregate of **D1(Hex)-A1** (red) is shown as reference.

5 Mixed Thin-Film Spectroscopy

Possible Expectations for Mixed Thin Film Absorption Spectra:

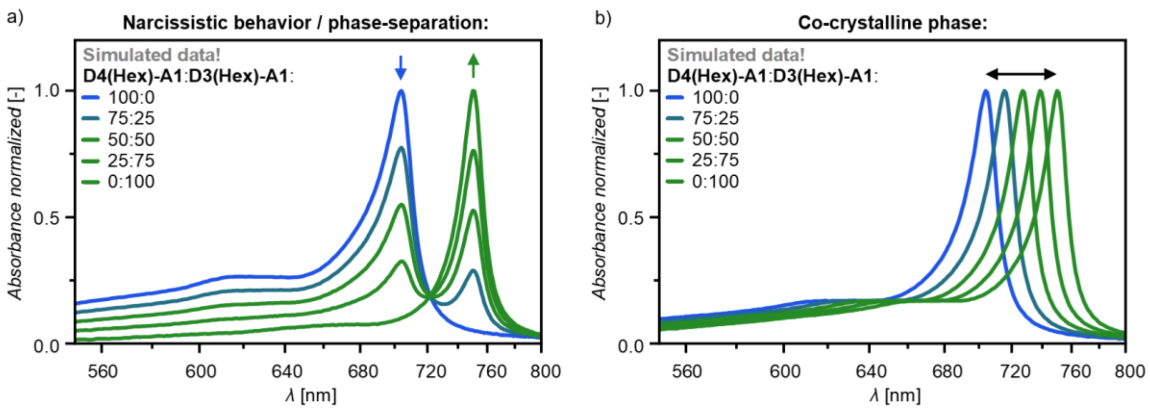

Figure S14. Simulated data of two possible scenarios for expectations of mixed and annealed thin films of **D4(Hex)-A1**:**D3(Hex)-A1** showing either narcissistic behavior / phase-separation (a) or co-crystalline phase formation (b). The data were generated by a relative superposition and normalization of the J-aggregate absorption bands of pristine annealed thin films of the two compounds.

Mixed Thin Film Absorption Data:

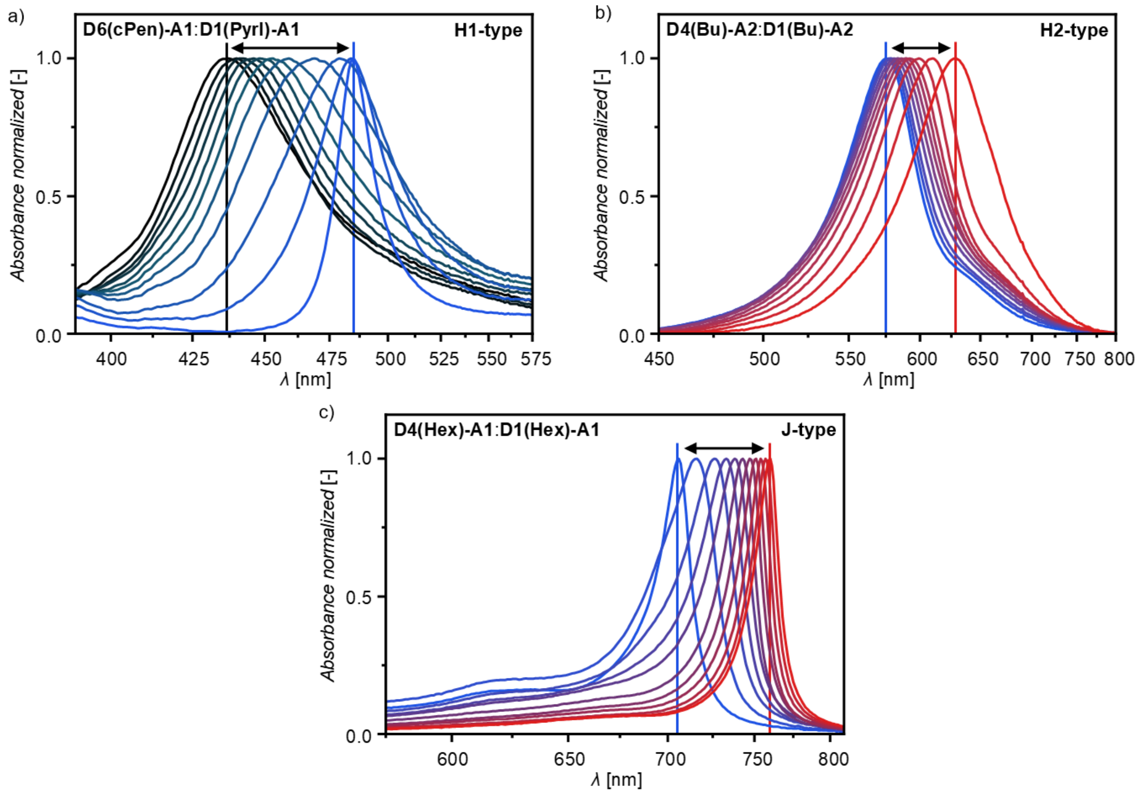

Figure S15. Normalized absorption spectra of mixed annealed thin films on quartz substrates of mixtures of dyes with the spectrally furthest apart pristine aggregate absorption bands within each type of aggregate series, namely **D6(cPen)-A1**:**D1(Pyrl)-A1** (a; H1-type), **D4(Bu)-A2**:**D1(Bu)-A2** (b; H2-type), and **D4(Hex)-A1**:**D1(Hex)-A1** (c; J-type). The mixture ratios X:Y are varied in steps of 10 % (molar ratio).

Table S8. Optical properties of annealed thin films spin-coated from 4×10^{-3} M $CHCl_3$ solutions at different molar mixing ratios onto quartz substrates of dye mixtures **D4(Pyrl)-A1**:**D1(Pyrl)-A1** (H1-type; see Figure 2a for spectra) as well as **D6(cPen)-A1**:**D1(Pyrl)-A1** (H1-type), **D4(Bu)-A2**:**D1(Bu)-A2** (H2-type), and **D4(Hex)-A1**:**D1(Hex)-A1** (J-type; see Figure S15 for spectra). The annealing temperatures for the dye mixtures were 130 °C for 5 min, except for the J-type mixture, which was annealed using a gradient from 170 °C (0:10) to 130 °C (10:0) for 5 min in steps of 4 °C due to the significant difference in T_{melt} of the two components (Table S4).

**Mixture ratio**	**H1-type – D4(Pyrl)-A1:D1(Pyrl)-A1**			**H1-type – D6(cPen)-A1:D1(Pyrl)-A1**		
	λ_{opt} [nm, cm^{-1}]	OD_{max} [-]	$fwhm_{opt}$ [nm, cm^{-1}]	λ_{opt} [nm, cm^{-1}]	OD_{max} [-]	$fwhm_{opt}$ [nm, cm^{-1}]
10:0	464, 21 550	0.19	28, 1 275	437, 22 900	0.10	60, 3 025
9:1	466, 21 450	0.18	26, 1 175	440, 22 725	0.10	55, 2 800
8:2	468, 21 375	0.18	26, 1 175	442, 22 625	0.10	56, 2 775
7:3	470, 21 275	0.18	26, 1 150	446, 22 425	0.09	58, 2 875
6:4	472, 21 175	0.17	26, 1 150	449, 22 275	0.09	61, 2 950
5:5	475, 21 050	0.18	25, 1 100	453, 22 075	0.08	65, 3 075
4:6	477, 20 975	0.18	24, 1 050	459, 21 775	0.08	72, 3 275
3:7	478, 20 925	0.16	24, 1 050	469, 21 325	0.08	73, 3 250
2:8	481, 20 800	0.17	22, 950	479, 20 875	0.09	58, 2 525
1:9	483, 20 700	0.18	20, 850	484, 20 650	0.12	38, 1 625
0:10	485, 20 625	0.19	20, 825	485, 20 625	0.19	20, 825
**Mixture ratio**	**H2-type – D4(Bu)-A2:D1(Bu)-A2**			**J-type – D4(Hex)-A1:D1(Hex)-A1**		
	λ_{opt} [nm, cm^{-1}]	OD_{max} [-]	$fwhm_{opt}$ [nm, cm^{-1}]	λ_{opt} [nm, cm^{-1}]	OD_{max} [-]	$fwhm_{opt}$ [nm, cm^{-1}]
10:0	575, 17 400	0.33	61, 1 850	706, 14 150	0.31	23, 475
9:1	577, 17 325	0.32	63, 1 925	716, 13 975	0.24	42, 828
8:2	579, 17 275	0.31	66, 2 000	726, 13 775	0.27	39, 750
7:3	581, 17 200	0.29	70, 2 100	733, 13 650	0.28	34, 650
6:4	584, 17 125	0.27	73, 2 175	738, 13 550	0.28	31, 575
5:5	586, 17 050	0.26	75, 2 200	743, 13 450	0.36	26, 474
4:6	590, 16 975	0.26	75, 2 200	747, 13 375	0.42	24, 425
3:7	592, 16 900	0.26	74, 2 150	751, 13 325	0.45	22, 375
2:8	599, 16 700	0.26	73, 2 050	754, 13 275	0.48	20, 350
1:9	610, 16 400	0.26	73, 2 000	757, 13 200	0.49	22, 375
0:10	629, 15 900	0.25	89, 2 275	760, 13 150	0.44	20, 350

Solution-Sheared Mixed Thin Films:

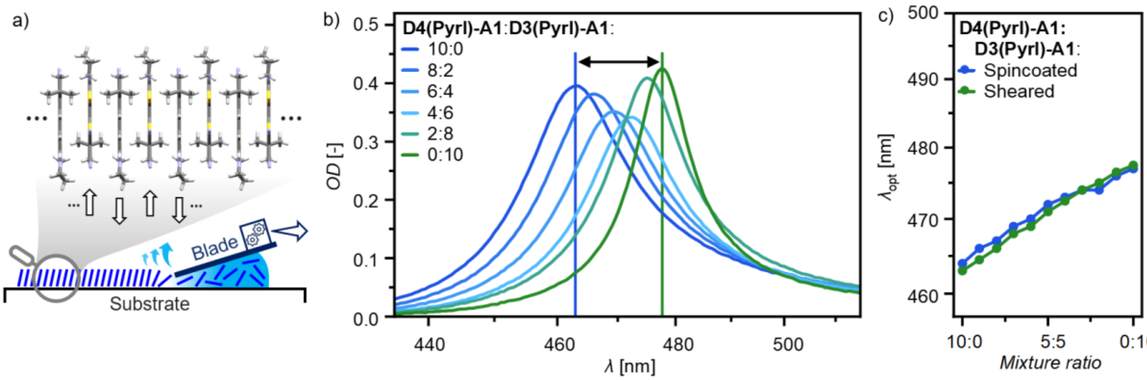

Figure S16. Solution-sheared mixed H1-type thin films of **D4(Pyrl)-A1**:**D3(Pyrl)-A1** including a schematic depiction of the shearing process (a), absorption spectra of linearly polarized light perpendicular (90°) to the shearing direction of mixed thin films (b), and the H-band absorption maximum plotted over the mixture ratio of both spin-coated (blue) and solution-sheared (green) mixed thin films (c). The mixture ratio given represents a molar mixing ratio. The vertical lines in b) mark the respective absorption maxima λ_{00} of thin films of pristine **D4(Pyrl)-A1** (blue) and **D3(Pyrl)-A1** (green), respectively. Figure a) was adapted with permission from © 2024 The Royal Society of Chemistry from ref. S40.

Table S9. Optical properties of solution-sheared H1-type mixed thin films at different mixing ratios in steps of 10 % of **D4(Pyrl)-A1**:**D3(Pyrl)-A1**, including the maximum H-band absorption of light linearly polarized perpendicular (90°) to the shearing direction *OD*(90°), and the optical dichroic ratio DR_{opt}, which represents the fraction of the absorption of linearly polarized light perpendicular (90°, maximum) to that polarized parallel (0°, minimum) to the shearing direction.

**D3(Pyrl)-A1: D4(Pyrl)-A1**	λ_{opt} [nm]	***OD*(90°)** [-]	$fwhm_{opt}$ [nm]	$fwhm_{opt}$ [cm^{-1}]	DR_{opt} [-]
10:0	478	0.43	14	600	36
9:1	477	0.41	15	675	28
8:2	475	0.42	19	825	20
7:3	474	0.38	20	900	17
6:4	473	0.35	22	950	13
5:5	471	0.39	22	975	17
4:6	469	0.36	23	1 050	13
3:7	468	0.35	23	1 050	12
2:8	466	0.39	23	1 050	14
1:9	465	0.36	23	1 075	11
0:10	463	0.40	23	1 075	14

6 Single-Crystal and Co-Crystal Structures

Single-Crystal Structures:

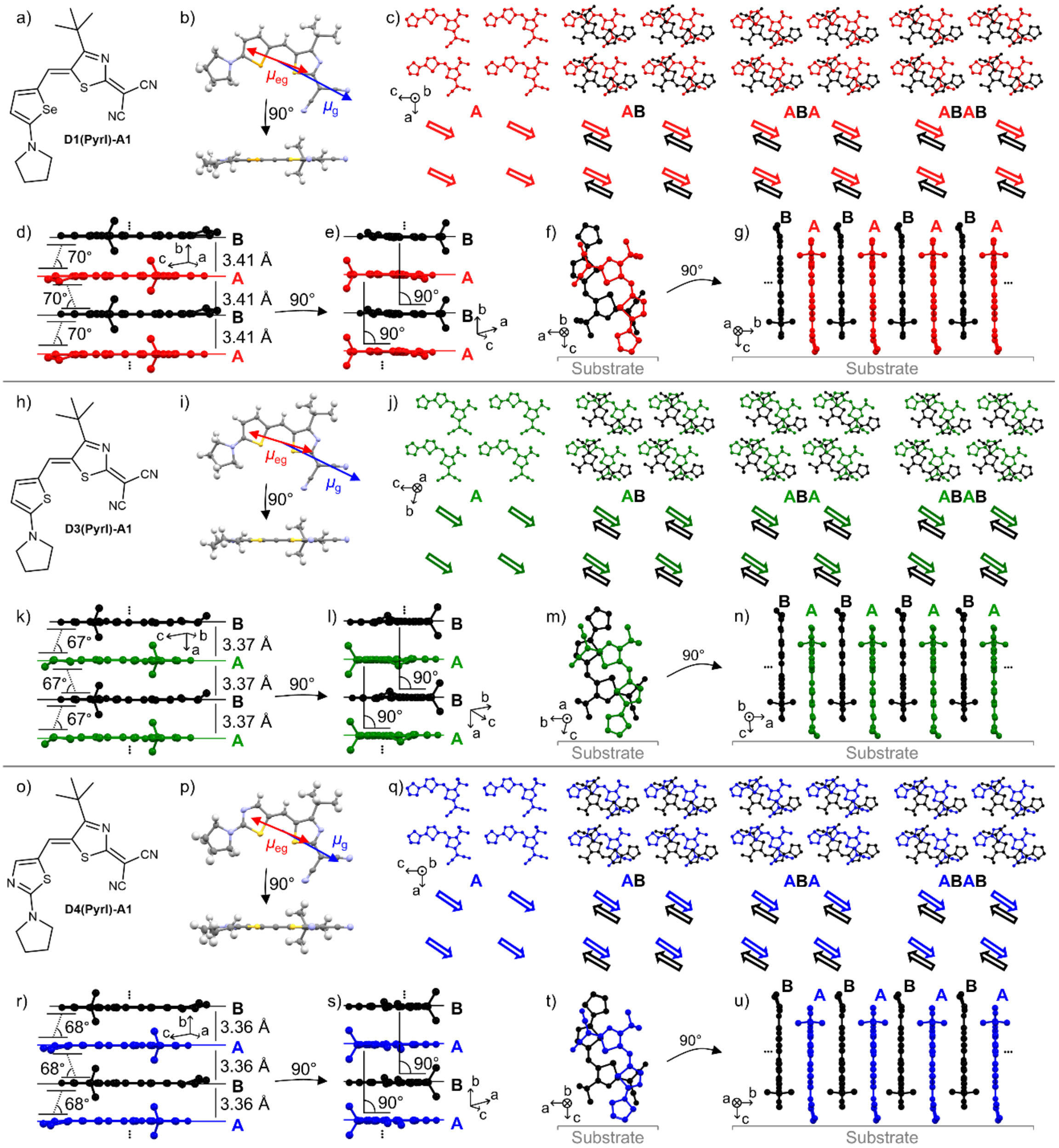

Figure S17. Single-crystal structures of H1-type coupled merocyanines **D1(Pyrl)-A1** (a-g), **D3(Pyrl)-A1** (h-n), and **D4(Pyrl)-A1** (o-u). a,h,o) Chemical structures of the dyes. b,i,p) Crystal structure with 50 % probability ellipsoids alongside the ground (blue) and transition (red) dipole moment μ_g and μ_{eg} orientation, respectively. c,j,q) Top view along the π-stacking direction on individual layers of four parallel π-stacks as well as a schematic orientation of the dipole moments μ_g (arrows). d,k,r and e,l,s) Side views of a tetramer π-stack of dyes including measured π-π-distances $d_{\pi\text{-}\pi}$ and slip angles Θ_{slip} between the donor-acceptor-bridge carbon atoms. f,m,t and g,n,u) Orientation of the molecules as determined by TF-XRD in respect to a substrate surface in annealed spin-coated thin films on quartz substrates with both a top view and side view of the π-stack, respectively. Hydrogen atoms (except in b, i and p) and solvent molecules were omitted for clarity.

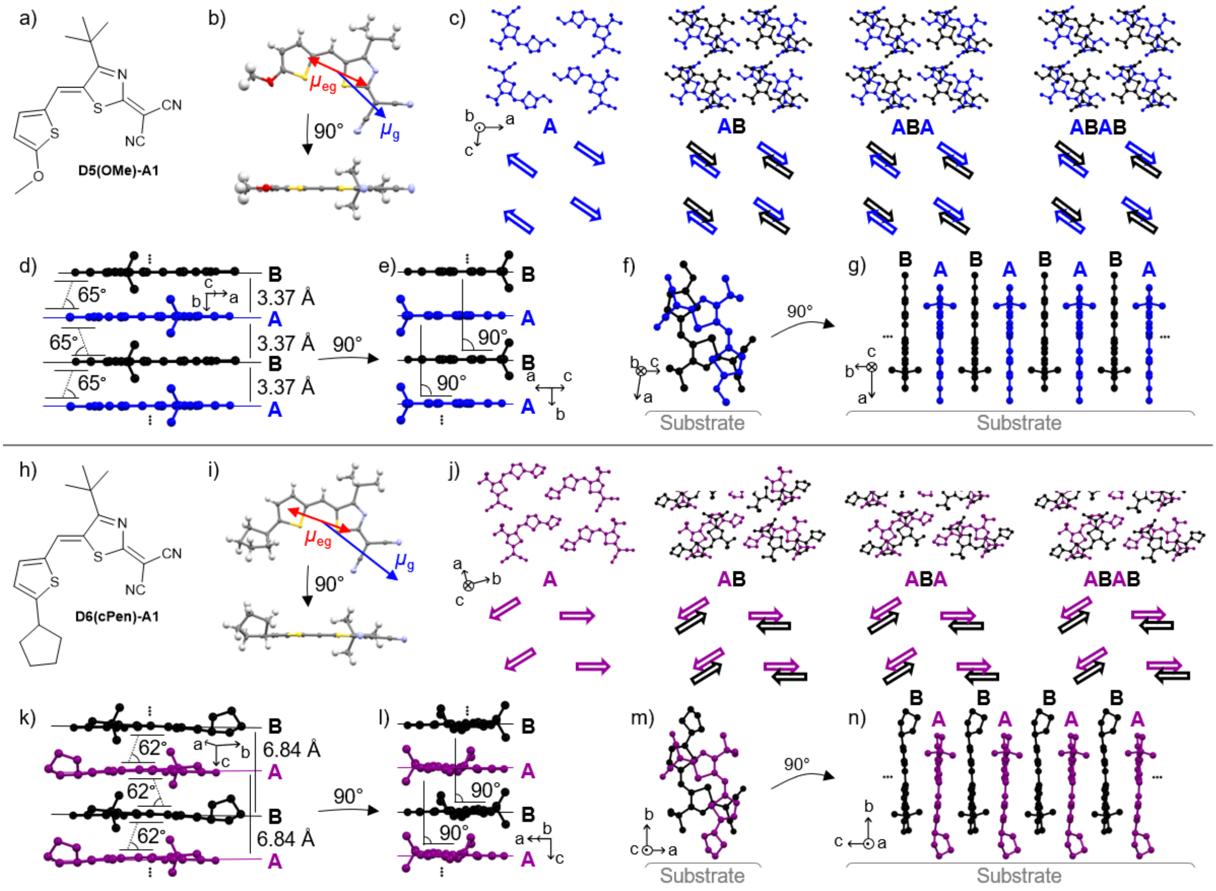

Figure S18. Single-crystal structures of H1-type coupled merocyanines **D5(OMe)-A1** (a-g) and **D6(cPen)-A1** (h-n). a,h) Chemical structures of the dyes. b,i) Crystal structure with 50 % probability ellipsoids alongside the ground (blue) and transition (red) dipole moment μ_g and μ_{eg} orientation, respectively. c,j) Top view along the π-stacking direction on individual layers of four parallel π-stacks as well as a schematic orientation of the dipole moments μ_g (arrows). d,k and e,l) Side views of a tetramer π-stack of dyes including measured π-π-distances $d_{\pi\text{-}\pi}$ and slip angles Θ_{slip} between the donor-acceptor-bridge carbon atoms. f,m and g,n) Orientation of the molecules as determined by TF-XRD in respect to a substrate surface in annealed spin-coated thin films on quartz substrates with both a top view and side view of the π-stack, respectively. Hydrogen atoms (except in b, i and p) and solvent molecules were omitted for clarity.

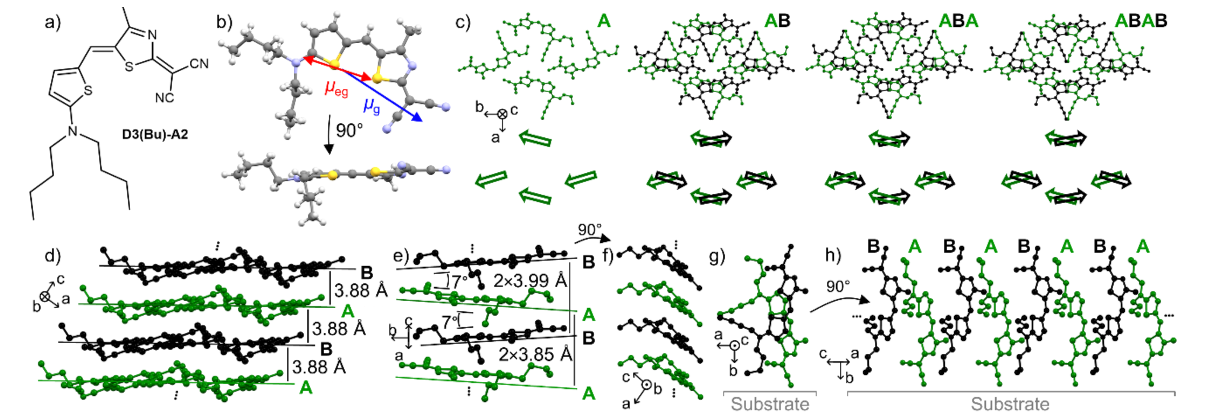

Figure S19. Single-crystal structure of H2-type coupled merocyanine **D3(Bu)-A2**. a) Chemical structure of the dye. b) Crystal structure with 50 % probability ellipsoids alongside the ground (blue) and transition (red) dipole moment μ_g and μ_{eg} orientation, respectively. c) Top view along the π-stacking direction on individual layers of four parallel π-stacks as well as a schematic orientation of the dipole moments μ_g (arrows). d) Side view onto the parallel ABAB π-stacks from subfigure c). e and f) Side views of a tetramer π-stack of dyes including measured π-π-distances $d_{\pi\text{-}\pi}$ and slip angles Θ_{slip} between the donor-acceptor-bridge carbon atoms. g and h) Orientation of the molecules as determined by TF-XRD in respect to a substrate surface in annealed spin-coated thin films on quartz substrates with both a top view and side view of the π-stack, respectively. Hydrogen atoms (except in b) and solvent molecules were omitted for clarity.

Tabular Crystal Data of Single- and Co-Crystals:

Table S10. Crystal data and structure refinement for **D1(Pyrl)-A1** single-crystal.

Empirical formula	$C_{20}H_{24}N_4OSSe$
Formula weight	447.45
Temperature	100(2) K
Wavelength	1.54178 Å
Crystal system	Monoclinic
Space group	$P2_1/m$
Unit cell dimensions	a = 9.7884(10) Å α = 90°<br>b = 6.8251(7) Å β = 91.169(7)°<br>c = 15.8995(17) Å γ = 90°
Volume	1061.97(19) Å^3
Z	2
Density (calculated)	1.399 g cm^{-3}
Absorption coefficient	3.443 mm^{-1}
F(000)	460
Crystal size	0.330 × 0.070 × 0.028 mm^3
Theta range for data collection	2.780 to 72.221°
Index ranges	$-12 \le h \le 12$, $-8 \le k \le 8$, $-19 \le l \le 19$
Reflections collected	20862
Independent reflections	2282 [R(int) = 0.0261]
Completeness to theta = 67.679°	100.0 %
Absorption correction	Semi-empirical from equivalents
Max. and min. transmission	0.7536 and 0.5227
Refinement method	Full-matrix least-squares on F^2
Data / restraints / parameters	2282 / 0 / 172
Goodness-of-fit on F^2	1.060
Final R indices [$I > 2\sigma(I)$]	R_1 = 0.0220, wR_2 = 0.0588
R indices (all data)	R_1 = 0.0223, wR_2 = 0.0590
Extinction coefficient	n/a
Largest diff. peak and hole	0.458 and –0.327 e × Å^{-3}

Table S11. Crystal data and structure refinement for **D4(Pyrl)-A1** single-crystal.

Empirical formula	$C_{19}H_{23}N_5OS_2$
Formula weight	401.54
Temperature	100(2) K
Wavelength	0.61992 Å
Crystal system	Monoclinic
Space group	$P2_1/m$
Unit cell dimensions	a = 9.743(4) Å α = 90°<br>b = 6.726(5) Å β = 91.535(6)°<br>c = 15.740(6) Å γ = 90°
Volume	1031.1(10) Å^3
Z	2
Density (calculated)	1.293 g cm^{-3}
Absorption coefficient	0.190 mm^{-1}
F(000)	424
Crystal size	0.200 × 0.010 × 0.010 mm^3
Theta range for data collection	1.129 to 27.951°
Index ranges	$-12 \le h \le 13$, $-9 \le k \le 9$, $-20 \le l \le 20$
Reflections collected	18718
Independent reflections	3147 [R(int) = 0.0401]
Completeness to theta = 21.836°	98.9 %
Absorption correction	None
Refinement method	Full-matrix least-squares on F^2
Data / restraints / parameters	3147 / 66 / 181
Goodness-of-fit on F^2	1.122
Final R indices [$I > 2\sigma(I)$]	R_1 = 0.0445, wR_2 = 0.1342
R indices (all data)	R_1 = 0.0502, wR_2 = 0.1414
Extinction coefficient	n/a
Largest difference peak and hole	0.356 and –0.586 e × Å^{-3}

Table S12. Crystal data and structure refinement for **D5(OMe)-A1** single-crystal.

Empirical formula	$C_{16}H_{15}N_3OS_2$
Formula weight	329.43
Temperature	100(2) K
Wavelength	1.54178 Å
Crystal system	Monoclinic
Space group	$C2/m$
Unit cell dimensions	a = 25.050(3) Å α = 90°<br>b = 6.7487(7) Å β = 94.223(5)°<br>c = 9.7228(9) Å γ = 90°
Volume	1639.2(3) Å^3
Z	4
Density (calculated)	1.335 g cm^{-3}
Absorption coefficient	2.980 mm^{-1}
F(000)	688
Crystal size	0.230 × 0.064 × 0.021 mm^3
Theta range for data collection	3.538 to 72.187°.
Index ranges	$-30 \le h \le 30$, $-8 \le k \le 8$, $-12 \le l \le 11$
Reflections collected	16201
Independent reflections	1764 [R(int) = 0.0277]
Completeness to theta = 67.679°	99.8 %
Absorption correction	Semi-empirical from equivalents
Max. and min. transmission	0.7536 and 0.5888
Refinement method	Full-matrix least-squares on F^2
Data / restraints / parameters	1764 / 0 / 131
Goodness-of-fit on F^2	1.048
Final R indices [$I > 2\sigma(I)$]	R_1 = 0.0283, wR_2 = 0.0754
R indices (all data)	R_1 = 0.0300, wR_2 = 0.0773
Extinction coefficient	n/a
Largest diff. peak and hole	0.351 and –0.234 e × Å^{-3}

Table S13. Crystal data and structure refinement for **D6(cPen)-A1** single-crystal.

Empirical formula	$C_{20}H_{21}N_3S_2$
Formula weight	367.52
Temperature	100(2) K
Wavelength	1.54178 Å
Crystal system	Orthorhombic
Space group	$Pna2_1$
Unit cell dimensions	a = 9.8062(9) Å α = 90°<br>b = 27.658(2) Å β = 90°<br>c = 6.8478(8) Å γ = 90°
Volume	1857.3(3) Å^3
Z	4
Density (calculated)	1.314 g cm^{-3}
Absorption coefficient	2.643 mm^{-1}
F(000)	776
Crystal size	0.332 × 0.047 × 0.012 mm^3
Theta range for data collection	4.784 to 72.249°
Index ranges	$-12 \le h \le 12$, $-33 \le k \le 34$, $-7 \le l \le 8$
Reflections collected	26672
Independent reflections	3557 [R(int) = 0.0353]
Completeness to theta = 67.679°	99.9 %
Absorption correction	Semi-empirical from equivalents
Max. and min. transmission	0.7536 and 0.5777
Refinement method	Full-matrix least-squares on F^2
Data / restraints / parameters	3557 / 1 / 230
Goodness-of-fit on F^2	1.073
Final R indices [$I > 2\sigma(I)$]	R_1 = 0.0239, wR_2 = 0.0611
R indices (all data)	R_1 = 0.0246, wR_2 = 0.0618
Absolute structure parameter	0.485(17)
Extinction coefficient	n/a
Largest diff. peak and hole	0.321 and –0.157 e × Å^{-3}

Table S14. Crystal data and structure refinement for **D3(Pyrl)-A1**:**D4(Pyrl)-A1** (54 ± 4):(46 ± 4) co-crystal.

Empirical formula	$C_{19.52}H_{19.54}Cl_{0.47}N_{4.46}O_{0.82}S_2$
Formula weight	410.56
Temperature	100(2) K
Wavelength	0.61992 Å
Crystal system	Monoclinic
Space group	$P2_1/m$
Unit cell dimensions	a = 9.8150(14) Å α = 90°<br>b = 6.762(3) Å β = 91.190(8)°<br>c = 15.801(3) Å γ = 90°
Volume	1048.5(6) Å^3
Z	2
Density (calculated)	1.300 g cm^{-3}
Absorption coefficient	0.226 mm^{-1}
F(000)	428.9
Crystal size	0.200 × 0.100 × 0.010 mm^3
Theta range for data collection	1.124 to 27.729°
Index ranges	$-14 \le h \le 14$, $-9 \le k \le 9$, $-20 \le l \le 20$
Reflections collected	19459
Independent reflections	3154 [R(int) = 0.0403]
Completeness to theta = 21.836°	98.4 %
Absorption correction	None
Refinement method	Full-matrix least-squares on F^2
Data / restraints / parameters	3154 / 168 / 229
Goodness-of-fit on F^2	1.082
Final R indices [$I > 2\sigma(I)$]	R_1 = 0.0682, wR_2 = 0.1928
R indices (all data)	R_1 = 0.0734, wR_2 = 0.1992
Extinction coefficient	n/a
Largest difference peak and hole	0.332 and –0.951 e × Å^{-3}

Table S15. Crystal data and structure refinement for **D1(Pyrl)-A1**:**D3(Pyrl)-A1** (55 ± 1):(45 ± 1) co-crystal.

Empirical formula	$C_{20}H_{24}N_4OS_{1.51}Se_{0.49}$
Formula weight	423.53
Temperature	100(2) K
Wavelength	1.54178 Å
Crystal system	Monoclinic
Space group	$P2_1/m$
Unit cell dimensions	a = 9.8032(9) Å α = 90°<br>b = 6.7908(6) Å β = 90.471(7)°<br>c = 15.8016(19) Å γ = 90°
Volume	1051.90(18) Å^3
Z	2
Density (calculated)	1.337 g cm^{-3}
Absorption coefficient	2.939 mm^{-1}
F(000)	441.6
Crystal size	0.190 × 0.039 × 0.011 mm^3
Theta range for data collection	2.796 to 72.223°
Index ranges	$-12 \le h \le 11$, $-8 \le k \le 7$, $-19 \le l \le 19$
Reflections collected	17654
Independent reflections	2260 [R(int) = 0.0356]
Completeness to theta = 67.679°	100.0 %
Absorption correction	Semi-empirical from equivalents
Max. and min. transmission	0.7536 and 0.5856
Refinement method	Full-matrix least-squares on F^2
Data / restraints / parameters	2260 / 0 / 179
Goodness-of-fit on F^2	1.083
Final R indices [$I > 2\sigma(I)$]	R_1 = 0.0363, wR_2 = 0.0964
R indices (all data)	R_1 = 0.0398, wR_2 = 0.0996
Extinction coefficient	n/a
Largest diff. peak and hole	0.788 and –0.479 e × Å^{-3}

Table S16. Crystal data and structure refinement for **D1(Pyrl)-A1**:**D4(Pyrl)-A1** (51 ± 1):(49 ± 1) co-crystal.

Empirical formula	$C_{19.55}H_{23.55}N_{4.45}OS_{1.45}Se_{0.55}$
Formula weight	426.79
Temperature	100(2) K
Wavelength	1.54178 Å
Crystal system	Monoclinic
Space group	$P2_1/m$
Unit cell dimensions	a = 9.7649(9) Å α = 90°<br>b = 6.7840(7) Å β = 91.382(6)°<br>c = 15.8332(17) Å γ = 90°
Volume	1048.57(18) Å^3
Z	2
Density (calculated)	1.352 g cm^{-3}
Absorption coefficient	3.019 mm^{-1}
F(000)	443.7
Crystal size	0.153 × 0.070 × 0.037 mm^3
Theta range for data collection	5.266 to 72.190°
Index ranges	$-11 \le h \le 12$, $-8 \le k \le 8$, $-19 \le l \le 19$
Reflections collected	19987
Independent reflections	2256 [R(int) = 0.0222]
Completeness to theta = 67.679°	99.9 %
Absorption correction	Semi-empirical from equivalents
Max. and min. transmission	0.7536 and 0.6240
Refinement method	Full-matrix least-squares on F^2
Data / restraints / parameters	2256 / 4 / 181
Goodness-of-fit on F^2	1.089
Final R indices [$I > 2\sigma(I)$]	R_1 = 0.0295, wR_2 = 0.0775
R indices (all data)	R_1 = 0.0296, wR_2 = 0.0776
Extinction coefficient	n/a
Largest diff. peak and hole	0.476 and –0.326 e × Å^{-3}

Table S17. Crystal data and structure refinement for **D3(Bu)-A2** single-crystal.

Empirical formula	$C_{20}H_{24}N_4S_2$
Formula weight	384.55
Temperature	100(2) K
Wavelength	1.54178 Å
Crystal system	Monoclinic
Space group	$P2_1/c$
Unit cell dimensions	a = 12.3265(15) Å α = 90°<br>b = 18.782(2) Å β = 100.417(6)°<br>c = 8.7935(11) Å γ = 90°
Volume	2002.3(4) Å^3
Z	4
Density (calculated)	1.276 g cm^{-3}
Absorption coefficient	2.486 mm^{-1}
F(000)	816
Crystal size	0.270 × 0.218 × 0.081 mm^3
Theta range for data collection	3.646 to 72.276°
Index ranges	$-15 \le h \le 15$, $-23 \le k \le 23$, $-10 \le l \le 9$
Reflections collected	30649
Independent reflections	3924 [R(int) = 0.0286]
Completeness to theta = 67.679°	99.8 %
Absorption correction	Semi-empirical from equivalents
Max. and min. transmission	0.7536 and 0.5500
Refinement method	Full-matrix least-squares on F^2
Data / restraints / parameters	3924 / 0 / 238
Goodness-of-fit on F^2	1.032
Final R indices [$I > 2\sigma(I)$]	R_1 = 0.0291, wR_2 = 0.0780
R indices (all data)	R_1 = 0.0302, wR_2 = 0.0792
Extinction coefficient	n/a
Largest diff. peak and hole	0.430 and –0.207 e × Å^{-3}

Table S18. Crystallographic data of single- and co-crystals of H1-type[a)] merocyanine dyes **D1(Pyrl)-A1**, **D3(Pyrl)-A1**, **D4(Pyrl)-A1**, and their three possible molar 1:1 mixtures, **D5(OMe)-A1**, and **D6(cPen)-A1**. The respective distances/angles correspond to those depicted in Figure 4 and Figure 6. The molar ratio was determined by the relative electron density from XRD data.

Dye	D1(Pyrl)-A1	D3(Pyrl)-A1 [S1]	D4(Pyrl)-A1	D3(Pyrl)-A1: D4(Pyrl)-A1	D3(Pyrl)-A1: D1(Pyrl)-A1	D4(Pyrl)-A1: D1(Pyrl)-A1	D5(OMe)-A1	D6(cPen)-A1
$d_{\pi-\pi}$ [Å]	3.41	3.37	3.36	3.38	3.40	3.39	3.37	3.42 [b)]
a, b, c [Å]	9.8, 6.8, 15.9	6.8, 9.8, 15.1	9.7, 6.7, 15.7	9.8, 6.8, 15.8	9.8, 6.8, 15.8	9.8, 6.8, 15.8	25.1, 6.7, 9.7	9.8, 27.7, 6.8
α, β, γ [°]	90, 91, 90	77, 82, 89	90, 91, 90	90, 91, 90	90, 90, 90	90, 91, 90	90, 94, 90	90, 90, 90
Θ_{slip} [°]	70	67	68	67	69	69	65	62
Molar ratio	-	-	-	54:46 [c)]	45:55 [d)]	49:51 [e)]	-	-

[a)] Single-crystal data of **D2(Pyrl)-A1** is not available due to its photoinstability. [b)] This value corresponds to half the distance to the second-nearest neighbour within the π-stack, as the nearest neighbour distance could not be accurately determined due to the twisted, non-planar cyclopentyl (cPen) moiety. [c)-e)] The deviations of the molar mixture ratios stemming from a comparison of the respective electron densities are [c)] ± 4, [d)] ± 1, and [e)] ± 1.

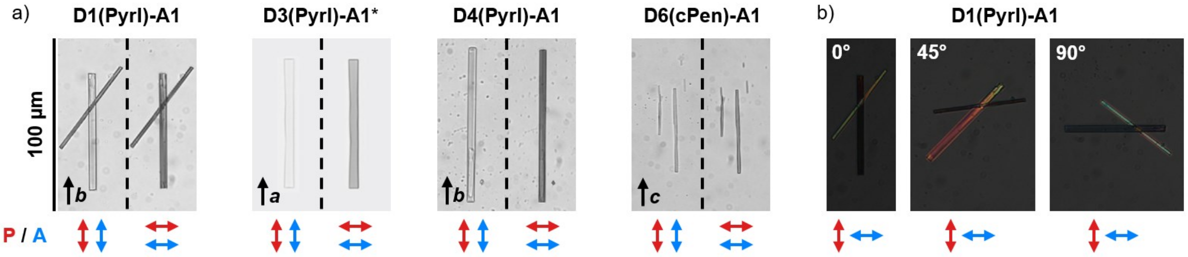

Figure S20. POM images (black and white for better visibility) of microcrystals of H1-type aggregating dyes grown from 1 mg mL^{-1} $CHCl_3$ solutions using methanol as a counter solvent on quartz substrates. a) Images of individual single-crystals of compounds **D1(Pyrl)-A1**, **D3(Pyrl)-A1**, **D4(Pyrl)-A1**, and **D6(cPen)-A1** with both polarizer (P, red) and analyzer (A, blue) oriented either parallel (left) or orthogonal (right) to the long crystal axis of the largest visible crystal; this axis, corresponding to the crystal growth direction, is denoted with the respective unit cell axis from the respective single-crystal structure. b) Images of the single-crystal of **D1(Pyrl)-A1** with cross-polarized P and A at different stages of substrate rotation showing birefringence at 45°. * The data for **D3(Pyrl)-A1** was reprinted with permission from the © 2017 American Chemical Society from ref. S1.

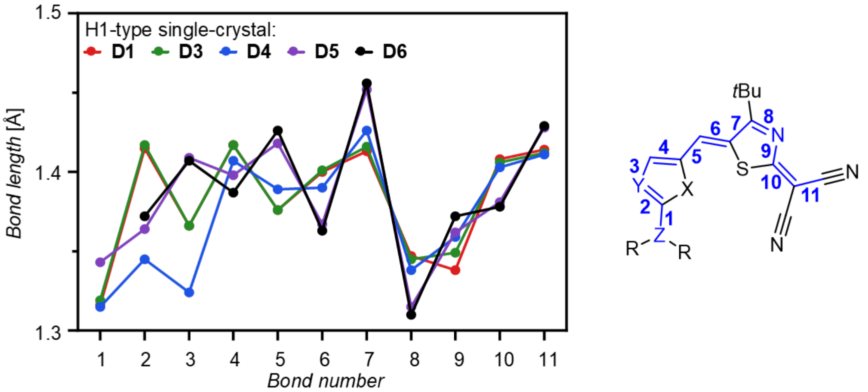

Figure S21. Bond lengths determined from the single-crystal structures of H1-type dyes with donor units **D1** (red), **D3** (green), **D4** (blue), **D5** (violet), and **D6** (black) including their base chemical structure with bonds contributing to the delocalized π-system accordingly labelled.

7 Thin Film Diffraction

Thin Film X-Ray Diffraction (TF-XRD):

Table S19. Out-of-plane lattice distances ($d_{TF\text{-}XRD}$) of pristine and mixed merocyanine thin films on quartz substrates as determined from the 2Θ angles from TF-XRD measurements. Where available, the corresponding Miller plane from the single- or co-crystal structure corresponding to $d_{TF\text{-}XRD}$ is listed. Unless denoted otherwise, all measurements were collected from thin films spin-coated from $CHCl_3$ solutions and annealed under conditions optimized for the respective compound or mixture.

**Aggregate type**	**Layer** (Mixtures in molar ratios)	2Θ [°]	$d_{TF\text{-}XRD}$ [Å]	**Corresponding Miller plane from crystal structure**
**H1-type**	**D1(Pyrl)-A1**	6.0	14.7	(0 0 1)
	**D3(Pyrl)-A1**	6.0	14.7	(0 0 1)
	**D4(Pyrl)-A1**	6.1	14.5	(0 0 1)
	**D5(OMe)-A1** *	7.1	12.5	(2 0 0)
	**D6(cPen)-A1**	6.4	13.9	(0 2 0)
	**D1(Pyrl)-A1**:**D4(Pyrl)-A1** 1:1	6.0	14.7	(0 0 1)
**H2-type**	**D1(Bu)-A2**	5.1	17.2	n/a
	**D3(Bu)-A2**	5.3	16.7	(0 1 0)
	**D4(Bu)-A2**	5.3	16.7	n/a
	**D1(Bu)-A2**:**D4(Bu)-A2** 1:1	5.2	17.1	n/a
**J-type**	**D1(Hex)-A1**	3.9	22.6	n/a
	**D3(Hex)-A1**	3.9	22.7	
	**D4(Hex)-A1**	4.1	21.5	
	**D1(Hex)-A1**:**D4(Hex)-A1** 1:1	4.0	22.1	

*: Thin film prepared by thermal sublimation onto substrates at 60 °C.

Selected Area Electron Diffraction (SAED):

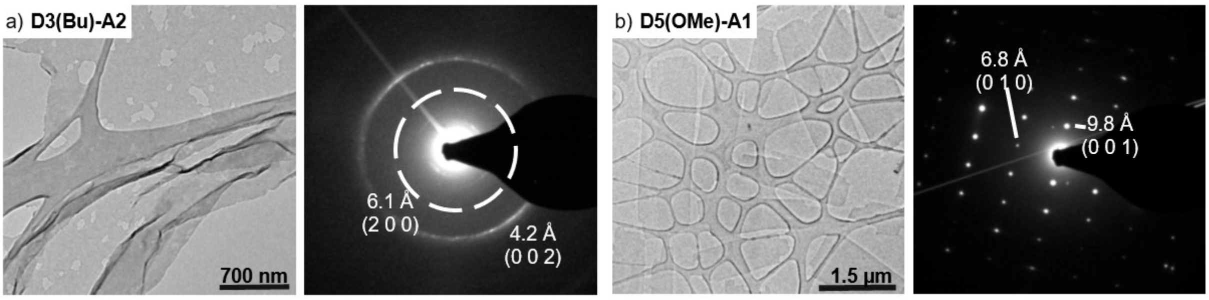

Figure S22. TEM images (left) and SAED patterns (right) of polycrystalline annealed thin films of H2-type **D3(Bu)-A2** (a) and H1-type **D5(OMe)-A1** (b). In the SAED patterns, relevant reflexes to allocate the correct crystal structure polymorph are marked with the corresponding lattice distance and Miller plane from the respective single-crystal structure.

8 Calculated Absorption Spectra of Solid-State Assemblies

H2-type Assembly of D3(Bu)-A2:

To verify that the packing arrangement of **D3(Bu)-A2** results in H2-type coupled aggregates, single-point TD-DFT calculations of individual dimer, tetramer, and hexamer π-stacks from this crystal structure were performed (Figure S23, Table S20). Similar calculations have previously been used to verify the nature of the Coulomb coupling in the H1-type crystal structure of **D3(Pyrl)-A1**.[S1] For **D3(Bu)-A2**, the highest calculated f_{osc} is always located in the most blue-shifted transition (= highest state within the exciton band), which analogously as for **D3(Pyrl)-A1** verifies the presence of H-type Coulomb coupling. Any additional contributions to the overall coupling by CT interactions[S43,S27,S44] could be excluded through ADF calculations, which revealed overall negligible effective t_h values of only 135 cm^{-1} (17 meV) to the nearest neighbors. The overall lower coupling and $\tilde{\nu}_{shift}$ compared to **D3(Pyrl)-A1** can be explained by the aggregate geometry and the scaling of Coulomb coupling by $R_{\mu eg}^{-3}$, where $R_{\mu eg}$ is the center-to-center distance between neighboring μ_{eg} vectors. For **D3(Pyrl)-A1**, the chromophores adopt a compact 1D card-stack with a co-planar arrangement, with coupling thus saturating in an octamer dye stack,[S1] as is also observed for similar merocyanine dyes.[S45] Hereby, $R_{\mu eg}$ to the second nearest neighbor is 6.8 Å. This distance is significantly increased to 8.8 Å for **D3(Bu)-A2**, with the resulting Coulomb coupling to the second nearest neighbor thus dropping to 45 % according to Kasha's point-dipole approximation. With the additional tilt of 7° of neighboring molecules towards each other, this results in overall weaker and less-extended H2-type coupling with more broadened absorption features for the **Dx(Bu)-A2** series ($fwhm_{opt}$: 61–89 nm, 1 850–2 275 cm^{-1}). The isostructural chromophores with different donor units (**D1** and **D4**) adopt the same packing arrangement, which results in the tunability of the H2-type coupled aggregate bands (λ_{opt}: 575–629 nm).

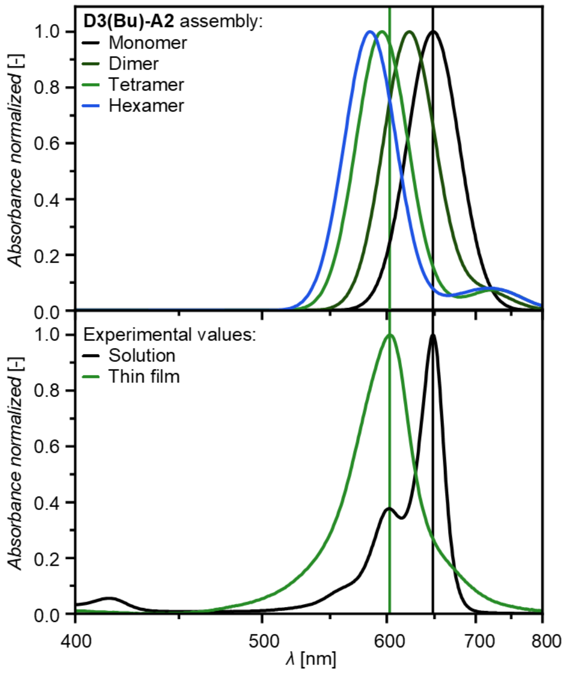

Figure S23. Calculated UV-Vis-NIR absorption spectra of differently-sized π-stacked assemblies (monomer in black, dimer in dark green, tetramer in light green, hexamer in blue) of **D3(Bu)-A2** compared to the experimental spectra of its monomer in $CHCl_3$ (black) and an annealed thin film on a quartz substrate (green). The calculated spectra (top) were shifted by −5 044 cm^{-1} so that the calculated monomer transition matches the experimentally determined one (bottom). The structures used for calculation are taken from the π-stack within the H2-type crystal structure of **D3(Bu)-A2**.

Table S20. Optical transitions calculated by single-point TD-DFT calculations of **D3(Bu)-A2** as a monomer and in dimer, tetramer, and hexamer π-stacked assemblies as taken from its single-crystal structure. Calculated wavenumber values were shifted by −5 044 cm^{-1} to match the experimental value of the monomer A_{00} absorption in $CHCl_3$ solution at 15 425 cm^{-1} with $\tilde{\nu}_{shift}$ denoting the calculated spectral shift compared to the A_{00} monomer transition. The most prominent transition with the largest calculated relative oscillator strength f_{osc} is marked in bold for each type of assembly.

Assembly	Calculated value	Transition					
		1	2	3	4	5	6
Monomer (1)	$\tilde{\nu}$ [cm^{-1}]	**15 425**	-	-	-	-	-
	$\tilde{\nu}_{shift}$ [cm^{-1}]	-	-	-	-	-	-
	relative f_{osc} [%]	**100**	-	-	-	-	-
Dimer (2)	$\tilde{\nu}$ [cm^{-1}]	14 049	**16 065**	-	-	-	-
	$\tilde{\nu}_{shift}$ [cm^{-1}]	−1 376	**+640**	-	-	-	-
	relative f_{osc} [%]	7	**100**	-	-	-	-
Tetramer (4)	$\tilde{\nu}$ [cm^{-1}]	13 657	14 009	15 110	**16 794**	-	-
	$\tilde{\nu}_{shift}$ [cm^{-1}]	−1 768	−1 416	−315	**+1 369**	-	-
	relative f_{osc} [%]	6	2	1	**100**	-	-
Hexamer (6)	$\tilde{\nu}$ [cm^{-1}]	13 580	13 684	14 030	14 736	15 873	**17 110**
	$\tilde{\nu}_{shift}$ [cm^{-1}]	−1 845	−1 741	−1 395	−689	+448	**+1 685**
	relative f_{osc} [%]	5	0	2	3	0	**100**

J-type Assembly of D3(Oct)-A1:

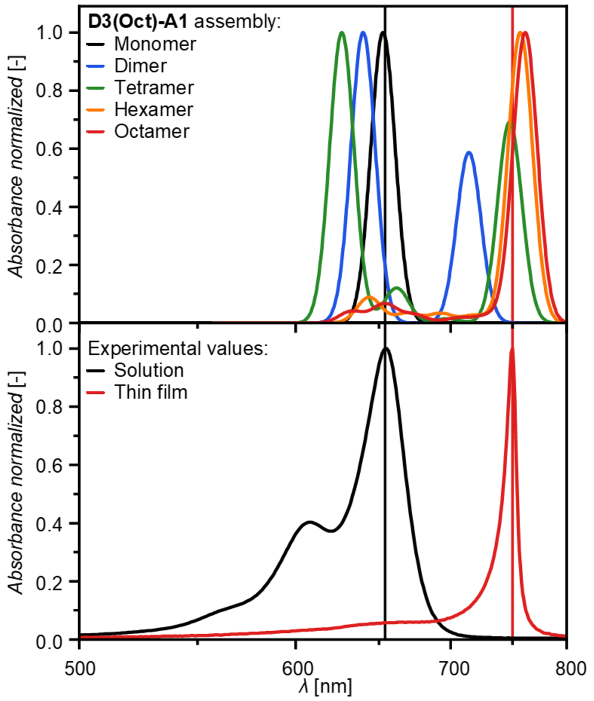

Figure S24. Calculated UV-Vis-NIR absorption spectra of differently-sized π-stacked assemblies (monomer in black, dimer in blue, tetramer in green, hexamer in orange, octamer in red) of **D3(Oct)-A1** compared to the experimental spectra of its monomer in $CHCl_3$ (black) and an annealed thin film on a quartz substrate (red). The calculated spectra (top) were shifted by −5 861 cm^{-1} so that the calculated monomer transition matches the experimentally determined one (bottom). The structures used for calculation are taken from the J-strand within the J-type crystal structure of **D3(Oct)-A1**.

Table S21. Optical transitions calculated by single-point TD-DFT calculations of **D3(Oct)-A1** as a monomer and in dimer, tetramer, hexamer, and octamer zig-zag-strand assemblies as taken from its single-crystal structure. Calculated wavenumber values were shifted by −5 861 cm^{-1} to match the experimental value of the monomer A_{00} absorption in $CHCl_3$ solution at 15 425 cm^{-1} with $\tilde{\nu}_{shift}$ denoting the calculated spectral shift compared to the A_{00} monomer transition. The most prominent transition with the largest calculated relative oscillator strength f_{osc} is marked in bold for each type of assembly.

Assembly	Calculated value	Transition							
		1	2	3	4	5	6	7	8
Monomer (1)	$\tilde{\nu}$ [cm^{-1}]	**15 325**	-	-	-	-	-	-	-
	$\tilde{\nu}_{shift}$ [cm^{-1}]	-	-	-	-	-	-	-	-
	relative f_{osc} [%]	**100**	-	-	-	-	-	-	-
Dimer (2)	$\tilde{\nu}$ [cm^{-1}]	**14 004**	**15 631**	-	-	-	-	-	-
	$\tilde{\nu}_{shift}$ [cm^{-1}]	**−1 321**	**+306**	-	-	-	-	-	-
	relative f_{osc} [%]	**64**	**100**	-	-	-	-	-	-
Tetramer (4)	$\tilde{\nu}$ [cm^{-1}]	**19 235**	20 199	20 978	**21 819**	-	-	-	-
	$\tilde{\nu}_{shift}$ [cm^{-1}]	**−1 950**	−986	−208	**+634**	-	-	-	-
	relative f_{osc} [%]	**79**	2	13	**100**	-	-	-	-
Hexamer (6)	$\tilde{\nu}$ [cm^{-1}]	**13 211**	13 912	14 435	14 947	15 538	16 273	-	-
	$\tilde{\nu}_{shift}$ [cm^{-1}]	**−2 114**	−1 413	−890	−378	+213	+948	-	-
	relative f_{osc} [%]	**100**	3	3	3	8	0	-	-
Octamer (8)	$\tilde{\nu}$ [cm^{-1}]	**13 137**	13 709	14 148	14 489	14 861	15 119	15 303	15 813
	$\tilde{\nu}_{shift}$ [cm^{-1}]	**−2 188**	−1 616	−1 177	−836	−464	−206	−22	+488
	relative f_{osc} [%]	**100**	2	2	0	3	0	6	4

9 Calculated Frontier Orbitals of Co-Crystals

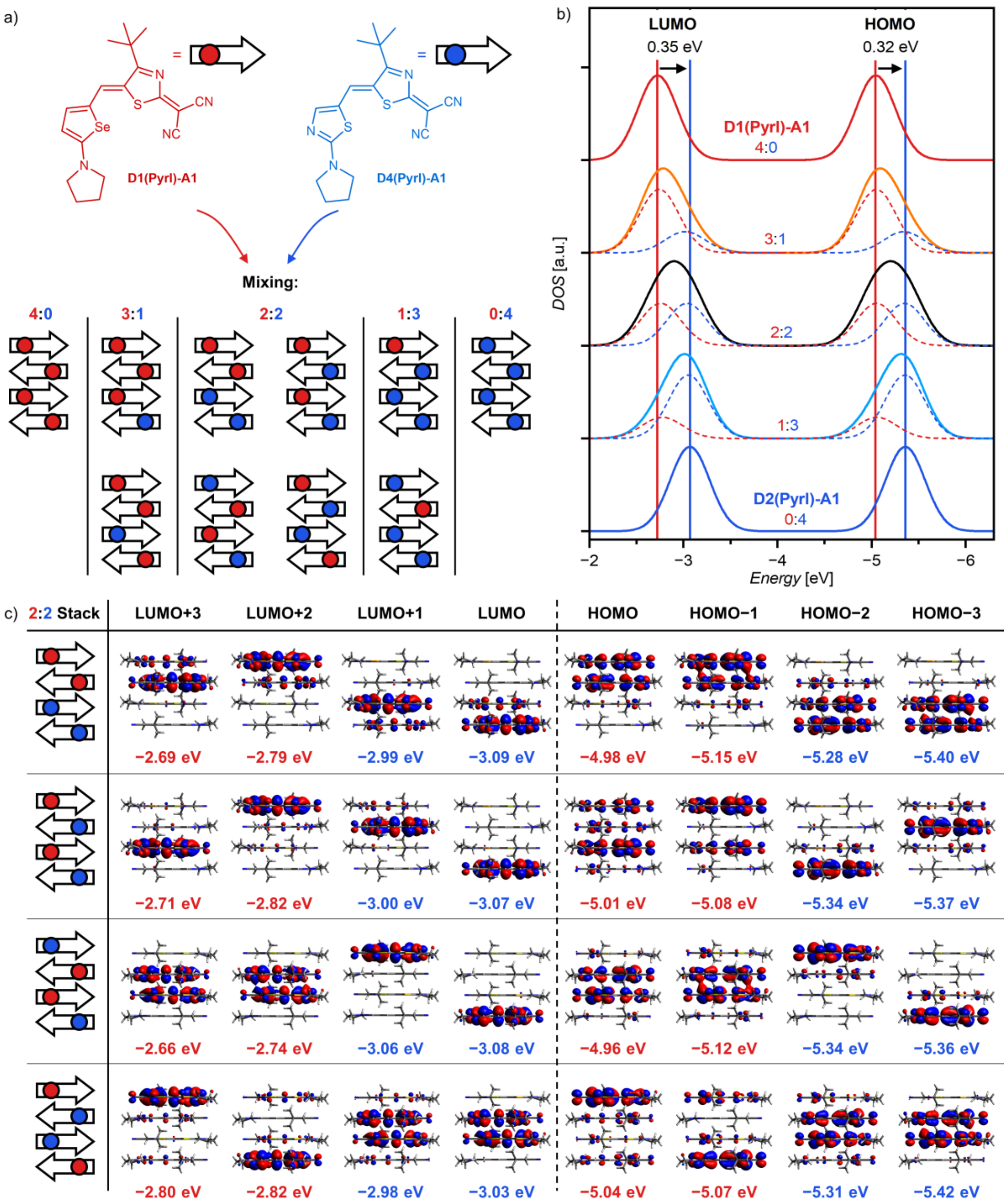

Figure S25. Results for the ground state calculations for H1-type co-crystals of **D1(Pyrl)-A1**:**D4(Pyrl)-A1** performed at a B3LYP/def2SVP single-point level of theory. a) Schematic depiction of all possible 4-stack arrangement structures used for the calculations generated from the single-crystal of **D1(Pyrl)-A1**. b) Resulting HOMO and LUMO density-of-states (DOS) at different mixing ratios with a Gaussian broadening of 500 meV. c) Depiction of the first four LUMO and HOMO levels for each possible stack arrangement at the exemplarily chosen 2:2 mixing ratio. The dashed lines in b) show the broadened average frontier orbital energies of the respective components with their intensities scaled to match the mixing ratio, and the solid lines show the respective summation of the latter two and indicate the resulting overall DOS at the individual mixing ratio; the vertical lines mark the respective positions of the maxima of the density of states of pristine 4-stacks of **D1(Pyrl)-A1** and **D4(Pyrl)-A1**. The energetic values of the orbitals in c) are colored according to the merocyanine dye **D1(Pyrl)-A1** (red) or **D4(Pyrl)-A1** (blue) they are allocated to.

Table S22. Calculated lattice energies (E_{latt}) at a CE-B3LYP/6-31G(d,p) level of theory of compounds **D1(Pyrl)-A1**, **D3(Pyrl)-A1**, and **D4(Pyrl)-A1**.

	**Parameters**				**Contributions to attractive interactions** [%]		
**Compound**	μ_g [D]	μ_g [%]	E_{latt} [kJ mol^{-1}]	E_{latt} [%]	**Electrostatic**	**Polarization**	**Dispersion**
**D1(Pyrl)-A1**	12.4	95	−183	92	25	13	62
**D3(Pyrl)-A1**	13.0	100	−199	100	23	14	63
**D4(Pyrl)-A1**	9.5	73	−128	64	22	16	62

10 Supporting Data for J-Type Crystal Structure

Notes Regarding "Wrong" Polymorph Formation of D3(Hex)-A1:

While a single-crystal structure of **D3(Hex)-A1** has been previously published by Liess *et al.*,[S3] by SAED and TF-XRD measurements it became obvious, that this packing arrangement does not correspond to the polymorph present in J-type coupled thin films. Instead, when grown from solution, **D3(Hex)-A1** rather forms individual dimers with anti-parallely aligned chromophores due to the flexibility of the hexyl substituents. By enclosing the dimer π-surfaces this in turn also prohibits further extended aggregation. The J-aggregate is thus seemingly only formed on substrates, presumably due to necessary surface-dye interactions. We attempted a plethora of further crystallization techniques, including solvent and vapor diffusion using various (anti-)solvents, growth on surfactant monolayers, high-vacuum and argon-flow gradient sublimation,[S46] in-air sublimation,[S47] Bridgman and Lipsett crystallization,[S48,S49] as well as growth in molten hydrocarbons.[S50] All attempts exclusively revealed the "wrong" polymorph of **D3(Hex)-A1**, with the cancellation of μ_g seemingly outweighing the tendency for extended J-aggregate formation as observed on substrate surfaces. Accordingly, we approximated the single-crystal structure of **D3(Hex)-A1** by using derivates with longer aliphatic chains such as **D3(Oct)-A1**.

Details on the obtained J-Type Crystal Structure of D3(Oct)-A1:

A determination of the slip-stacked J-type packing arrangement of **D3(Hex)-A1** by single-crystal XRD was not possible because this compound exclusively crystallized in a different polymorph exhibiting individual isolated dimer units as described above. While a single-crystal structure for **D3(Pr)-A1**, which contains propyl instead of hexyl substituents at the amino-thiophene donor moiety, with similar J-type coupling in the solid state has been previously reported (Figure S29),[S1] this dye exhibits a significantly broader ($fwhm_{opt}$: 650 cm^{-1} vs. 290 cm^{-1}) absorption band (Figure S26c). To thus further approximate the packing structure of **D3(Hex)-A1**, we synthesized compounds **D3(Oct)-A1** and **D3(Dec)-A1** bearing even longer octyl and decyl chains, respectively (Figure S26a; see the corresponding supporting information sections for details on synthesis and characterization), to increase van-der-Waals interactions between the aliphatic chains and promote the growth of 2D J-type single-crystals.[S51] Indeed, all derivatives, irrespective of the linear chain length (Pr → Bu → Hex → Oct → Dec), show similar J-type coupled absorption bands with almost identical λ_{opt} values of 746–751 nm. With increasing chain length, the $fwhm_{opt}$ steadily decreases from 650 to 210 cm^{-1} (36 to 12 nm), seeming to saturate for the decyl substituents. Additional TF-XRD measurements show a stepwise shift of the 2Θ reflex towards lower values with increasing chain length (Figure S26, Table S23). Correspondingly, also the maximum absorbance increases with similar absorption band integral values

in thin films spin-coated from equal molar concentrations, which is indicative for an increase in the J-type resonant oscillator strength (Figure S27). Accordingly, we could conclude that all derivatives must show similar J-type packing arrangements as **D3(Hex)-A1**.

The single-crystal structure of **D3(Oct)-A1** (Figure 4c, Figure S30, Table S25) reveals the expected J-type coupled strands with a similar zig-zag arrangement of the dimerized chromophores as for previously reported **D3(Pr)-A1**.[S1] Due to the elongated alkyl chains, these do not assemble next to each other but are rather isolated from each other through the alkyl chains, forming a lamellar packing arrangement. Within these individual lamellae, the π-stacked J-strands are more isolated from one another, thus reducing parasitic out-of-strand coupling and thereby leading to sharper J-bands. A similar effect has been shown in literature for a set of quinoidal indacenodithiophene compounds.[S52] Upon lengthening of their alkyl chain substituents from 2-ethylhexyl to hexadecyl, these dyes showed similar band narrowing of $fwhm_{opt}$ from 142 to 22 nm in their solid-state J-aggregate thin films at the same λ_{opt}. This observation could be explained by single-crystal data, showing an almost identical neighboring chromophore arrangement within the extended aggregate but an increasing lamellar separation of individual J-strands upon chain elongation. The crystal structure of **D3(Oct)-A1** was verified by TF-XRD to be the correct J-type polymorph also present in annealed thin films (Figure S28b) and thus similar to that of **D3(Hex)-A1**. Compared to **D3(Pr)-A1**, crystals of **D3(Oct)-A1** adopt an edge-on orientation on a substrate surface. Hereby the J-strands are analogously aligned parallel to the substrate surface and therefore equal λ_{opt} values are observed. Single-point TD-DFT calculations of the J-strand of **D3(Oct)-A1** performed analogously as previous ones for **D3(Pr)-A1** demonstrate an equal increase in the calculated J-type resonant oscillator strength upon strand elongation up to an octamer arrangement (Figure S24, Table S21).[S1] Due to the more narrowed absorption band of **D3(Hex)-A1** compared to **D3(Pr)-A1** and its similar TF-XRD signal pattern as for the derivatives with longer aliphatic chains (Figure S28a, Table S24), we thus tentatively also assume an edge-on orientation for **D3(Hex)-A1** like for **D3(Oct)-A1** in relation to the substrate surface.

J-Type Thin Film Properties:

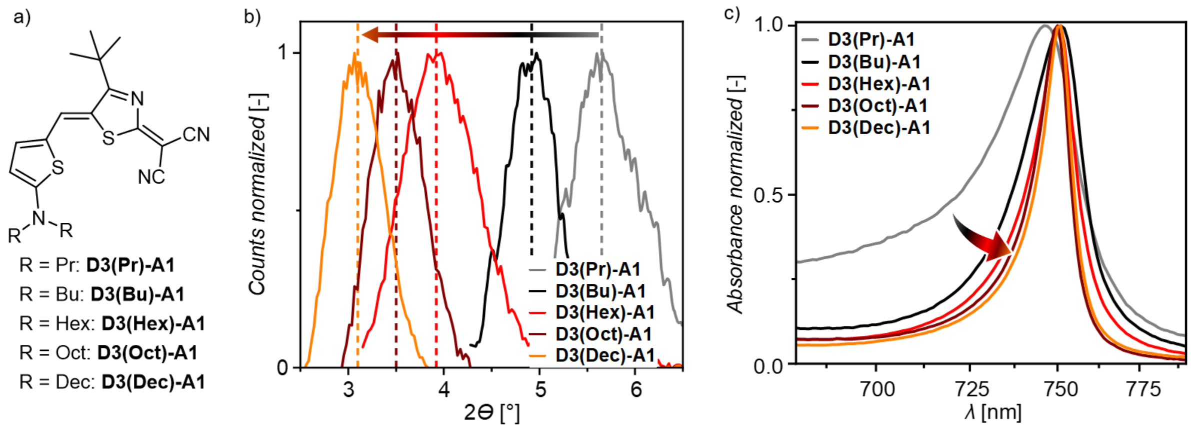

Figure S26. a) General chemical structure of the **D3(R)-A1** dyes with varying alkyl chain length that display J-type coupling in their respective annealed thin films. b) and c) depict cut-outs of the normalized TF-XRD patterns showing the primary reflex peak as well as the normalized absorption spectra of thin films annealed at 130 °C for 5 min, respectively; the arrows indicate the spectral shift and change in band shape upon alkyl chain lengthening.

Table S23. Optical properties and out-of-plane lattice distances ($d_{TF\text{-}XRD}$) of annealed thin films (130 °C for 5 min) on quartz substrates of J-type aggregating merocyanine dyes **D3(R)-A1** with varying alkyl chain length.

Dye	λ_{opt} [nm]	$fwhm_{opt}$ [nm, cm^{-1}]	$d_{TF\text{-}XRD}$ [Å]
**D3(Pr)-A1**	746	36, 650	15.2–15.3
**D3(Bu)-A1**	751	23, 400	17.7–18.0
**D3(Hex)-A1**	750	16, 290	22.7
**D3(Oct)-A1**	750	13, 220	25.1
**D3(Dec)-A1**	751	12, 210	28.5

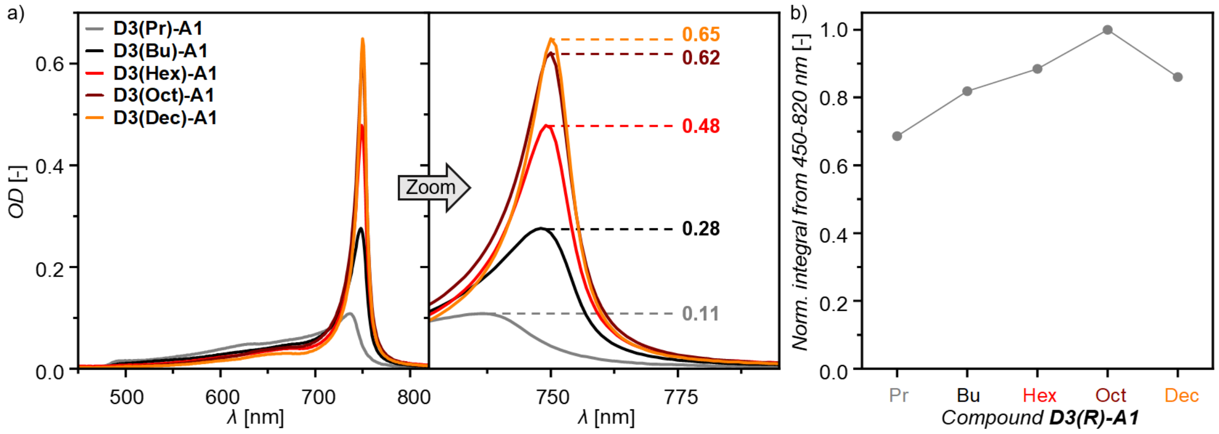

Figure S27. a) UV-Vis-NIR absorption spectra of thin films annealed at 130 °C for 5 min of J-type aggregating dye **D3(R)-A1** spin-coated from 4 mM $CHCl_3$ solutions with varying chain lengths Pr (grey), Bu (black), Hex (red), Oct (brown), and Dec (orange), alongside a zoom-in around the J-band maximum with the denoted maximum *OD* values. b) Normalized integral values of the absorption spectra shown in a) integrated from 450–820 nm.

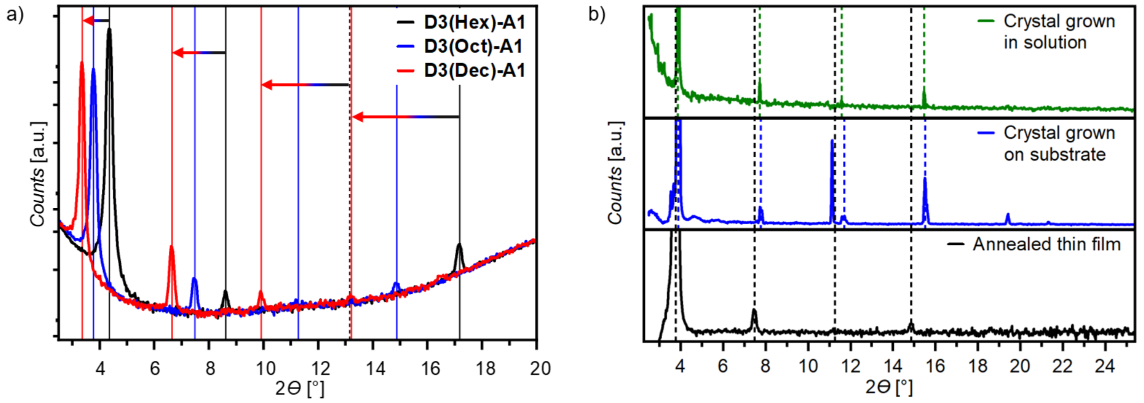

Figure S28. a) TF-XRD patterns of thin films annealed at 130 °C for 5 min of J-type dyes **D3(Hex)-A1** (black), **D3(Oct)-A1** (blue), and **D3(Dec)-A1** (red); the colored vertical lines mark the respective reflex peak positions; the arrows highlight the shift of the reflex peaks upon alkyl chain elongation; the dashed black vertical line marks a peak position which is absent for **D3(Hex)-A1**; the peak data are listed in Table S24. b) Baselined TF-XRD spectra of different samples of **D3(Oct)-A1**: Its single-crystal used for crystal structure analysis and transferred onto an Si/SiO_2 wafer in *DuPont*™ *Krytox*® oil (top, green); an assembly of single-crystals grown on an Si/SiO_2 wafer from $CHCl_3$ using methanol as counter solvent (middle, blue); its annealed thin film as shown in figure a); the dashed vertical lines correspond to the respective reflex peak positions belonging to the same signal set; the single-crystals of **D3(Oct)-A1** grown on the wafer (blue) were also measured by single-crystal XRD and confirmed to be the same polymorph as the single-crystal grown in solution (green).

Table S24. TF-XRD data of J-type dyes **D3(Hex)-A1**, **D3(Oct)-A1**, and **D3(Dec)-A1** as shown in Figure S28.

Reflex number	D3(Hex)-A1		D3(Oct)-A1		D3(Dec)-A1	
	**2θ [°]**	$d_{TF\text{-}XRD}$ **[Å]**	**2θ [°]**	$d_{TF\text{-}XRD}$ **[Å]**	**2θ [°]**	$d_{TF\text{-}XRD}$ **[Å]**
**1**	4.36	20.3	3.78	23.4	3.36	26.3
**2**	8.62	10.2	7.49	11.8	6.65	13.3
**3**	n/a	n/a	11.28	7.8	9.91	8.9
**4**	17.18	5.2	14.88	5.9	13.22	6.7

J-Type Single-Crystal Structures:

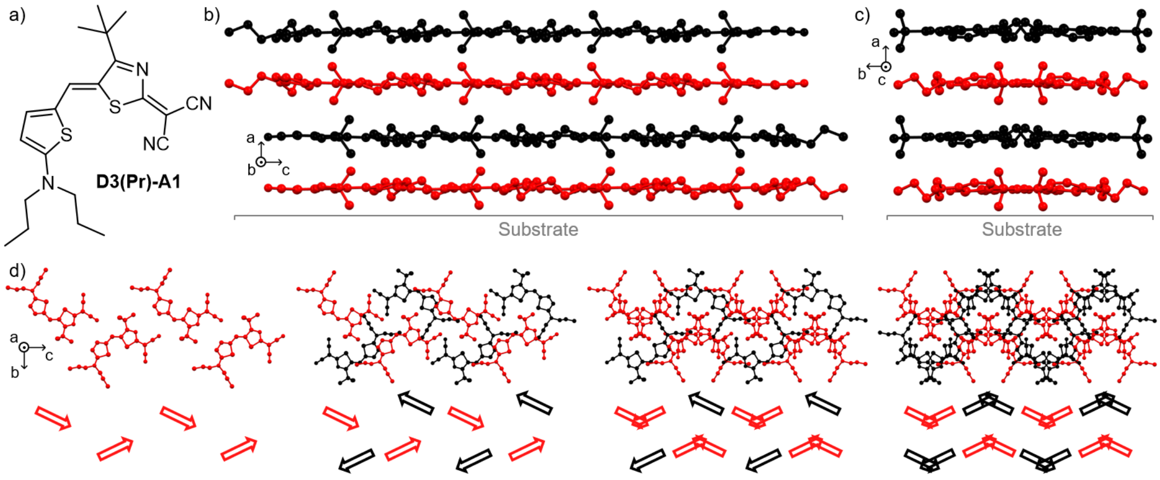

Figure S29. Single-crystal structure of J-type coupled merocyanine **D3(Pr)-A1** according to ref. S1 (CCDC 1496527). a) Chemical structure of the dye. b) and c) Orientation of the J-strands in respect to a substrate surface as determined by TF-XRD in annealed spin-coated thin films on quartz substrates with both a side view and a view along the strand, respectively. d) Top view onto individual layers of two parallel J-strands as well as a schematic orientation of the dipole moments μ_g (arrows). Hydrogen atoms were omitted for clarity.

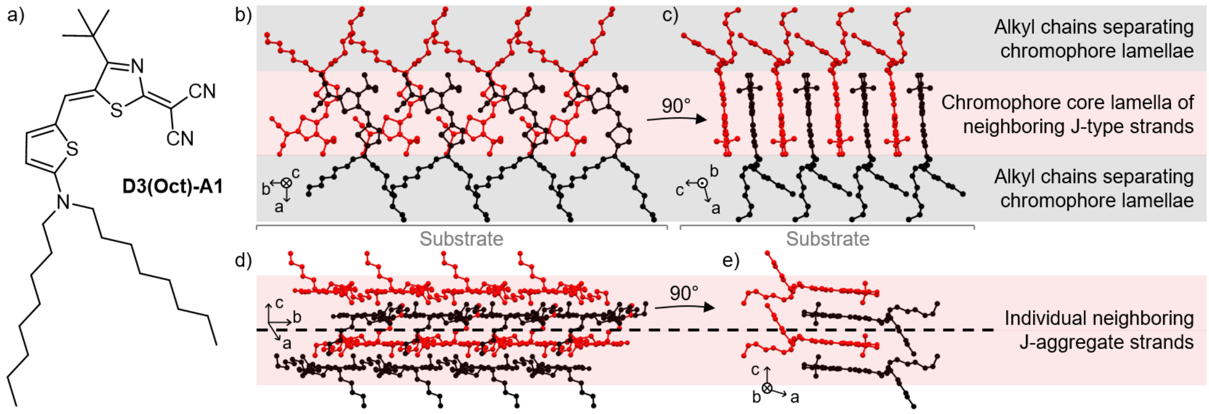

Figure S30. Single-crystal structure of J-type coupled merocyanine **D3(Oct)-A1**. a) Chemical structure of the dye. b) and c) Orientation of the J-strands in respect to a substrate surface as determined by TF-XRD in annealed spin-coated thin films on quartz substrates with both a top view and a view along the strands, respectively. d) and e) Side view onto individual layers of two parallel J-strands. In b)-e) the individual aliphatic and J-aggregate lamellar layers are described and highlighted in grey and red, respectively. Hydrogen atoms were omitted for clarity.

Table S25. Crystal data and structure refinement for **D1(Oct)-A1** single-crystal

Empirical formula	$C_{31}H_{46}N_4S_2$	
Formula weight	538.84	
Temperature	100(2) K	
Wavelength	1.54178 Å	
Crystal system	Monoclinic	
Space group	$P2_1/c$	
Unit cell dimensions	a = 22.785(3) Å	α = 90°
	b = 10.0123(9) Å	β = 101.171(6)°
	c = 13.6943(14) Å	γ = 90°
Volume	3064.9(6) Å^3	
Z	4	
Density (calculated)	1.168 g cm^{-3}	
Absorption coefficient	1.755 mm^{-1}	
F(000)	1168	
Crystal size	0.205 × 0.167 × 0.054 mm^3	
Theta range for data collection	3.955 to 72.352°	
Index ranges	$-28 \le h \le 28$, $-12 \le k \le 12$, $-16 \le l \le 14$	
Reflections collected	65389	
Independent reflections	6044 [R(int) = 0.0433]	
Completeness to theta = 67.679°	99.9 %	
Absorption correction	Semi-empirical from equivalents	
Max. and min. transmission	0.7536 and 0.6200	
Refinement method	Full-matrix least-squares on F^2	
Data / restraints / parameters	6044 / 598 / 470	
Goodness-of-fit on F^2	1.097	
Final R indices [$I > 2\sigma(I)$]	R_1 = 0.0466, wR_2 = 0.1143	
R indices (all data)	R_1 = 0.0505, wR_2 = 0.1174	
Extinction coefficient	n/a	
Largest diff. peak and hole	0.406 and –0.247 e × Å^{-3}	

11 Organic Electronics Devices

Organic thin-film transistors (OTFTs):

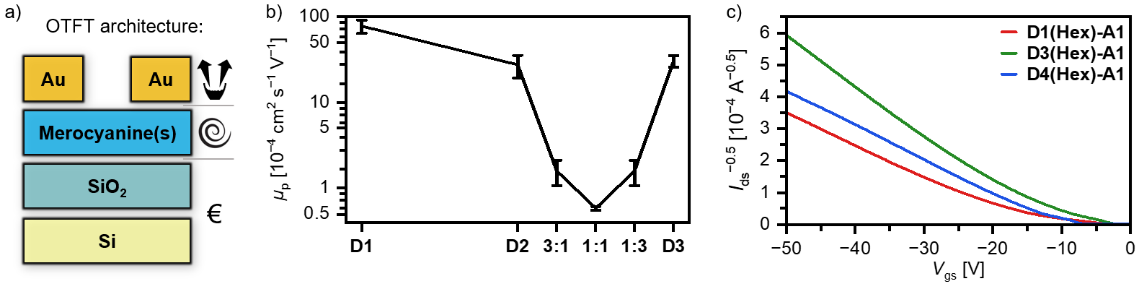

Figure S31. a) Schematic depiction of the bottom-gate, top-contact architecture used for OTFT devices of the J-type **Dx(Hex)-A1** dye series; the Si/SiO_2 substrate is commercially available, the merocyanine layer is deposited by spin-coating and successive annealing (130 °C for 5 min), and the Au electrodes are deposited by thermal evaporation. b) Mobility μ_p values of OTFTs with various **Dx(Hex)-A1** dye compositions and molar mixtures thereof. c) Transfer curves of OTFTs with pristine **Dx(Hex)-A1** semiconductor layers.

Table S26. Device characteristics of spin-coated and annealed (130 °C for 5 min) OTFT devices of **D1(Hex)-A1**, **D3(Hex)-A1**, **D4(Hex)-A1**, and various mixtures thereof. Mobility data was determined in the saturation regime and averaged over at least ten devices across two substrates.

Dye (mixture)	μ_p [cm^2 V^{-1} s^{-1}]	μ_{max} [cm^2 V^{-1} s^{-1}]	V_{th} [V]	I_{on}/I_{off} [-]
**D1(Hex)-A1**	$(30 \pm 5) \times 10^{-4}$	3×10^{-3}	−15	10^6
**D3(Hex)-A1**	$(77 \pm 13) \times 10^{-4}$	8×10^{-3}	−13	10^6
**D4(Hex)-A1**	$(27 \pm 8) \times 10^{-4}$	4×10^{-3}	−10	10^6
**D1(Hex)-A1:D4(Hex)-A1 3:1**	$(16 \pm 5) \times 10^{-5}$	8×10^{-3}	−10	10^3
**D1(Hex)-A1:D4(Hex)-A1 2:2**	$(6 \pm 1) \times 10^{-5}$	8×10^{-3}	−3	10^5
**D1(Hex)-A1:D4(Hex)-A1 1:3**	$(16 \pm 5) \times 10^{-5}$	8×10^{-3}	−10	10^3

Organic photodiodes (OPDs):

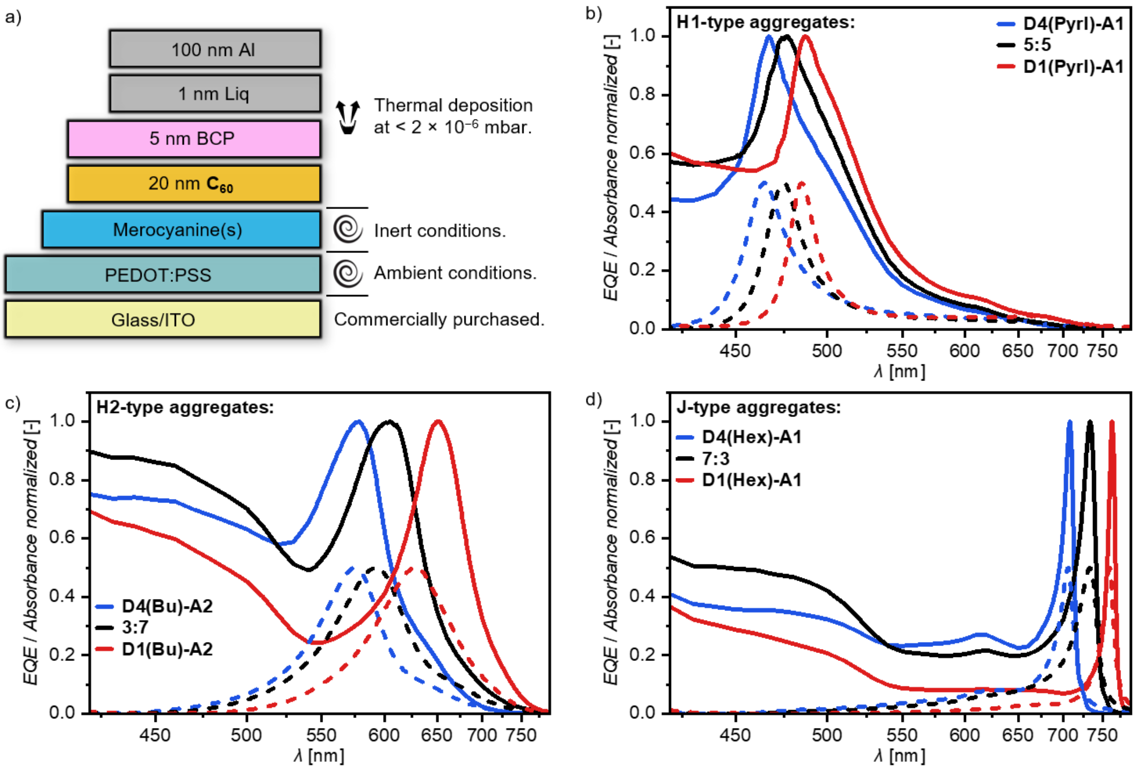

Figure S32. a) Schematic depiction of the employed OPD device architecture. b)-d) respectively show the normalized OPD *EQE* spectra (solid lines) compared to the normalized absorption spectra of the respective pristine/mixed merocyanine thin films of the three aggregate series H1, H2, and J (dashed lines; Figure S15); the mixture ratios were chosen according to Table S8 to yield absorption bands roughly energetically centered between the two pristine λ_{opt} values.

Table S27. Figures of merit of OPD devices [a)] of all three different aggregate series (H1, H2, J) with merocyanine dyes with donor moieties **D4**, **D1**, and mixtures thereof as electron donors. The mixture ratios were chosen according to Table S8 to yield absorption bands roughly energetically centered between the two pristine λ_{opt} values.

Type	Dye (mixture)	EQE_{max} [%]	R_{max} [mA W^{-1}]	λ_{EQE} [nm, cm^{-1}]	λ_{opt} [nm, cm^{-1}]	$fwhm_{EQE}$ [nm, cm^{-1}]	$fwhm_{opt}$ [nm, cm^{-1}]
	**D4(Pyrl)-A1**	22	84	467, 21 425	464, 21 550	76, 3 500 [b)]	28, 1 275
**H1**	**D4(Pyrl)-A1:D1(Pyrl)-A1 5:5**	18	70	477, 20 975	475, 21 050	76, 3 350 [b)]	25, 1 100
	**D1(Pyrl)-A1**	15	58	487, 20 525	485, 20 625	72, 3 050 [b)]	20, 825
	**D4(Bu)-A2**	13	63	579, 17 275	575, 17 400	56, 1 675 [b)]	61, 1 850
**H2**	**D4(Bu)-A2:D1(Bu)-A2 3:7**	12	57	605, 16 525	592, 16 900	68, 1 875 [b)]	74, 2 150
	**D1(Bu)-A2**	17	88	650, 15 375	629, 15 900	76, 1 800	89, 2 275
	**D4(Hex)-A1**	11	65	708, 14 125	706, 14 150	19, 375	23, 475
**J**	**D4(Hex)-A1:D1(Hex)-A1 7:3**	6	36	733, 13 650	733, 13 650	27, 500	34, 650
	**D1(Hex)-A1**	3	21	763, 13 100	760, 13 150	12, 200	20, 350

[a)] Device architecture: Glass/ITO/PEDOT:PSS/Merocyanine(s)/20 nm $\mathbf{C_{60}}$/5 nm BCP/1 nm Liq/100 nm Al.
[b)] These values were determined as twice the half-width-at-half-maximum value towards higher wavelengths due to a significant spectral overlap with the photoresponse function of electron acceptor C60 and are thus overestimated.

12 General Synthetic Schemes

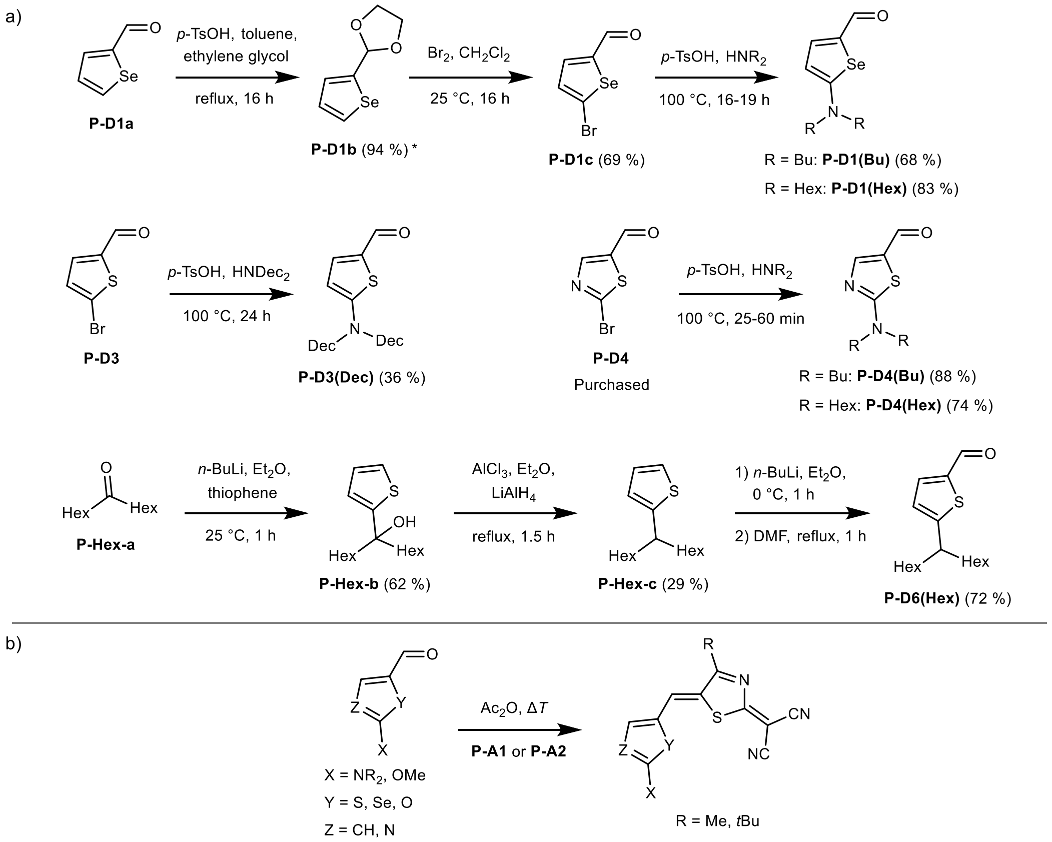

Figure S33. a) Synthetic schemes for literature unknown precursor donor molecules. b) General synthetic procedure for the Knoevenagel condensation step for the formation of the final merocyanine compounds. * This yield of **P-D1b** corresponds to that of the crude product which still includes roughly 30 % of **P-D3a** according to the ^{1}H NMR spectrum shown in Figure S34.

13 Precursor Syntheses and Characterization

All following precursor syntheses and their according characterizations are described in the sequence listed in Table S2.

2-(Selenophen-2-yl)-1,3-dioxolane – P-D1b:

Compound **P-D1a** (selenophene-2-carbaldehyde, 500 mg, 3.14 mmol, 1.00 equiv.) was suspended in 11 mL of toluene. Ethylene glycol (683 mg, 11.00 mmol, 3.50 equiv.) and *p*-toluenesulfonic acid (12 mg, *p*-TsOH, 0.07 mmol, 0.02 equiv.) were added and the reaction mixture stirred under reflux conditions for 16 h with a Dean-Stark trap. The reaction mixture was then extracted with H_2O and ethyl acetate. The organic phase was washed with brine, dried with $MgSO_4$, the solvent removed under vacuum, and the residue further purified using silica-gel column chromatography with CH_2Cl_2. As shown by mass spectrometry and ^{1}H NMR data, even after column chromatography a large amount of reactant **P-D1a** is still present in the product mixture (presumably due to the reversible nature of this reaction type) and a full separation of these two compounds was not possible and also not required, as **P-D1b** is only used as an intermediate for the synthesis of compound **P-D1c**.

Yield: 599 mg (= 94 %) of a dark red liquid; according to the ^{1}H NMR data shown in Figure S34 this corresponds to a yield of **P-D1b** of roughly 427 mg (67 %).

Mass spectrometry (DIP): $[M+H]^+$ found at 204.97698. Calculated for $C_7H_9O_2Se^+$ at 204.97622; large traces of reactant **P-D1a** visible at 160.95157 (calculated for $C_5H_5OSe^+$ at 160.95001). ^{1}H NMR (400 MHz, CD_2Cl_2, 295 K): δ / ppm = 8.03–8.05 (m, 1H), 7.34–7.35 (m, 1H), 7.21–7.26 (m, 1H), 6.02 (s, 1H), 3.95–4.14 (m, 4H).

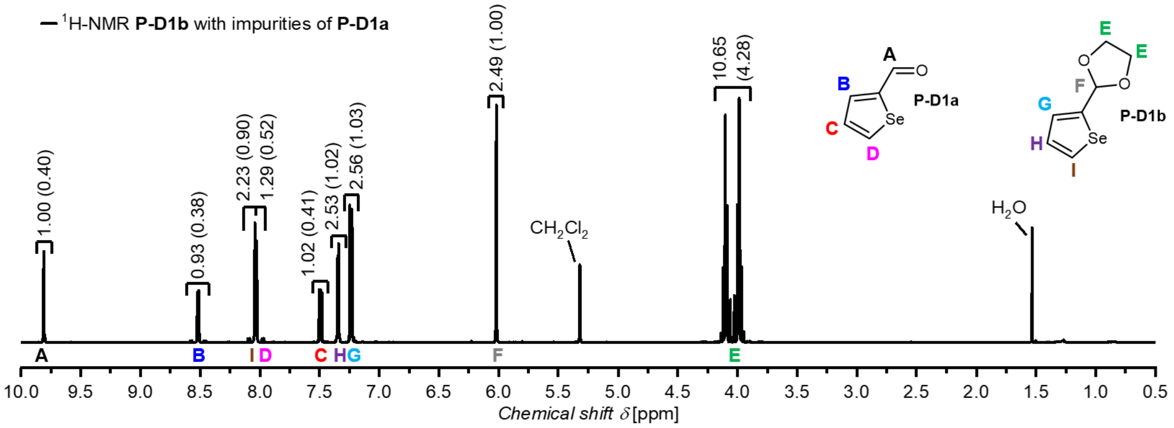

Figure S34. ^{1}H NMR of **P-D1b** after column chromatography measured in CD_2Cl_2 at 295 K and 400 MHz. As described in the synthesis procedure, there are large amounts of the reactant **P-D1a** present due to the reversible nature of this reaction. The ^{1}H NMR data indicates a ratio of 2.5:1.0 of **P-D1b**:**PD1a**. The first integral values are scaled to proton **A** of compound **P-D1a**; those in brackets are scaled to proton **F** of compound **P-D1b**.

5-Bromoselenophene-2-carbaldehyde – P-D1c:

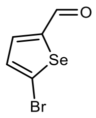

P-D1c

Compound **P-D1b** (1 000 mg, 4.92 mmol, 1.0 equiv.) was suspended in 10 mL CH_2Cl_2 and Br_2 (788 mg, 4.92 mmol, 1.0 equiv.) was added. The reaction was stirred at room temperature for 16 h. The reaction mixture was then diluted with 20 mL of an aqueous Na_2SO_3 solution and extracted using CH_2Cl_2 and H_2O. The organic phase was dried with $MgSO_4$, the solvent was removed under vacuum, and the residue further purified using silica-gel column chromatography with CH_2Cl_2:cyclohexane 3:2. The characterization of **P-D1c** was in accordance with literature data, where it was previously reported using a different synthetic approach.[S5]

Yield: 803 mg (= 69 %) of a brown oil.

7-(Thiophen-2-yl)tridecan-7-ol – P-Hex-b:

Thiophene (2.13 g, 25.3 mmol, 1.00 equiv.) was added to 25 mL anhydrous diethyl ether at 0 °C under nitrogen atmosphere. *n*-Butyllithium (1.86 g, 29.1 mmol, 1.15 equiv.) was added dropwise and, after complete addition, the mixture was heated to reflux for 1 h. After cooling to 0 °C, **P-Hex-a** (tridecan-7-one, 5.77 g, 29.1 mmol, 1.15 equiv.) was added and the mixture stirred for 1 h at room temperature. After addition of H_2O at 0 °C, the reaction mixture was extracted using diethyl ether and H_2O. The organic phase was dried with $MgSO_4$ and filtered off with diethyl ether. The solvent was removed under vacuum and the residue purified by silica-gel column chromatography using a gradient from CH_2Cl_2:cyclohexane 3:1 to CH_2Cl_2. This synthesis protocol was adapted from ref. S53.

Yield: 4.46 g (= 62 %) of a light green oil.

Mass spectrometry (DIP): $[M{-}OH]^+$ found at 265.20099. Calculated for $C_{17}H_{29}S^+$ at 265.19845. ^1H NMR (400 MHz, $CDCl_3$, 295 K): δ / ppm = 7.18 (dd, J = 5.0 Hz, J = 1.2 Hz, 1H), 6.95 (dd, J = 5.0 Hz, J = 3.5 Hz, 1H), 6.85 (dd, J = 3.5 Hz, J = 1.2 Hz, 1H), 1.95 (s, 1H), 1.80–1.84 (m, 4H), 1.17–1.34 (m, 16H), 0.84–0.87 (t, J = 6.9 Hz, 6H). ^{13}C NMR (101 MHz, $CDCl_3$, 295 K): δ / ppm = 152.6, 126.7, 123.6, 122.5, 76.7, 43.3, 31.9, 29.7, 23.6, 22.7, 14.2 (11 carbon signals in total).

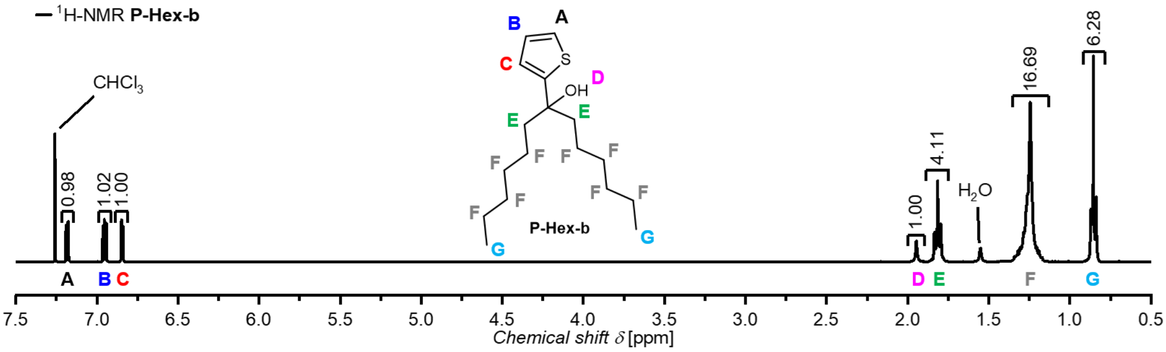

Figure S35. ^1H NMR of compound **P-Hex-b** measured in $CDCl_3$ at 295 K and 400 MHz. All peaks were assigned using an additional ^1H ^1H COSY NMR measurement.

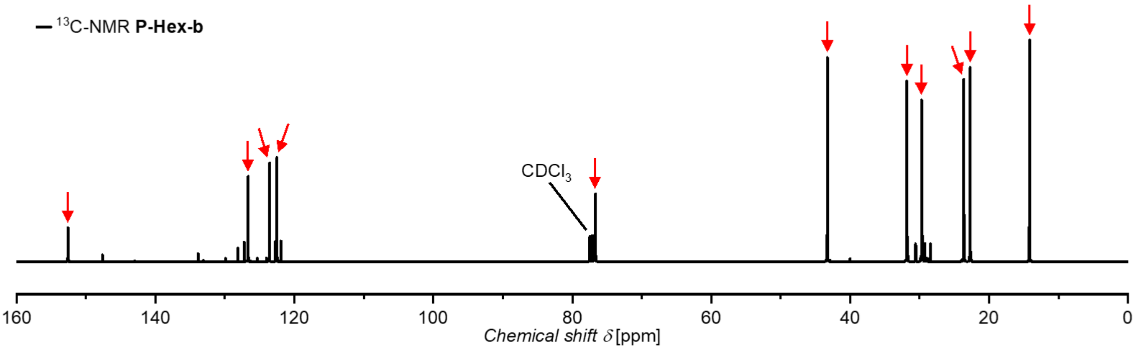

Figure S36. ^{13}C NMR of compound **P-Hex-b** measured in $CDCl_3$ at 295 K and 101 MHz. All peaks belonging to **P-Hex-b** were assigned using an additional ^1H ^{13}C HSQC NMR measurement.

2-(Tridecan-7-yl)thiophene – P-Hex-c:

Under nitrogen atmosphere, $LiAlH_4$ (1.20 g, 31.6 mmol, 2.0 equiv.) was added to a solution of $AlCl_3$ (8.42 g, 63.2 mmol, 4.0 equiv.) in 20 mL of anhydrous diethyl ether. **P-Hex-b** (4.46 g, 15.8 mmol, 1.0 equiv.) was dissolved in 25 mL anhydrous diethyl ether under nitrogen atmosphere and added dropwise to the mixture. The reaction was heated to reflux for 1.5 h, cooled to 0 °C, and quenched by addition of H_2O. The mixture was extracted using diethyl ether and H_2O. The organic phase was dried with $MgSO_4$ and filtered off with diethyl ether. The solvent was removed under vacuum and the residue further purified by flash silica-gel column chromatography using *n*-hexane.

Yield: 1.20 g (= 29 %) of a colorless oil.

Mass spectrometry (DIP): $[M+H]^+$ found at 267.21525. Calculated for $C_{17}H_{31}S^+$ at 267.21410. ^1H NMR (400 MHz, $CDCl_3$, 295 K): δ / ppm = 7.11–7.13 (ddd, J = 5.1 Hz, J = 1.1 Hz, J = 0.5 Hz, 1H), 6.91 (dd, J = 5.1 Hz, J = 3.4 Hz, 1H), 6.74–6.75 (ddd, J = 3.4 Hz, J = 1.1 Hz, J = 0.5 Hz, 1H), 2.78–2.85 (m, 1H), 1.49–1.69 (m, 4H), 1.16–1.31 (m, 16H), 0.84–0.88 (m, 6H). ^{13}C NMR (101 MHz, $CDCl_3$, 295 K): δ / ppm = 150.9, 126.4, 123.5, 122.5, 41.5, 38.2, 31.9, 29.4, 27.5, 22.8, 14.2 (11 carbon signals in total).

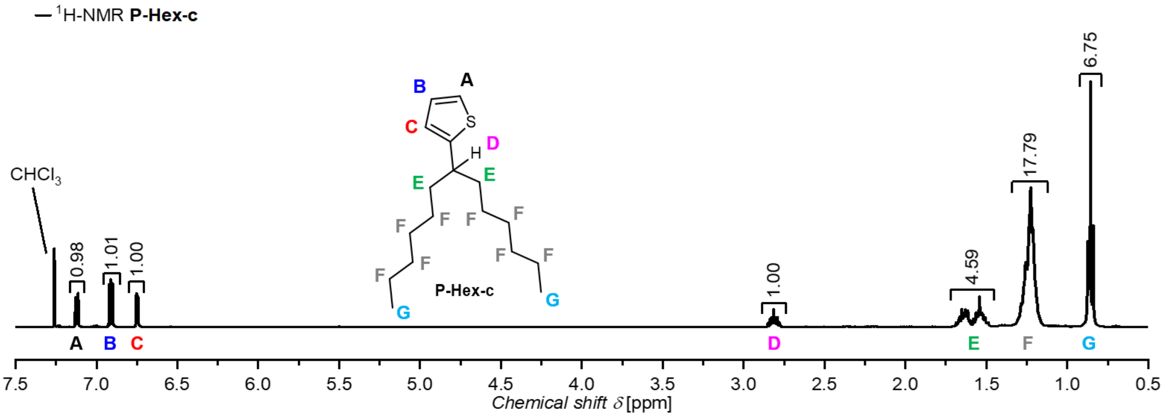

Figure S37. ^1H NMR of compound **P-Hex-c** measured in $CDCl_3$ at 295 K and 400 MHz. All peaks were assigned using an additional ^1H ^1H COSY NMR measurement.

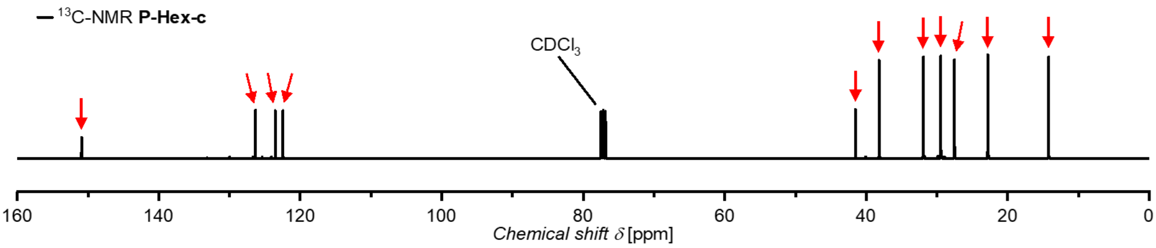

Figure S38. ^{13}C NMR of compound **P-Hex-c** measured in $CDCl_3$ at 295 K and 101 MHz. All peaks belonging to P-Hex-b were assigned using an additional ^1H ^{13}C HSQC NMR measurement.

5-(Dibutylamino)selenophene-2-carbaldehyde – P-D1(Bu):

Compounds **P-D1c** (5-bromoselenophene-2-carbaldehyde, 300 mg, 1.26 mmol, 1.00 equiv.), dihexylamine (489 mg, 3.78 mmol, 3.00 equiv.) and *p*-toluenesulfonic acid (4.79 mg, *p*-TsOH, 0.03 mmol, 0.02 equiv.) were heated to 100 °C for 16 h. The reaction mixture was dried under vacuum and purified using silica-gel column chromatography with CH_2Cl_2:methanol 95:5.

Yield: 244 mg (= 68 %) of a brown oil.

Mass spectrometry (DIP): $[M+H]^+$ found at 288.08645. Calculated for $C_{13}H_{22}NOSe^+$ at 288.08611. ^1H NMR (400 MHz, CD_2Cl_2, 295 K): δ / ppm = 9.37 (s, 1H), 7.64 (d, J = 4.8 Hz, 1H), 5.92 (d, J = 4.8 Hz, 1H), 3.33 (t, J = 7.7 Hz, 4H), 1.63-1.71 (m, 4H), 1.37 (m, 4H), 0.96 (t, J = 7.4 Hz, 6H). ^{13}C NMR (101 MHz, $CDCl_3$, 295 K): δ / ppm = 181.0, 171.4, 143.8, 129.2, 103.8, 55.1, 29.1, 20.3, 13.9 (9 carbon signals in total).

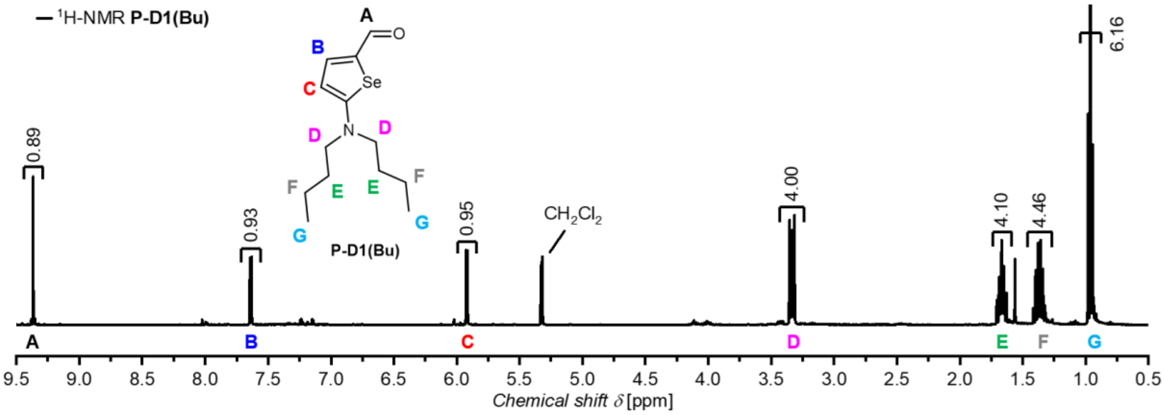

Figure S39. ^1H NMR of compound **P-D1(Bu)** measured in CD_2Cl_2 at 295 K and 400 MHz.

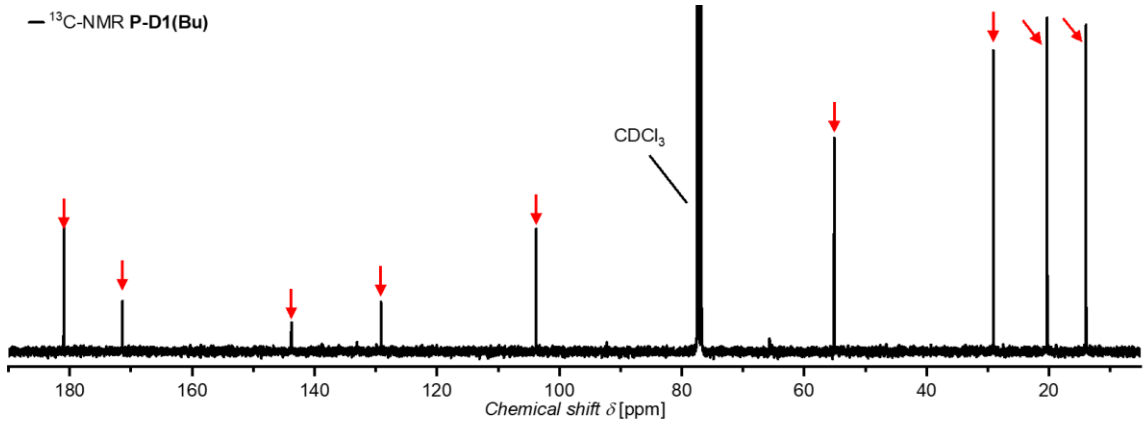

Figure S40. ^{13}C NMR of compound **P-D1(Bu)** measured in $CDCl_3$ at 295 K and 101 MHz.

5-(Dihexylamino)selenophene-2-carbaldehyde – P-D1(Hex):

Compounds **P-D1c** (5-bromoselenophene-2-carbaldehyde, 100 mg, 0.42 mmol, 1.00 equiv.), dihexylamine (234 mg, 1.26 mmol, 3.00 equiv.) and *p*-toluenesulfonic acid (1.45 mg, *p*-TsOH, 0.01 mmol, 0.02 equiv.) were heated to 100 °C for 19 h. The reaction mixture was dried under vacuum and purified using silica-gel column chromatography with CH_2Cl_2.

Yield: 119 mg (= 83 %) of a brown oil.

Mass spectrometry (DIP): $[M+H]^+$ found at 344.14939. Calculated for $C_{17}H_{30}NOSe^+$ at 344.14871. ^{1}H NMR (400 MHz, $CDCl_3$, 295 K): δ / ppm = 9.41 (s, 1H), 7.64 (d, J = 4.8 Hz, 1H), 5.89 (d, J = 4.8 Hz, 1H), 3.31 (t, J = 7.8 Hz, 4H), 1.67 (m, 4H), 1.25–1.36 (m, 12H), 0.90 (t, J = 6.7 Hz, 6H). ^{13}C NMR (101 MHz, $CDCl_3$, 295 K): δ / ppm = 181.0, 171.4, 143.8, 129.2, 103.8, 55.4, 31.6, 27.0, 26.7, 22.7, 14.1 (11 carbon signals in total).

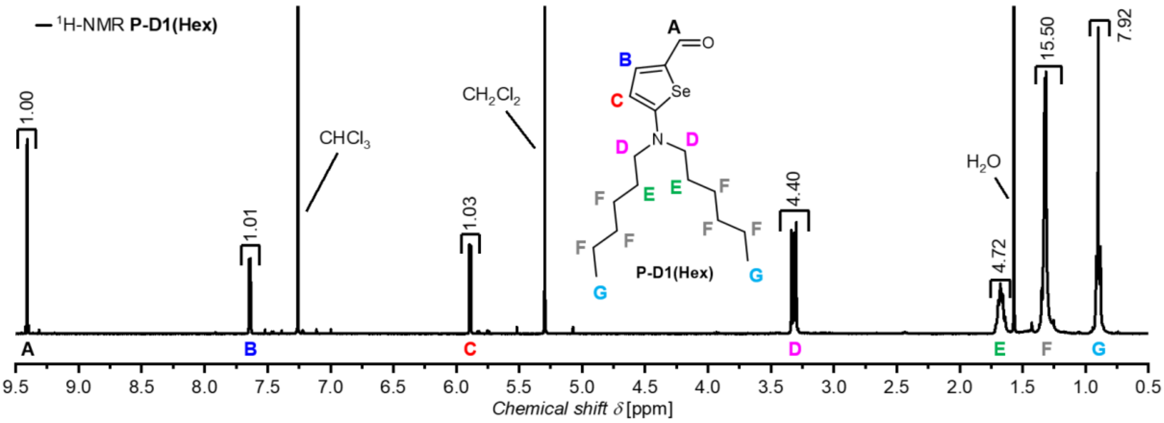

Figure S41. ^{1}H NMR of compound **P-D1(Hex)** measured in $CDCl_3$ at 295 K and 400 MHz.

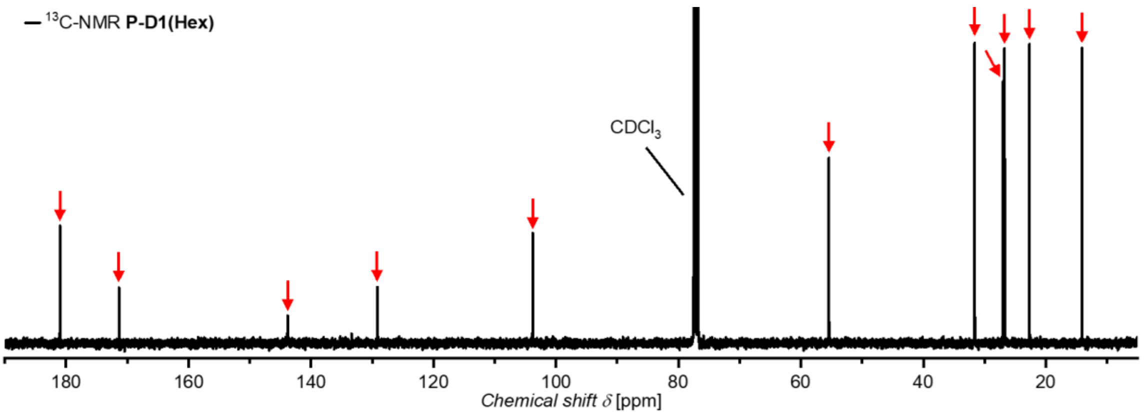

Figure S42. ^{13}C NMR of compound **P-D1(Hex)** measured in $CDCl_3$ at 295 K and 101 MHz.

5-(Didecylamino)thiophene-2-carbaldehyde – P-D3(Oct):

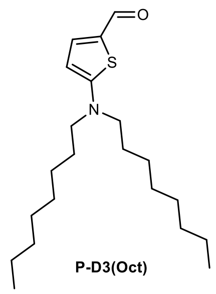

Compounds **P-D3** (5-bromothiophene-2-carbaldehyde, 0.50 g, 2.62 mmol, 1.00 equiv.), dioctylamine (1.90 g, 7.85 mmol, 3.00 equiv.) and *p*-toluenesulfonic acid (13.5 mg, *p*-TsOH, 0.08 mmol, 0.03 equiv.) were heated to 100 °C for 18.5 h. The reaction mixture was then precipitated by addition of 5 mL toluene. The precipitate was collected by filtration, washed with toluene, and dried under vacuum. The residue was purified using silica-gel column chromatography with toluene:ethyl acetate 30:1. **P-D3(Oct)** was characterized according to literature, where it was previously synthesized without indication of the synthetic yield.[S54]

Yield: 314 mg (= 34 %) of a brown oil.

5-(Didecylamino)thiophene-2-carbaldehyde – P-D3(Dec):

Compounds **P-D3** (5-bromothiophene-2-carbaldehyde, 1.00 g, 5.23 mmol, 1.00 equiv.), didecylamine (4.67 g, 15.70 mmol, 3.00 equiv.) and *p*-toluenesulfonic acid (55.0 mg, *p*-TsOH, 0.32 mmol, 0.06 equiv.) were heated to 100 °C for 10 h under reflux conditions. The reaction mixture was then extracted with CH_2Cl_2 and H_2O. The organic phase dried with $MgSO_4$ and filtered off with *n*-hexane and isopropanol. The solvent was removed under vacuum and the residue purified using flash silica-gel column chromatography with a gradient from cyclohexane to cyclohexane:ethyl acetate 97:3.

Yield: 765 mg (= 36 %) of a brown oil.

Mass spectrometry (MALDI-TOF): $[M]^+$ found at 407.32750. Calculated for $C_{25}H_{45}NOS^+$ at 407.32164. ^{1}H NMR (400 MHz, $CDCl_3$, 295 K): δ / ppm = 9.46 (s, 1H), 7.44 (d, J = 4.5 Hz, 1H), 5.89 (d, J = 4.5 Hz, 1H), 3.32 (t, J = 7.7 Hz, 4H), 1.61–1.69 (m, 4H), 1.23–1.34 (m, 28H), 0.88 (t, J = 6.8 Hz, 6H). ^{13}C NMR (101 MHz, $CDCl_3$, 295 K): δ / ppm = 179.8, 167.1, 140.8, 125.3, 102.8, 53.9, 32.0, 29.67, 29.63, 29.47, 29.40, 27.05, 26.99, 22.8, 14.2 (15 carbon signals in total).

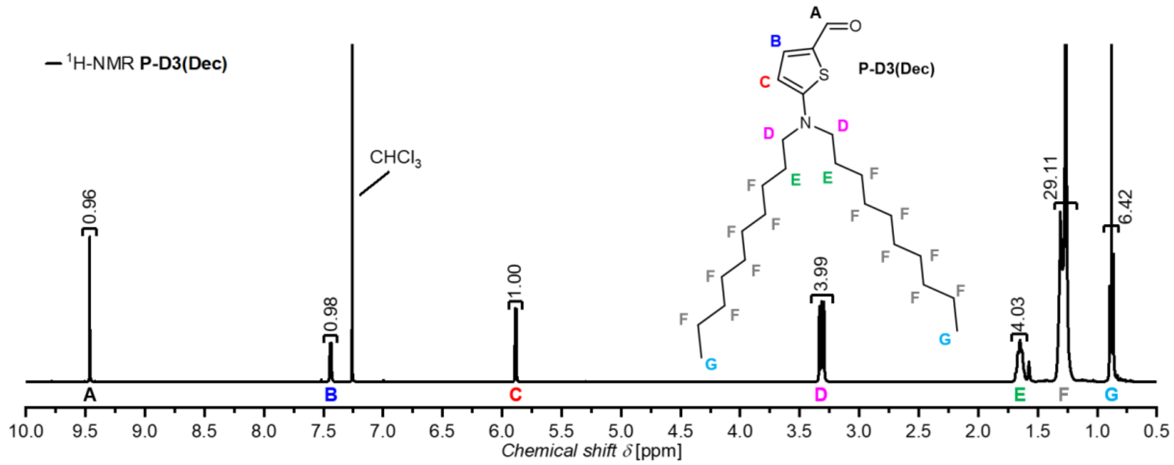

Figure S43. ^{1}H NMR of compound **P-D3(Dec)** measured in $CDCl_3$ at 295 K and 400 MHz.

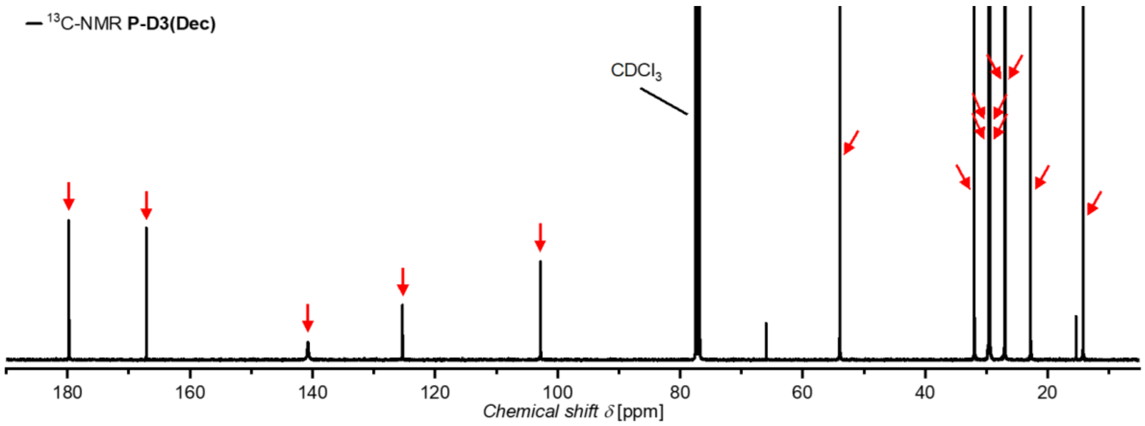

Figure S44. ^{13}C NMR of compound **P-D3(Dec)** measured in $CDCl_3$ at 295 K and 101 MHz. The two non-assigned peaks were identified as impurities using an additional ^{1}H ^{13}C HSQC NMR measurement.

2-(Dibutylamino)thiazole-5-carbaldehyde – P-D4(Bu):

Compounds **P-D4** (5-bromothiazole-2-carbaldehyde, 1.00 g, 5.21 mmol, 1.00 equiv.), dibutylamine (2.02 g, 15.62 mmol, 3.00 equiv.) and *p*-toluenesulfonic acid (55.0 mg, *p*-TsOH, 0.32 mmol, 0.06 equiv.) were heated to 100 °C for 25 min. The reaction mixture was extracted with CH_2Cl_2 and H_2O. The organic phase was dried with $MgSO_4$ and filtered off with *n*-hexane and isopropanol. The solvent was removed under vacuum and the residue purified using flash silica-gel column chromatography with a gradient from cyclohexane to cyclohexane:ethyl acetate 97:3.

Yield: 1.1 g (= 88 %) of a brown oil.

Mass spectrometry (MALDI-TOF): $[M+H]^+$ found at 241.13678. Calculated for $C_{12}H_{21}N_2OS^+$ at 241.13691. ^{1}H NMR (400 MHz, $CDCl_3$, 295 K): δ / ppm = 9.66 (s, 1H), 7.84 (s, 1H), 3.49 (m, 4H), 1.62–1.70 (m, 4H), 1.37 (sext, J = 7.5 Hz, 4H), 0.96 (t, J = 7.5 Hz, 6H). ^{13}C NMR (101 MHz, $CDCl_3$, 295 K): δ / ppm = 180.6, 175.4, 154.1, 128.0, 52.0, 29.2, 20.2, 13.9 (8 carbon signals in total).

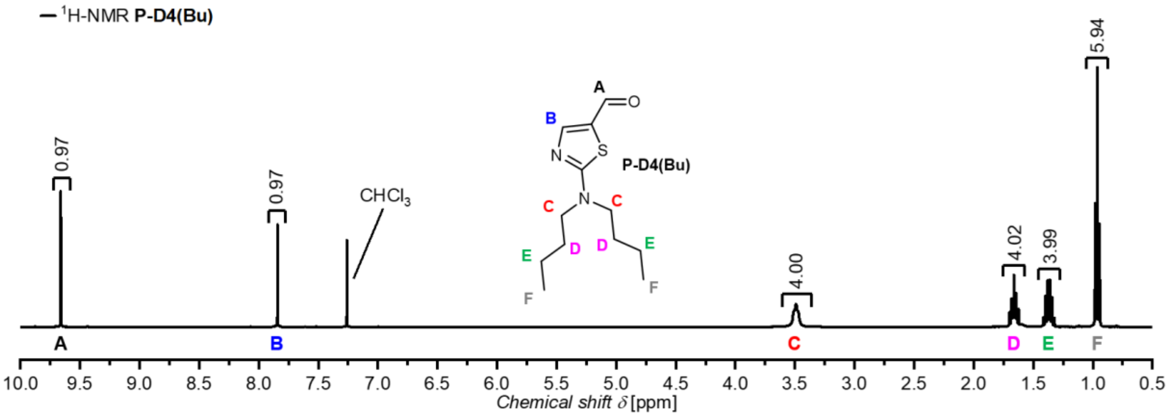

Figure S45. ^{1}H NMR of compound **P-D4(Bu)** measured in $CDCl_3$ at 295 K and 400 MHz.

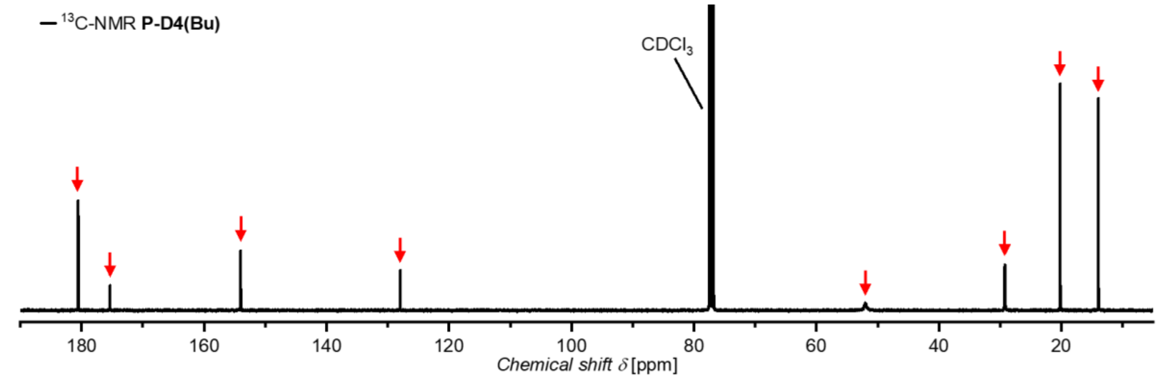

Figure S46. ^{13}C NMR of compound **P-D4(Bu)** measured in $CDCl_3$ at 295 K and 101 MHz.

2-(Dihexylamino)thiazole-5-carbaldehyde – P-D4(Hex):

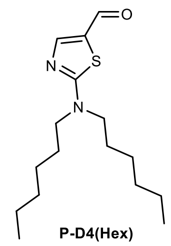

P-D4(Hex)

Compounds **P-D4** (5-bromothiazole-2-carbaldehyde, 2.00 g, 10.42 mmol, 1.00 equiv.), dihexylamine (5.79 g, 31.25 mmol, 3.00 equiv.) and *p*-toluenesulfonic acid (110 mg, *p*-TsOH, 0.64 mmol, 0.06 equiv.) were heated to 100 °C for 60 min. The reaction mixture was extracted using CH_2Cl_2 and H_2O. The organic phase was dried with $MgSO_4$ and filtered off with CH_2Cl_2. The solvent was removed under vacuum and the residue purified using flash silica-gel column chromatography with a gradient of cyclohexane to cyclohexane:ethyl acetate 97:3. **P-D4(Hex)** was characterized according to literature, where it was previously synthesized according to a different synthetic route.[S55]

Yield: 2.3 g (= 74 %) of a red oil.

5-(Tridecan-7-yl)thiophene-2-carbaldehyde – P-D6(Hex):

Under nitrogen atmosphere, **P-Hex-c** (600 mg, 2.25 mmol, 1.0 equiv.) was dissolved in 3 mL anhydrous diethyl ether and cooled to 0 °C. After addition of *n*-butyllithium (159 mg, 2.48 mmol, 1.1 equiv.), the reaction mixture was kept at 0 °C for 1 h and then stirred at room temperature for 15 min. Dimethylformamide (165 mg, 2.25 mmol, 1.0 equiv.) was added and the mixture heated to reflux for 1 h. After cooling to room temperature, the reaction was quenched with 200 mL of H_2O and extracted using ethyl acetate and H_2O. The crude product was purified by flash silica-gel column chromatography using a gradient from cyclohexane to CH_2Cl_2:cyclohexane 3:7.

Yield: 480 mg (= 72 %) of a light yellow oil.

Mass spectrometry (DIP): $[M+H]^+$ found at 295.2100. Calculated for $C_{18}H_{31}OS^+$ at 295.20901. ^1H NMR (400 MHz, $CDCl_3$, 295 K): δ / ppm = 9.83 (s, 1H), 7.61 (d, J = 3.8 Hz, 1H), 6.88 (d, J = 3.8 Hz, 1H), 2.82–2.90 (m, 1H), 1.51–1.73 (m, 4H), 1.11–1.33 (m, 16H), 0.82–0.91 (t, J = 7.0 Hz, 6H). ^{13}C NMR (101 MHz, $CDCl_3$, 295 K): δ / ppm = 182.7, 163.0, 141.4, 136.9, 125.4, 42.5, 37.9, 31.8, 29.3, 27.4, 22.7, 14.1 (12 carbon signals in total).

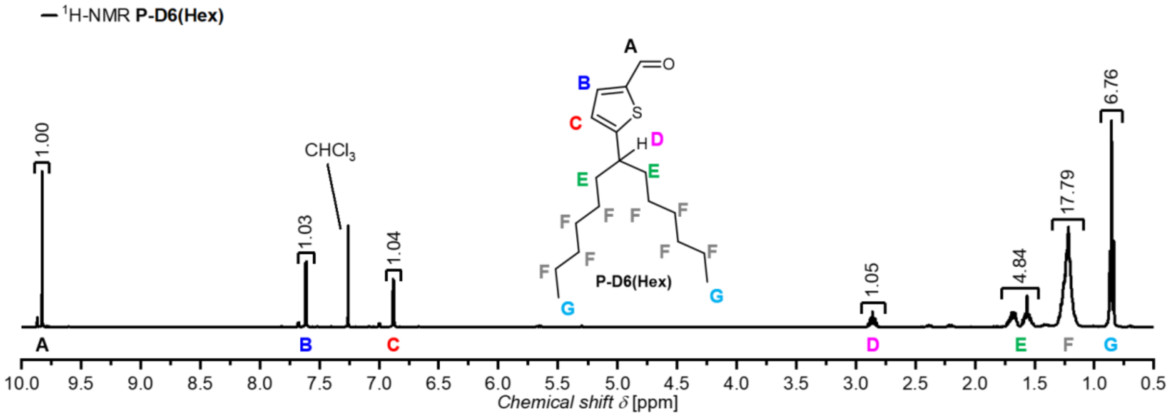

Figure S47. ^1H NMR of compound **P-D6(Hex)** measured in $CDCl_3$ at 295 K and 400 MHz.

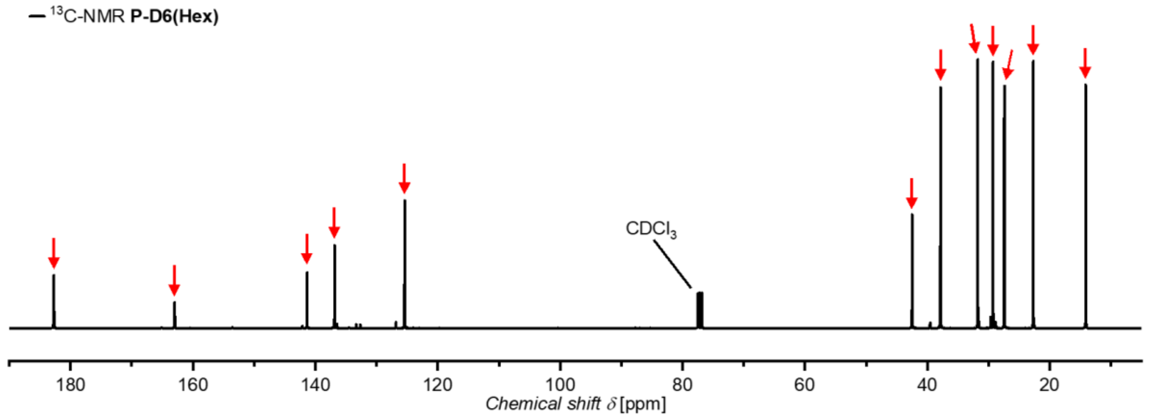

Figure S48. ^{13}C NMR of compound **P-D6(Hex)** measured in $CDCl_3$ at 295 K and 101 MHz.

14 Merocyanine Syntheses and Characterization

All following merocyanine syntheses and their according characterizations are described in the sequence listed in Table S1.

(*Z*)-2-(4-(*tert*-Butyl)-5-((5-(dihexylamino)selenophen-2-yl)methylene)thiazol-2(5*H*)-ylidene)malononitrile – D1(Hex)-A1:

A solution of **P-D1(Hex)** (2-(dihexylamino)selenophene-5-carbaldehyde, 99 mg, 0.29 mmol, 1.0 equiv.) and **P-A1** (2-[4-(*tert*-butyl)thiazol-2(3*H*)-ylidene]malononitrile, 59 mg, 0.29 mmol, 1.0 equiv.) in 0.7 mL Ac_2O was heated to 90 °C for 60 min. The reaction mixture was extracted using CH_2Cl_2 and H_2O and the crude product was purified by silica-gel column chromatography using CH_2Cl_2:ethyl acetate 90:10 and precipitation from CH_2Cl_2 and methanol. The precipitate was collected by filtration, washed with methanol and *n*-hexane, and dried under vacuum.

Yield: 141 mg (= 92 %) of a deep blue powder.

Melting point: ~ 155 °C. Mass spectrometry (MALDI-TOF): $[M]^+$ found at 530.28053. Calculated for $C_{27}H_{38}N_4SSe^+$ at 530.19769. ^{1}H NMR (400 MHz, $CDCl_3$, 295 K): δ / ppm = 8.08 (s, 1H), 7.55 (d, J = 5.2 Hz, 1H), 6.19 (d, J = 5.2 Hz, 1H), 3.47 (t, J = 7.8 Hz, 4H), 1.75 (m, 4H), 1.51 (s, 9H), 1.30–1.43 (m, 12H), 0.92 (t, J = 7.0 Hz, 6H). ^{13}C NMR (101 MHz, $CDCl_3$, 295 K): δ / ppm = 184.9, 179.5, 177.8, 148.9, 134.6, 127.3, 123.7, 118.3, 115.9, 110.0, 56.6, 38.2, 31.60, 31.57, 27.4, 26.7, 22.6, 14.1 (18 carbon signals in total). UV/Vis ($CHCl_3$): λ_{00} [nm] (ε [M^{-1} cm^{-1}]) = 662 (120 000).

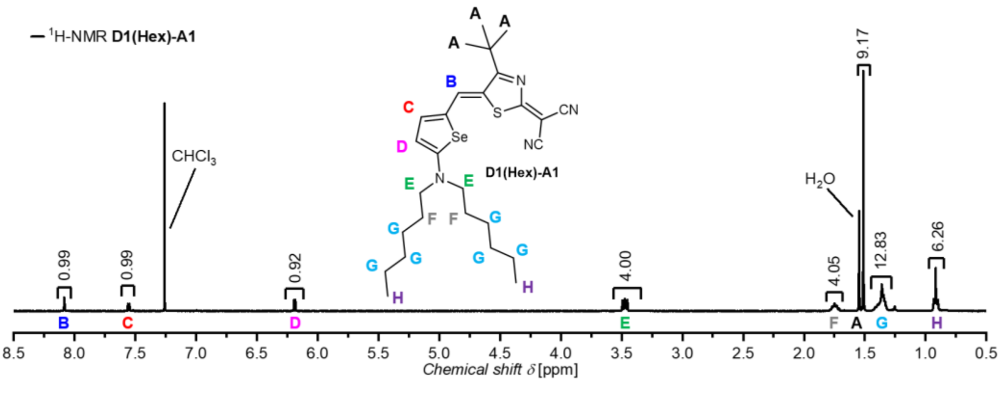

Figure S49. ^{1}H NMR of compound **D1(Hex)-A1** measured in $CDCl_3$ at 295 K and 400 MHz.

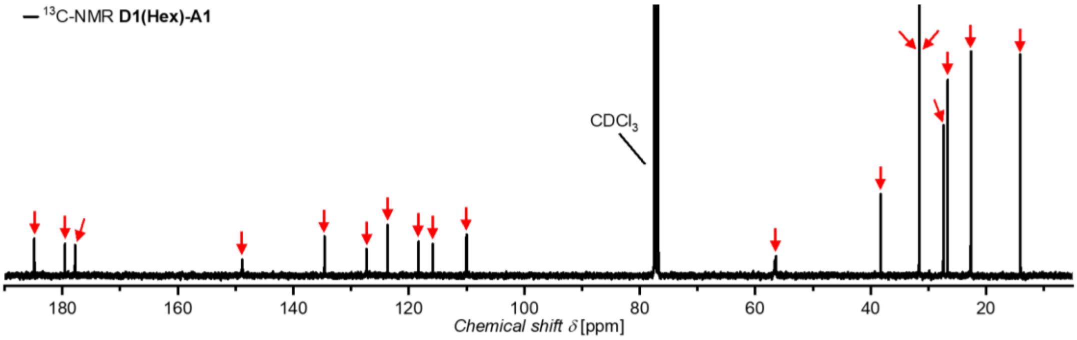

Figure S50. ^{13}C NMR of compound **D1(Hex)-A1** measured in $CDCl_3$ at 295 K and 101 MHz.

(Z)-2-(4-(*tert*-Butyl)-5-((5-(pyrrolidin-1-yl)selenophen-2-yl)methylene)thiazol-2(5*H*)-ylidene)malononitrile – D1(Pyrl)-A1:

A solution of **P-D1(Pyrl)** (5-(pyrrolidin-1-yl)selenophene-2-carbaldehyde, 25 mg, 0.11 mmol, 1.0 equiv.) and **P-A1** (2-[4-(*tert*-butyl)thiazol-2(3*H*)-ylidene]malononitrile, 22 mg, 0.11 mmol, 1.0 equiv.) in 0.3 mL Ac_2O was heated to 100 °C for 45 min. The reaction mixture was extracted using CH_2Cl_2 and H_2O, and the crude product further purified by silica-gel column chromatography using a gradient from CH_2Cl_2 to CH_2Cl_2:ethyl acetate 90:10 and precipitation from CH_2Cl_2 and methanol. The precipitate was collected by filtration, washed with methanol and *n*-hexane, and dried under vacuum.

Yield: 36 mg (= 78 %) of a dark blue/black powder.

Melting point: ~ 260 °C. Mass spectrometry (MALDI-TOF): $[M]^+$ found at 416.14300. Calculated for $C_{19}H_{20}N_4SSe^+$ at 416.05684. ^{1}H NMR (400 MHz, $CDCl_3$, 295 K): δ / ppm = 8.08 (s, 1H), 7.57 (dd, J = 5.1 Hz, J = 0.5 Hz, 1H), 6.14 (d, J = 5.1 Hz, 1H), 3.54 (t, J = 6.8 Hz, 4H), 2.22 (m, 4H), 1.50 (s, 9H). ^{13}C NMR (101 MHz, $CDCl_3$, 295 K): δ / ppm = 185.1, 179.5, 174.4, 148.2, 134.6, 128.2, 123.9, 118.2, 115.8, 110.0, 53.3, 39.3, 32.6, 26.1 (14 carbon signals in total). UV/Vis ($CHCl_3$): λ_{00} [nm] (ε [M^{-1} cm^{-1}]) = 659 (119 000).

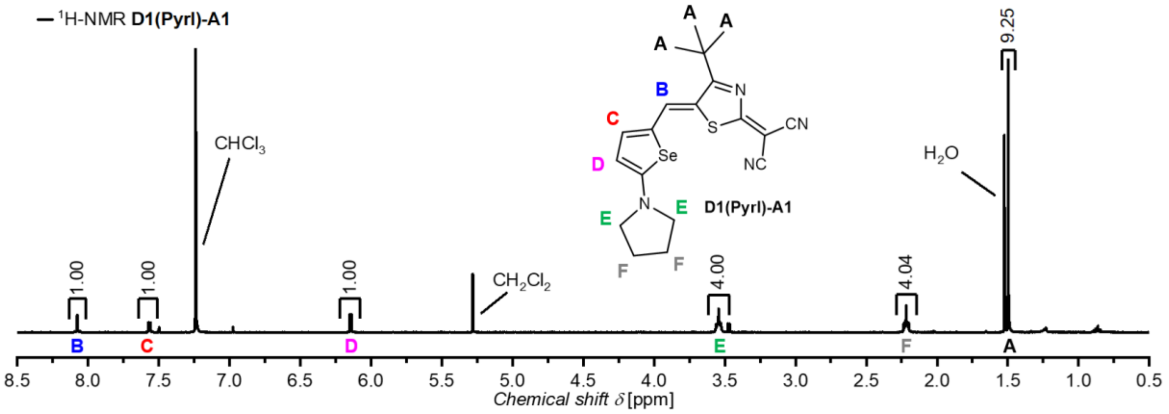

Figure S51. ^{1}H NMR of compound **D1(Pyrl)-A1** measured in $CDCl_3$ at 295 K and 400 MHz.

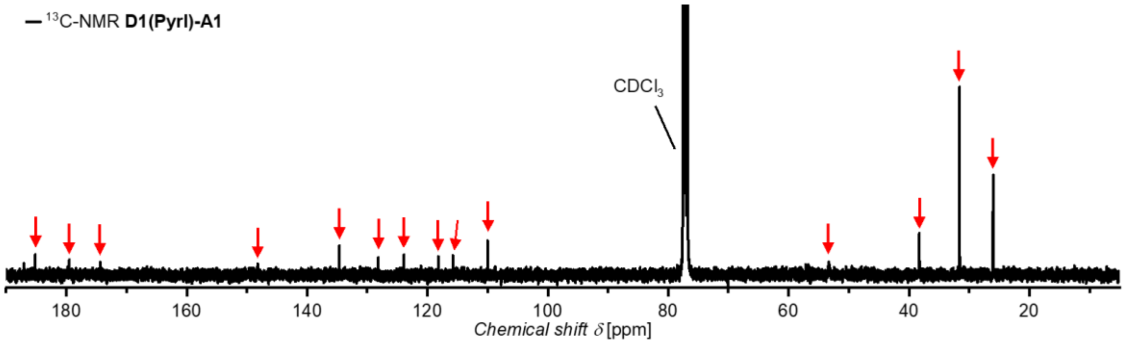

Figure S52. ^{13}C NMR of compound **D1(Pyrl)-A1** measured in $CDCl_3$ at 295 K and 101 MHz.

(*Z*)-2-(4-(*tert*-Butyl)-5-((5-(pyrrolidin-1-yl)furan-2-yl)methylene)thiazol-2(5*H*)-ylidene)malononitrile – D2(Pyrl)-A1:

A solution of **P-D2(Pyrl)** (5-(pyrrolidin-1-yl)furan-2-carbaldehyde, 200 mg, 1.21 mmol, 1.0 equiv.) and **P-A1** (2-[4-(*tert*-butyl)thiazol-2(3*H*)-ylidene]malononitrile, 249 mg, 1.21 mmol, 1.0 equiv.) in 5.0 mL Ac_2O was heated to 90 °C for 60 min. After cooling to room temperature, 6 ml of *n*-hexane were added and the precipitate was collected by filtration, washed with isopropanol and *n*-hexane, and dried under vacuum. The product was further purified by flash silica-gel column chromatography using a gradient from CH_2Cl_2 to CH_2Cl_2:ethyl acetate 9:1, precipitated from CH_2Cl_2 and ethyl acetate, collected by filtration, and dried under vacuum.

Yield: 335 mg (= 79 %) of a dark green metallic powder.

Melting point: > 300 °C. Mass spectrometry (MALDI-TOF): $[M]^+$ found at 352.19301. Calculated for $C_{19}H_{20}N_4OS^+$ at 352.13523. ^{1}H NMR (400 MHz, dimethyl sulfoxide-d$_6$, 295 K): δ / ppm = 8.01 (d, J = 5.0 Hz, 1H), 7.73 (s, 1H), 6.64 (d, J = 5.0 Hz, 1H), 3.79 (broad, 4H), 2.04–2.08 (m, 4H), 1.43 (s, 9H). UV/Vis ($CHCl_3$): λ_{00} [nm] (ε [M^{-1} cm^{-1}]) = 657 (108 000).

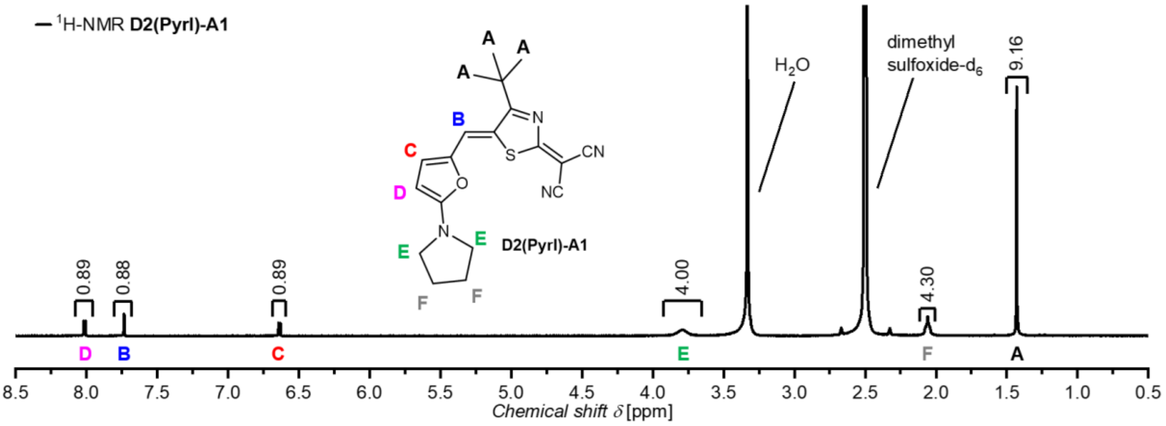

Figure S53. ^{1}H NMR of compound **D2(Pyrl)-A1** measured in dimethyl sulfoxide-d$_6$ at 295 K and 400 MHz.

(*Z*)-2-(4-(*tert*-Butyl)-5-((5-(dioctylamino)thiophen-2-yl)methylene)thiazol-2(5*H*)-ylidene)malononitrile – D3(Oct)-A1:

A solution of **P-D3(Oct)** (5-(dioctylamino)thiophene-2-carbaldehyde, 195 mg, 0.55 mmol, 1.0 equiv.) and **P-A1** (2-[4-(*tert*-butyl)thiazol-2(3*H*)-ylidene]malononitrile, 114 mg, 0.55 mmol, 1.0 equiv.) in 1.4 mL Ac_2O was heated to 90 °C for 60 min. The reaction mixture was extracted with CH_2Cl_2 and water, purified by silica-gel column chromatography using a gradient from CH_2Cl_2 to CH_2Cl_2:ethyl acetate 95:5, precipitated from CH_2Cl_2 and methanol, filtered off with methanol, and successively dried under vacuum.

Yield: 205 mg (= 65 %) of a dark purple solid.

Melting point: ~ 145 °C. Mass spectrometry (MALDI-TOF): $[M+H]^+$ found at 538.38345. Calculated for $C_{31}H_{47}N_4S_2^+$ at 539.32367. ^1H NMR (400 MHz, $CDCl_3$, 295 K): δ / ppm = 7.98 (s, 1H), 7.40 (d, J = 4.7 Hz, 1H), 6.19 (d, J = 4.7 Hz, 1H), 3.48 (t, J = 7.6 Hz, 4H), 1.67–1.76 (m, 4H), 1.51 (s, 9H), 1.24–1.42 (m, 20H), 0.89 (t, J = 6.8 Hz, 6H). ^{13}C NMR (151 MHz, $CDCl_3$, 295 K): δ / ppm = 185.7, 180.2, 171.2, 145.0, 131.6, 123.8, 117.8, 115.5, 108.3, 57.4, 54.6, 38.2, 31.8, 31.5, 29.3, 29.1, 27.3, 26.9, 22.6, 14.1 (20 carbon signals in total). UV/Vis ($CHCl_3$): λ_{00} [nm] (ε [M^{-1} cm^{-1}]) = 654 (122 000).

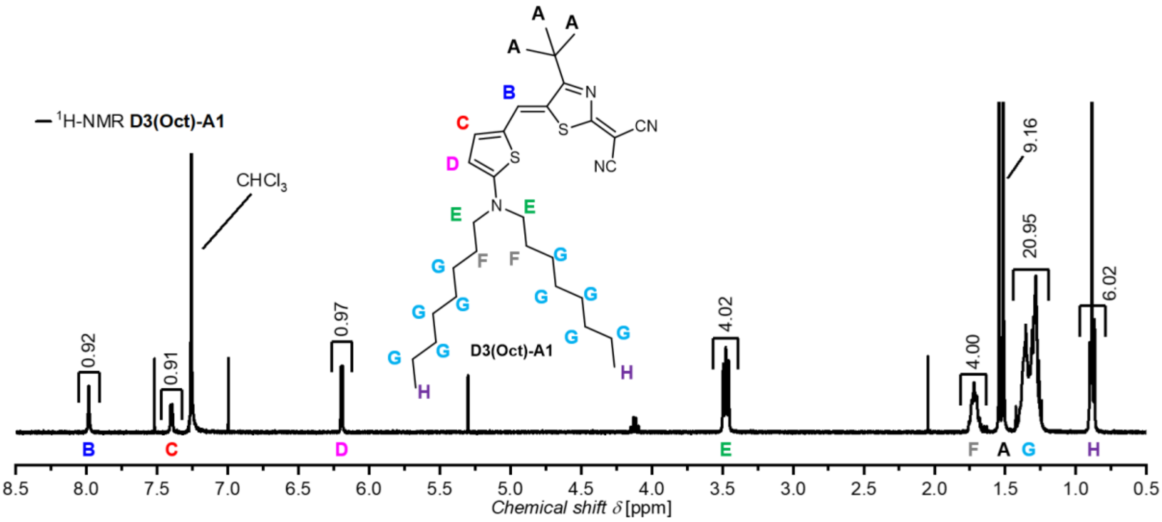

Figure S54. ^1H NMR of compound **D3(Oct)-A1** measured in $CDCl_3$ at 295 K and 400 MHz.

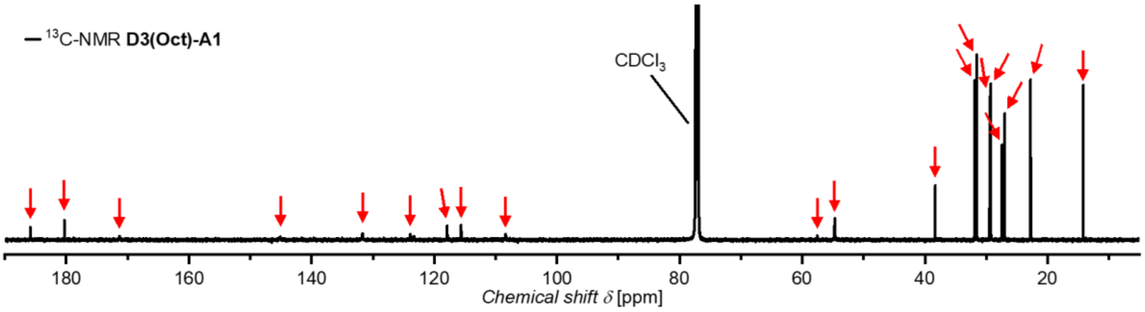

Figure S55. ^{13}C NMR of compound **D3(Oct)-A1** measured in $CDCl_3$ at 295 K and 151 MHz.

(*Z*)-2-(4-(*tert*-Butyl)-5-((5-(didecylamino)thiophen-2-yl)methylene)thiazol-2(5*H*)-ylidene)malononitrile – D3(Dec)-A1:

A solution of **P-D3(Dec)** (5-(didecylamino)thiophene-2-carbaldehyde, 400 mg, 0.92 mmol, 1.0 equiv.) and **P-A1** (2-[4-(*tert*-butyl)thiazol-2(3*H*)-ylidene]malononitrile, 188 mg, 0.92 mmol, 1.0 equiv.) in 4.0 mL Ac_2O was heated to 100 °C for 50 min. The reaction mixture was extracted with CH_2Cl_2 and brine, purified by flash silica-gel column chromatography using a gradient from CH_2Cl_2 to CH_2Cl_2:ethyl acetate 99:1, precipitated from CH_2Cl_2 and methanol, filtered off with methanol and *n*-hexane, and successively dried under vacuum.

Yield: 425 mg (= 74 %) of a dark green solid.

Melting point: ~ 150 °C. Mass spectrometry (MALDI-TOF): $[M]^+$ found at 594.37656. Calculated for $C_{35}H_{54}N_4S_2^+$ at 594.37844. ^1H NMR (400 MHz, $CDCl_3$, 295 K): δ / ppm = 7.99 (s, 1H), 7.40 (d, J = 4.7 Hz, 1H), 6.20 (d, J = 4.7 Hz, 1H), 3.48 (t, J = 7.6 Hz, 4H), 1.67–1.75 (m, 4H), 1.51 (s, 9H), 1.23–1.40 (m, 28H), 0.88 (t, J = 6.8 Hz, 6H). ^{13}C NMR (101 MHz, $CDCl_3$, 295 K): δ / ppm = 185.1, 179.8, 172.0, 145.8, 131.9, 123.4, 118.1, 115.9, 109.2, 55.8, 54.7, 38.1, 32.0, 31.6, 29.60, 29.57, 29.42, 29.38, 27.4, 27.0, 22.8, 14.2 (22 carbon signals in total). UV/Vis ($CHCl_3$): λ_{00} [nm] (ε [M^{-1} cm^{-1}]) = 654 (128 000).

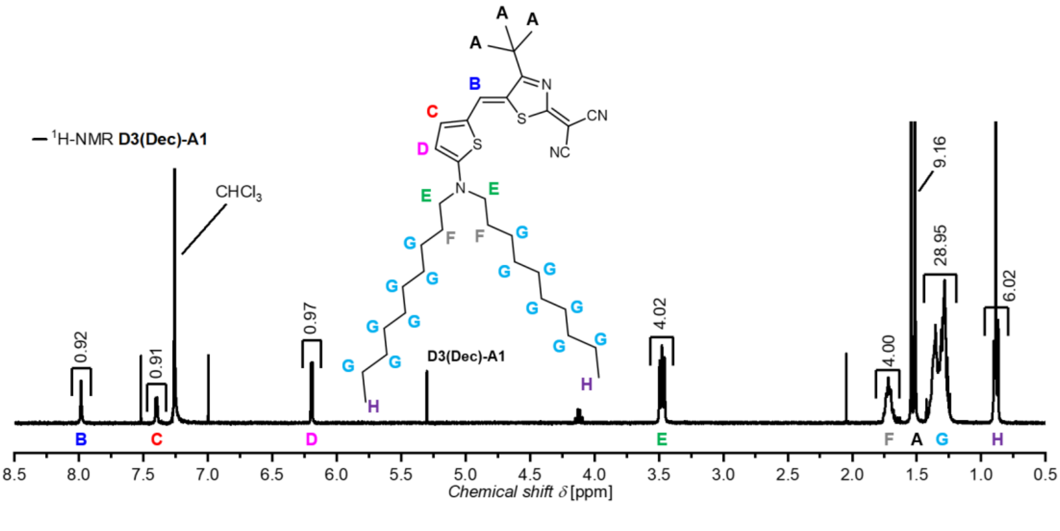

Figure S56. ^1H NMR of compound **D3(Dec)-A1** measured in $CDCl_3$ at 295 K and 400 MHz.

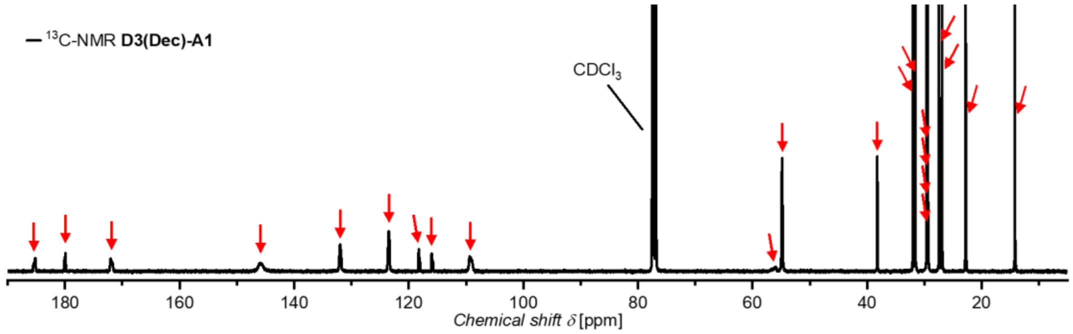

Figure S57. ^{13}C NMR of compound **D3(Dec)-A1** measured in $CDCl_3$ at 295 K and 101 MHz.

(Z)-2-(4-(*tert*-Butyl)-5-((2-(dihexylamino)thiazol-5-yl)methylene)thiazol-2(5H)-ylidene)malononitrile – D4(Hex)-A1:

A solution of **P-D4(Hex)** (2-(dihexylamino)thiazole-5-carbaldehyde, 600 mg, 2.02 mmol, 1.0 equiv.) and **P-A1** (2-[4-(*tert*-butyl)thiazol-2(3H)-ylidene]malononitrile, 415 mg, 2.02 mmol, 1.0 equiv.) in 5.0 mL Ac_2O was heated to 90 °C for 60 min. The green precipitate was filtered off with isopropanol and *n*-hexane, purified by silica-gel column chromatography using CH_2Cl_2, and precipitated from methanol/CH_2Cl_2.

Yield: 419 mg (= 43 %) of a deep red/purple solid.

Melting point: ~ 205 °C. Mass spectrometry (MALDI-TOF): $[M]^+$ found at 483.25252. Calculated for $C_{26}H_{37}N_5S_2^+$ at 483.24849. ^{1}H NMR (400 MHz, $CDCl_3$, 295 K): δ / ppm = 7.99 (s, 1H), 7.73 (s, 1H), 3.56 (s, 4H), 1.69 (m, 4H), 1.52 (s, 9H), 1.33 (m, 12H), 0.89 (t, J = 6.9 Hz, 6H). ^{13}C NMR (101 MHz, $CDCl_3$, 295 K): δ / ppm = 188.5, 180.7, 176.4, 156.9, 129.9, 128.0, 124.0, 116.7, 114.1, 62.0, 38.6, 31.5, 31.2, 27.2, 26.5, 22.5, 14.0 (17 carbon signals in total). UV/Vis ($CHCl_3$): λ_{00} [nm] (ε [M^{-1} cm^{-1}]) = 612 (52 000).

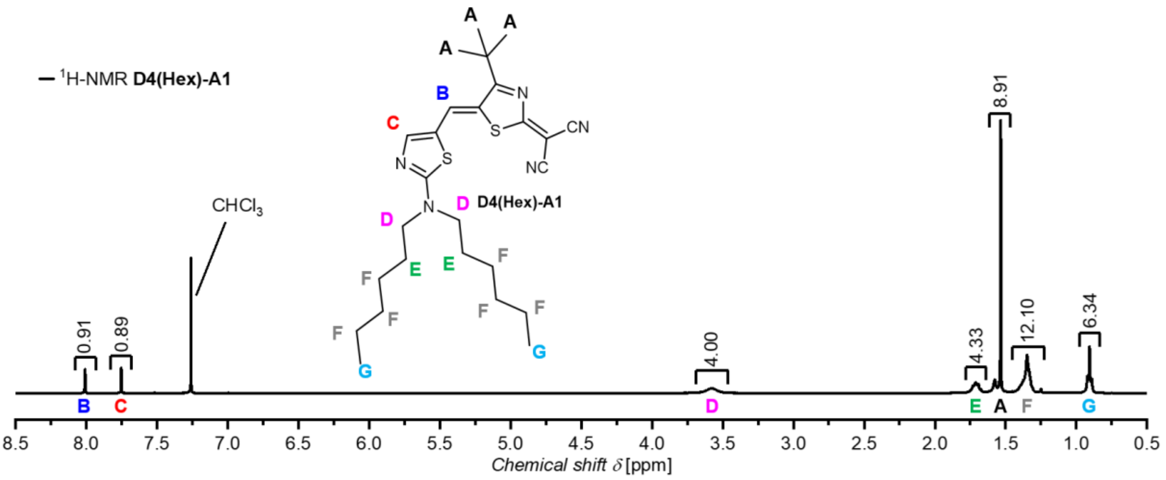

Figure S58. ^{1}H NMR of compound **D4(Hex)-A1** measured in $CDCl_3$ at 295 K and 400 MHz.

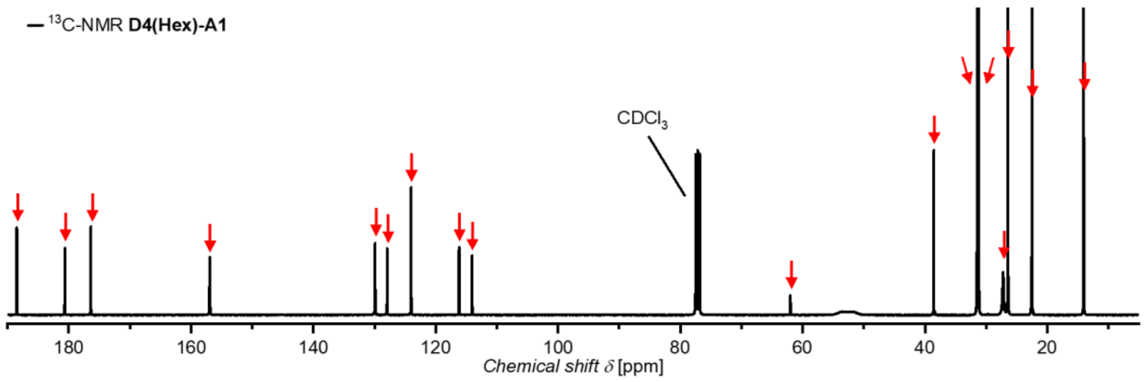

Figure S59. ^{13}C NMR of compound **D4(Hex)-A1** measured in $CDCl_3$ at 295 K and 101 MHz.

(*Z*)-2-(4-(*tert*-Butyl)-5-((2-(pyrrolidin-1-yl)thiazol-5-yl)methylene)thiazol-2(5*H*)-ylidene)malononitrile – D4(Pyrl)-A1:

A solution of **P-D4(Pyrl)** (2-(pyrrolidine-1-yl)thiazole-5-carbaldehyde, 224 mg, 1.23 mmol, 1.0 equiv.) and **P-A1** (2-[4-(*tert*-butyl)thiazol-2(3*H*)-ylidene]malononitrile, 252 mg, 1.23 mmol, 1.0 equiv.) in 3.0 mL Ac_2O was heated to 90 °C for 60 min. The reaction mixture was extracted using CH_2Cl_2 and H_2O, and the crude product further purified by silica-gel column chromatography using a gradient from CH_2Cl_2 to CH_2Cl_2:ethylacetate 99:1 and precipitation from CH_2Cl_2 and *n*-hexane. The precipitate was collected by filtration, washed with *n*-hexane, and dried under vacuum.

Yield: 140 mg (= 31 %) of a deep blue powder.

Melting point: ~ 290 °C. Mass spectrometry (MALDI-TOF): $[M]^+$ found at 369.10879. Calculated for $C_{18}H_{19}N_5S_2^+$ at 369.10764. ^1H NMR (400 MHz, $CDCl_3$, 295 K): δ / ppm = 8.02 (d, J = 0.5 Hz, 1H), 7.79 (d, J = 0.5 Hz, 1H), 3.67 (broad, 4H), 2.18 (m, 4H), 1.54 (s, 9H). ^{13}C NMR (101 MHz, $CDCl_3$, 295 K): δ / ppm = 188.8, 180.6, 173.1, 155.9, 129.3, 128.7, 124.4, 115.9, 113.7, 63.2, 38.6, 31.2, 25.6 (13 carbon signals in total). UV/Vis ($CHCl_3$): λ_{00} [nm] (ε [M^{-1} cm^{-1}]) = 606 (52 000).

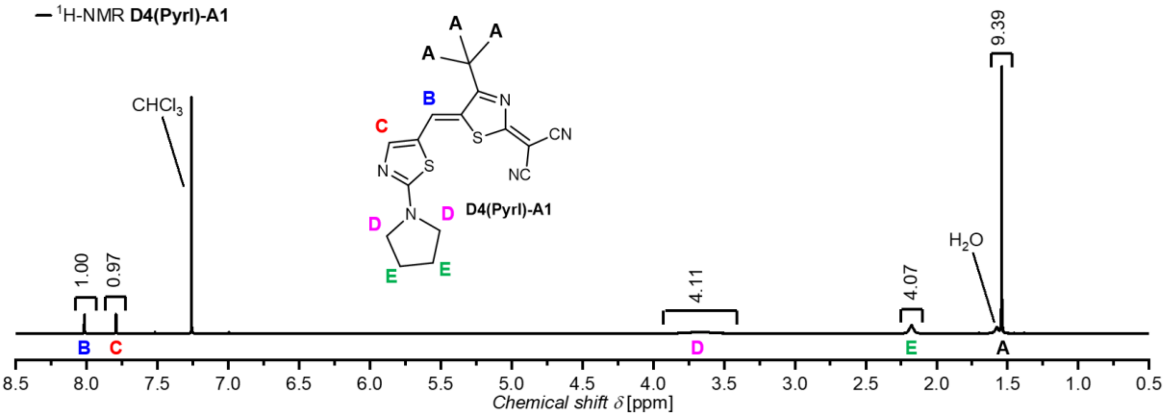

Figure S60. ^1H NMR of compound **D4(Pyrl)-A1** measured in $CDCl_3$ at 295 K and 400 MHz.

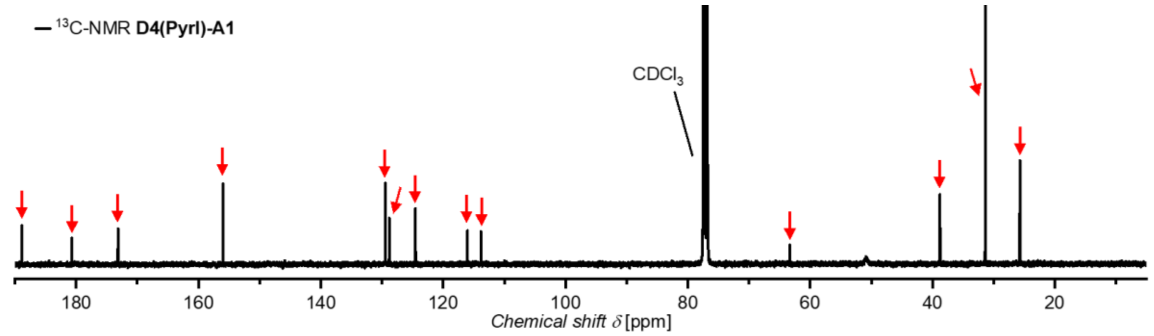

Figure S61. ^{13}C NMR of compound **D4(Pyrl)-A1** measured in $CDCl_3$ at 295 K and 101 MHz.

(*Z*)-2-(4-(*tert*-butyl)-5-((5-methoxythiophen-2-yl)methylene)thiazol-2(5*H*)-ylidene)malononitrile – D5(OMe)-A1:

A solution of **P-D5(OMe)** (5-methoxythiophene-2-carbaldehyde, 750 mg, 5.28 mmol, 1.0 equiv.) and **P-A1** (2-[4-(*tert*-butyl)thiazol-2(3*H*)-ylidene]malononitrile, 1 080 mg, 5.28 mmol, 1.0 equiv.) in 13 mL Ac_2O was heated to 115 °C for 16 h. After cooling to room temperature, 50 mL of diethyl ether were added and the resulting precipitate was collected by filtration and washed with isopropanol and diethyl ether. The product was purified by silica-gel column chromatography using CH_2Cl_2, precipitated from CH_2Cl_2 and *n*-hexane, collected by filtration, and dried under vacuum. The obtained solid was further purified by gradient sublimation.

Yield: 870 mg (= 50 %) of a dark blue solid.

Melting point: ~ 215 °C. Mass spectrometry (MALDI-TOF): $[M]^-$ found at 329.08542. Calculated for $C_{16}H_{15}N_3OS_2^-$ at 329.06620. ^{1}H NMR (400 MHz, $CDCl_3$, 295 K): δ / ppm = 7.98 (s, 1H), 7.36 (d, J = 4.4 Hz, 1H), 6.46 (d, J = 4.4 Hz, 1H), 4.10 (s, 3H), 1.55 (s, 9H). ^{13}C NMR (101 MHz, $CDCl_3$, 295 K): δ / ppm = 190.1, 181.2, 177.3, 138.7, 131.2, 129.7, 125.9, 115.2, 113.2, 108.9, 61.6, 38.9, 31.3 (13 carbon signals in total). UV/Vis ($CHCl_3$): λ_{00} [nm] (ε [M^{-1} cm^{-1}]) = 578 (40 000).

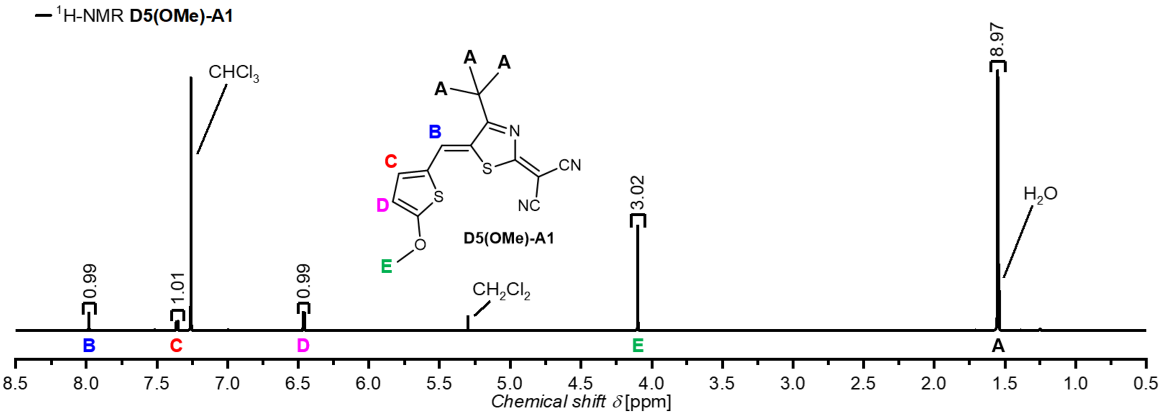

Figure S62. ^{1}H NMR of compound **D5(OMe)-A1** measured in $CDCl_3$ at 295 K and 400 MHz.

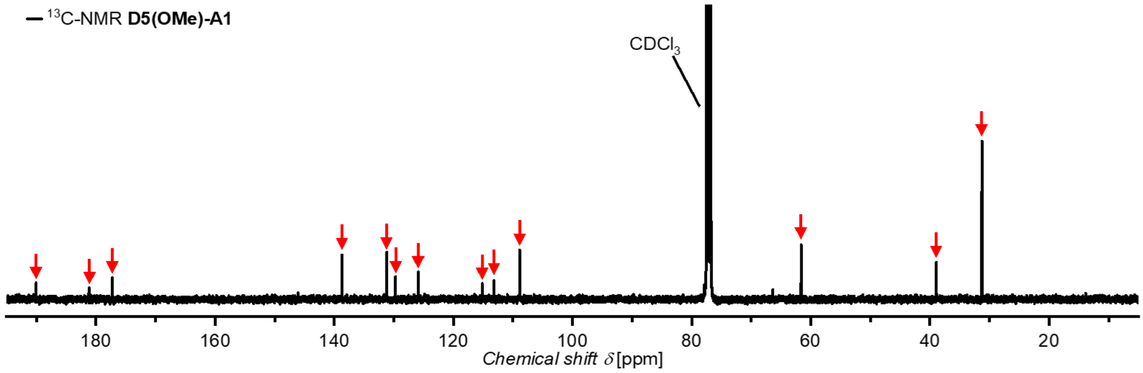

Figure S63. ^{13}C NMR of compound **D5(OMe)-A1** measured in $CDCl_3$ at 295 K and 101 MHz.

(*Z*)-2-(4-(*tert*-butyl)-5-((5-(tridecan-7-yl)thiophen-2-yl)methylene)thiazol-2(5*H*)-ylidene)malononitrile – D6(Hex)-A1:

A solution of **P-D6(Hex)** (5-(tridecan-7-yl)thiophene-2-carbaldehyde, 256 mg, 0.87 mmol, 1.0 equiv.) and **P-A1** (2-[4-(*tert*-butyl)thiazol-2(3*H*)-ylidene]malononitrile, 178 mg, 0.87 mmol, 1.0 equiv.) in 2.1 mL Ac_2O was heated to 100 °C for 22 h. The reaction mixture was extracted using CH_2Cl_2 and H_2O and the crude product purified by flash silica-gel column chromatography using a gradient from CH_2Cl_2:cyclohexane 1:3 to 1:1 and precipitation from CH_2Cl_2 and methanol. The precipitate was collected by filtration, washed with methanol, and dried under vacuum.

Yield: 23 mg (= 6 %) of a red solid.

Melting point: ~ 155 °C. Mass spectrometry (MALDI-TOF): $[M]^-$ found at 481.25855. Calculated for $C_{28}H_{39}N_3S_2^-$ at 481.25909. ^1H NMR (400 MHz, $CDCl_3$, 295 K): δ / ppm = 8.06 (s, 1H), 7.41 (d, J = 3.9 Hz, 1H), 6.94 (d, J = 3.9 Hz, 1H), 2.92 (m, 1H), 1.58–1.79 (m, 4H), 1.56 (s, 9H), 1.16–1.34 (m, 16H), 0.86 (t, J = 7.0 Hz, 6H). ^{13}C NMR (101 MHz, $CDCl_3$, 295 K): δ / ppm = 190.7, 181.4, 164.7, 137.5, 136.5, 132.6, 130.1, 126.9, 114.7, 112.8, 43.0, 39.0, 38.0, 31.8, 31.2, 29.3, 27.6, 22.7, 14.2 (19 carbon signals in total). UV/Vis ($CHCl_3$): λ_{00} [nm] (ε [M^{-1} cm^{-1}]) = 554 (34 000).

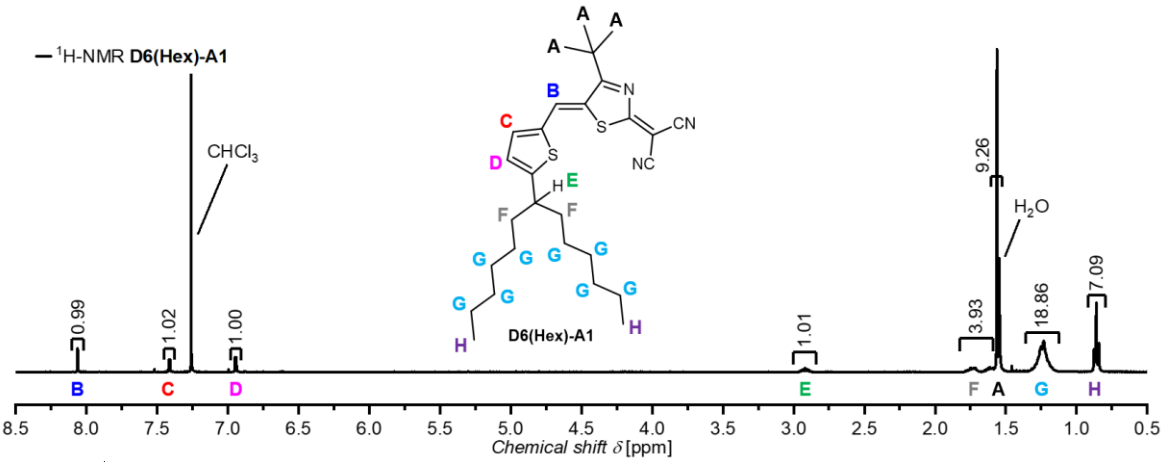

Figure S64. ^1H NMR of compound **D6(Hex)-A1** measured in $CDCl_3$ at 295 K and 400 MHz.

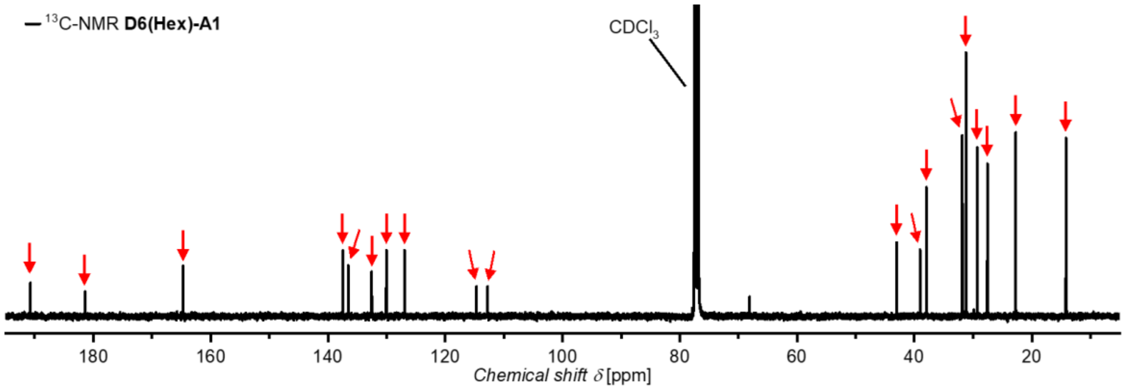

Figure S65. ^{13}C NMR of compound **D6(Hex)-A1** measured in $CDCl_3$ at 295 K and 101 MHz.

(*Z*)-2-(4-(*tert*-butyl)-5-((5-cyclopentylthiophen-2-yl)methylene)thiazol-2(5*H*)-ylidene)malononitrile – D6(cPen)-A1:

A solution of **P-D6(cPen)** (5-cyclopentylthiophene-2-carbaldehyde, 200 mg, 1.11 mmol, 1.0 equiv.) and **P-A1** (2-[4-(*tert*-butyl)thiazol-2(3*H*)-ylidene]malononitrile, 228 mg, 1.11 mmol, 1.0 equiv.) in 3 mL Ac_2O was heated to 90 °C for 20 h. The reaction mixture was extracted with CH_2Cl_2 and H_2O and the crude product further purified by silica-gel column chromatography using CH_2Cl_2:*n*-hexane 99:1 and precipitation from CH_2Cl_2 and *n*-hexane. The precipitate was collected by filtration, washed with *n*-hexane, and dried under vacuum.

Yield: 285 mg (= 70 %) of a dark purple solid.

Melting point: ~ 215 °C. Mass spectrometry (MALDI-TOF): $[M]^-$ found at 367.16104. Calculated for $C_{20}H_{21}N_3S_2^-$ at 367.11824. ^1H NMR (400 MHz, $CDCl_3$, 295 K): δ / ppm = 8.05 (s, 1H), 7.40 (d, J = 4.0 Hz, 2H), 6.99 (dd, J = 4.0 Hz, J = 0.9 Hz, 2H), 3.36 (quin, J = 8.1 Hz, 1H), 2.17–2.26 (m, 2H), 1.81–1.92 (m, 2H), 1.66–1.80 (m, 4H), 1.56 (s, 9H). ^{13}C NMR (101 MHz, $CDCl_3$, 295 K): δ / ppm = 190.7, 181.4, 164.6, 137.7, 136.3, 132.5, 130.1, 126.1, 114.7, 112.8, 42.3, 39.0, 35.7, 31.2, 25.5 (15 carbon signals in total). UV/Vis ($CHCl_3$): λ_{00} [nm] (ε [M^{-1} cm^{-1}]) = 554 (36 000).

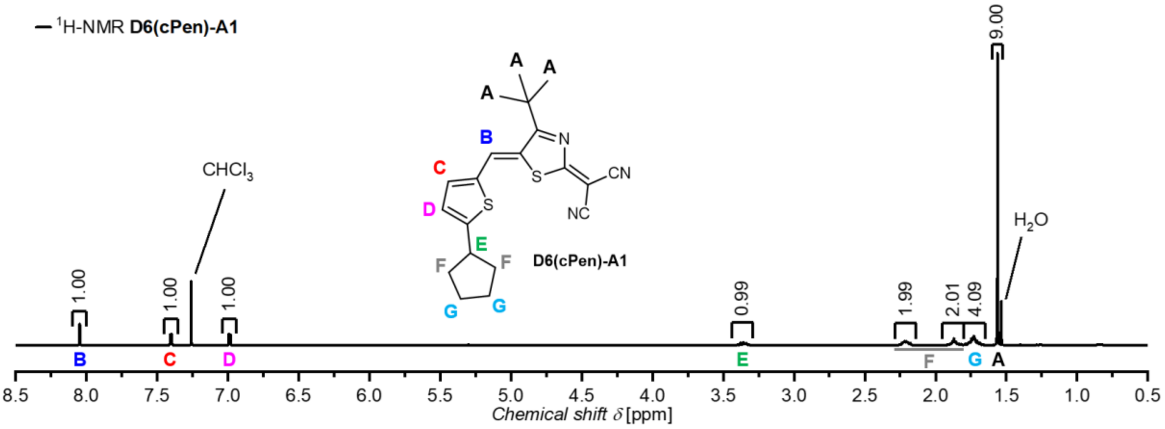

Figure S66. ^1H NMR of compound **D6(cPen)-A1** measured in $CDCl_3$ at 295 K and 400 MHz.

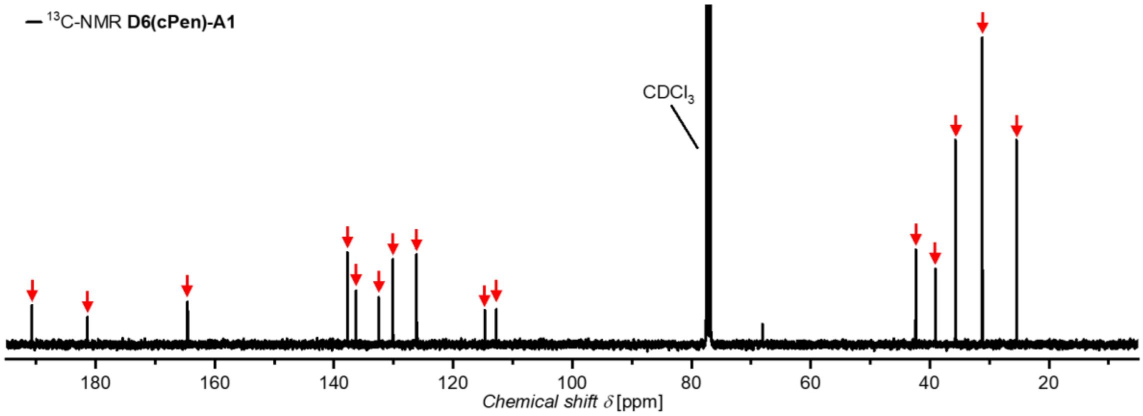

Figure S67. ^{13}C NMR of compound **D6(cPen)-A1** measured in $CDCl_3$ at 295 K and 101 MHz. The peaks below 70 ppm were assigned using additional ^1H ^{13}C HSQC and HMBC NMR measurements.

(*Z*)-2-(5-((5-(dibutylamino)selenophen-2-yl)methylene)-4-methylthiazol-2(5*H*)-ylidene)malononitrile – D1(Bu)-A2:

A solution of **P-D1(Bu)** (2-(dibutylamino)selenophene-5-carbaldehyde, 219 mg, 0.76 mmol, 1 equiv.) and **P-A2** (2-(4-methylthiazol-2(3*H*)-ylidene)malononitrile, 125 mg, 0.76 mmol, 1 equiv.) in 1.9 mL Ac_2O was heated to 90 °C for 60 min. The reaction mixture was extracted using CH_2Cl_2 and H_2O, the organic phase was dried under vacuum, and the residuum precipitated from CH_2Cl_2 and *n*-hexane. The precipitate was collected by filtration, washed with *n*-hexane, and dried under vacuum.

Yield: 248 mg (= 75 %) of a deep blue powder.

Melting point: ~ 160 °C. Mass spectrometry (MALDI-TOF): $[M]^-$ found at 432.14075. Calculated for $C_{20}H_{24}N_4SSe^-$ at 432.08924. ^1H NMR (400 MHz, $CDCl_3$, 295 K): δ / ppm = 7.71 (s, 1H), 7.61 (d, J = 5.2 Hz, 1H), 6.25 (d, J = 5.2 Hz, 1H), 3.50 (t, J = 7.8 Hz, 4H), 2.56 (s, 3H), 1.72–1.80 (m, 4H), 1.44 (m, 4H), 1.02 (t, J = 7.4 Hz, 6H). ^{13}C NMR (101 MHz, $CDCl_3$, 295 K): δ / ppm = 180.9, 179.5, 174.0, 150.3, 134.2, 128.1, 124,0, 118.5, 116.5, 112.0, 57.0, 29.6, 20.5, 17.1, 13.9 (15 carbon signals in total). UV/Vis ($CHCl_3$): λ_{00} [nm] (ε [M^{-1} cm^{-1}]) = 655 (119 000).

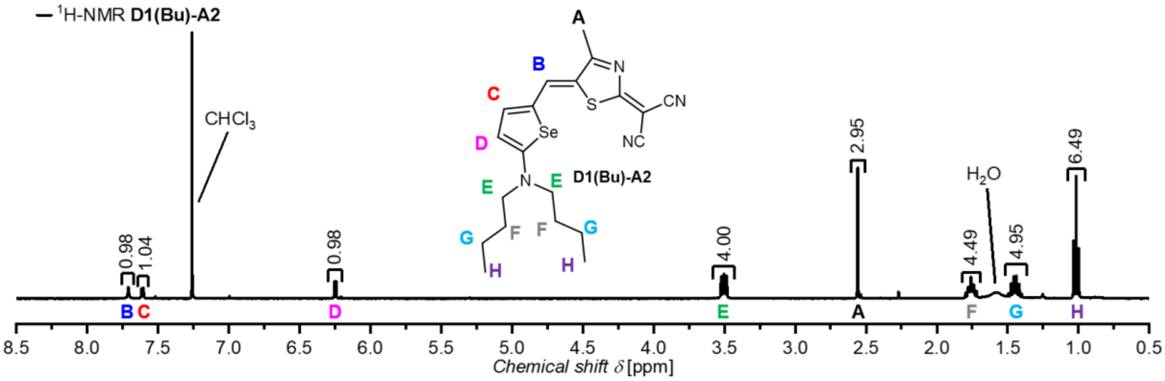

Figure S68. ^1H NMR of compound **D1(Bu)-A2** measured in $CDCl_3$ at 295 K and 400 MHz.

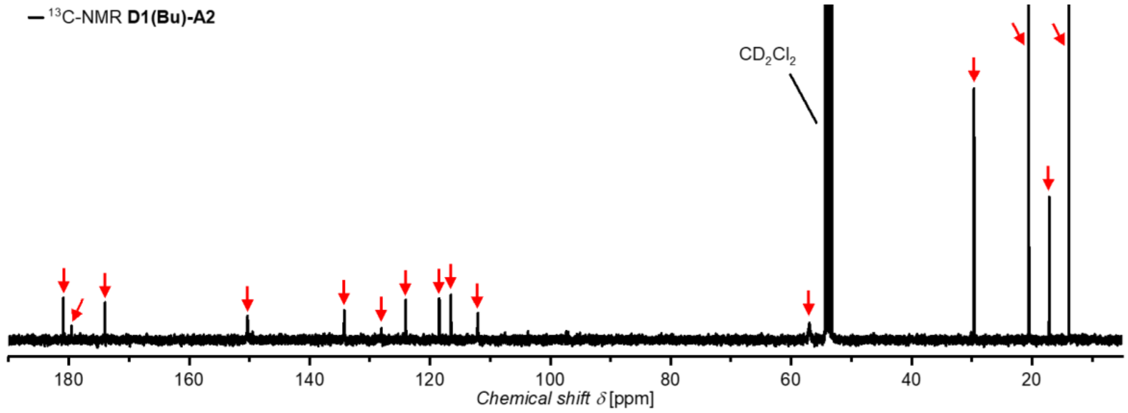

Figure S69. ^{13}C NMR of compound **D1(Bu)-A2** measured in CD_2Cl_2 at 295 K and 101 MHz.

(Z)-2-(5-((5-(Dibutylamino)thiophen-2-yl)methylene)-4-methylthiazol-2(5*H*)-ylidene)malononitrile – D3(Bu)-A2:

D3(Bu)-A2 was synthesized according to literature.[S4] A solution of **P-D3(Bu)** (5-(dibutylamino)thiophene-2-carbaldehyde, 622 mg, 2.60 mmol, 1.0 equiv.) and **P-A2** (2-(4-methylthiazol-2(3*H*)-ylidene)malononitrile, 424 mg, 2.60 mmol, 1.0 equiv.) in 2.8 mL Ac_2O was heated to 90 °C for 45 min. The reaction mixture was precipitated from diethyl ether, collected by filtration, and washed with diethyl ether, isopropanol, and *n*-hexane. The product was further purified by silica-gel column chromatography using CH_2Cl_2:methanol 99.5:0.5, suspended in *n*-hexane, collected by filtration, and dried under vacuum.

Yield: 559 mg (= 56 %) of a dark green powder.

Melting point: ~ 165 °C. Mass spectrometry (MALDI-TOF): $[M]^+$ found at 384.14369. Calculated for $C_{20}H_{24}N_4S_2^+$ at 384.14369. ^{1}H NMR (400 MHz, $CDCl_3$, 295 K): δ / ppm = 7.61 (s, 1H), 7.43 (d, J = 5.2 Hz, 1H), 6.23 (d, J = 5.2 Hz, 1H), 3.49 (t, J = 7.7 Hz, 4H), 2.54 (s, 3H), 1.67–1.75 (m, 4H), 1.42 (sext, J = 7.4 Hz, 4H), 0.99 (t, J = 7.4 Hz, 6H). ^{13}C NMR (101 MHz, $CDCl_3$, 295 K): δ / ppm = 181.6, 175.1, 172.2, 145.8, 130.9, 124.5, 124.0, 118.0, 115.8, 109.4, 54,7, 29.5, 20.3, 17.1, 14.0 (15 carbon signals in total). UV/Vis ($CHCl_3$): λ_{00} [nm] (ε [M^{-1} cm^{-1}]) = 648 (137 000).

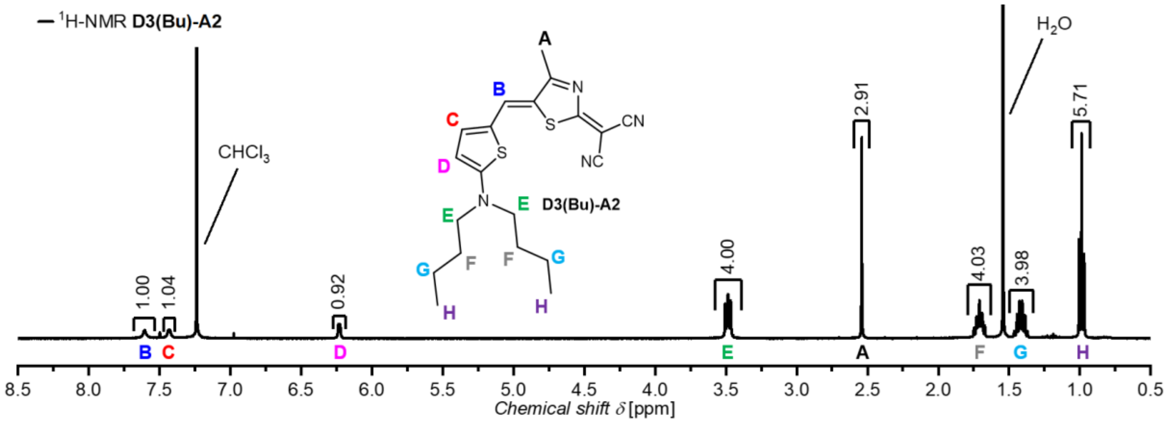

Figure S70. ^{1}H NMR of compound **D3(Bu)-A2** measured in $CDCl_3$ at 295 K and 400 MHz.

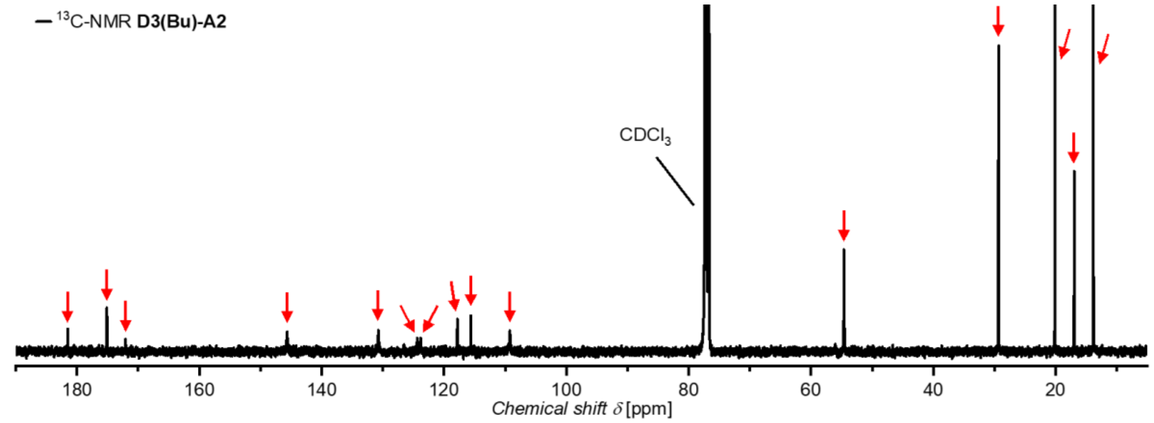

Figure S71. ^{13}C NMR of compound **D3(Bu)-A2** measured in $CDCl_3$ at 295 K and 101 MHz.

(*Z*)-2-(5-((2-(Dibutylamino)thiazol-5-yl)methylene)-4-methylthiazol-2(5*H*)-ylidene)malononitrile – D4(Bu)-A2:

A solution of **P-D4(Bu)** (2-(dibutylamino)thiazole-5-carbaldehyde, 288 mg, 1.20 mmol, 1.0 equiv.) and **P-A2** (2-(4-methylthiazol-2(3*H*)-ylidene)malononitrile, 196 mg, 1.20 mmol, 1.0 equiv.) in 3.0 mL Ac_2O was heated to 90 °C for 60 min and, after the addition of 20 mL H_2O, left to stir for 60 min at room temperature. The precipitate was collected by filtration, washed with ethanol, isopropanol, and *n*-hexane, and dried under vacuum. The product was purified by flash silica-gel column chromatography using a gradient from CH_2Cl_2 to CH_2Cl_2:ethyl acetate 92.5:7.5, precipitated from CH_2Cl_2 and *n*-hexane, collected by filtration, washed with *n*-hexane, and dried under vacuum.

Yield: 213 mg (= 46 %) of a dark green powder.

Melting point: ~ 190 °C. Mass spectrometry (MALDI-TOF): $[M]^-$ found at 385.13968. Calculated for $C_{19}H_{23}N_5S_2^-$ at 385.14004. ^{1}H NMR (400 MHz, $CDCl_3$, 295 K): δ / ppm = 7.79 (s, 1H), 6.67 (s, 1H), 3.57 (broad, 4H), 2.61 (s, 3H), 1.69 (m, 4H), 1.40 (m, 4H), 0.98 (t, J = 7.3 Hz, 6H). ^{13}C NMR (101 MHz, $CDCl_3$, 295 K): δ / ppm = 182.2, 179.1, 176.5, 156.5, 129.6, 128.5, 124.3, 116.0, 113.9, 62.5, 29.5, 20.2, 17.3, 14.0 (14 carbon signals in total). UV/Vis ($CHCl_3$): λ_{00} [nm] (ε [M^{-1} cm^{-1}]) = 610 (54 000).

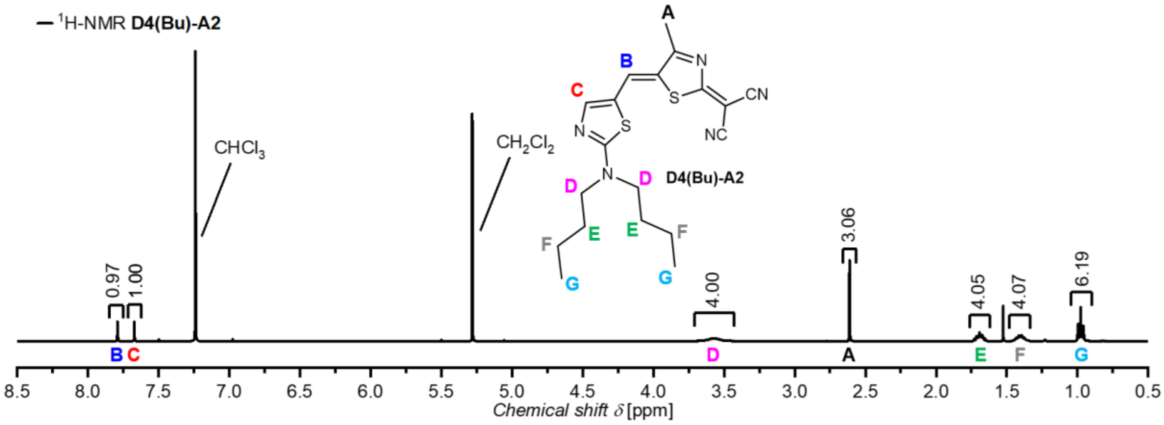

Figure S72. ^{1}H NMR of compound **D4(Bu)-A2** measured in $CDCl_3$ at 295 K and 400 MHz.

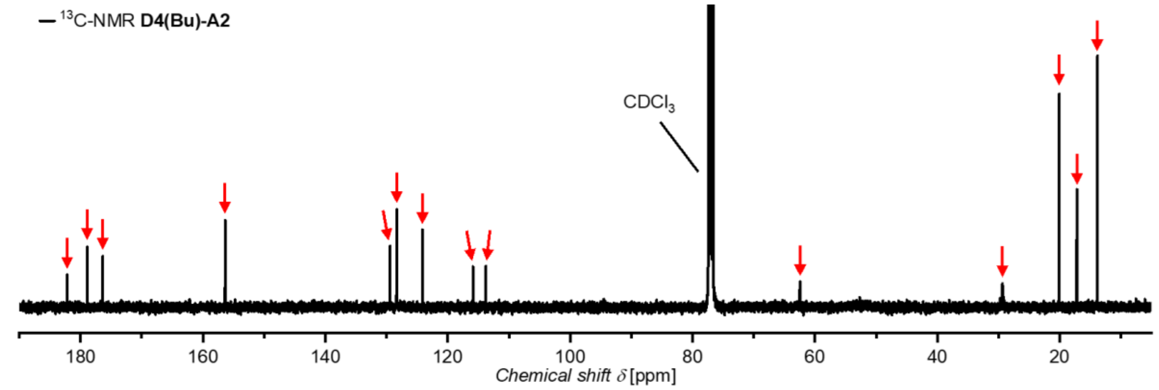

Figure S73. ^{13}C NMR of compound **D4(Bu)-A2** measured in $CDCl_3$ at 295 K and 101 MHz.

15 Supporting References

[S1] Liess, A.; Lv, A.; Arjona-Esteban, A.; Bialas, D.; Krause, A.-M.; Stepanenko, V.; Stolte, M.; Würthner, F. Exciton Coupling of Merocyanine Dyes from H- to J-type in the Solid State by Crystal Engineering. *Nano Lett.* **2017**, *17*, 1719–1726.

[S2] Bürckstümmer, H.; Tulyakova, E. V.; Deppisch, M.; Lenze, M. R.; Kronenberg, N. M.; Gsänger, M.; Stolte, M.; Meerholz, K.; Würthner, F. Efficient Solution-Processed Bulk Heterojunction Solar Cells by Antiparallel Supramolecular Arrangement of Dipolar Donor–Acceptor Dyes. *Angew. Chem. Int. Ed.* **2011**, *50*, 11628–11632; *Angew. Chem.* **2011**, *123*, 11832–11836.

[S3] Liess, A.; Arjona-Esteban, A.; Kudzus, A.; Albert, J.; Krause, A.-M.; Lv, A.; Stolte, M.; Meerholz, K.; Würthner, F. Ultranarrow Bandwidth Organic Photodiodes by Exchange Narrowing in Merocyanine H- and J-Aggregate Excitonic Systems. *Adv. Funct. Mater.* **2019**, *29*, 1805058.

[S4] Würthner, F.; Thalacker, C.; Matschiner, R.; Lukaszuk, K.; Wortmann, R. Optimization of neutrocyanine chromophores based on five-membered heterocycles for photorefractive applications. *Chem. Commun.* **1998**, 1739–1740.

[S5] Antonov, D. N.; Belen'Kii, L. I.; Gronowitz, S. On the selectivity in the bromination of selenophene-2-carbonyl derivatives in the presence of aluminum trichloride. *J. Heterocyclic Chem.* **1995**, *32*, 53–55.

[S6] Shoji, H.; Kobatake, S. Thermal bleaching reactions of photochromic diarylethenes with thiophene-*S*,*S*-dioxide for a light-starting irreversible thermosensor. *Chem. Commun.* **2013**, *49*, 2362–2364.

[S7] Prim, D.; Kirsch, G.; Nicoud, J.-F. Efficient synthesis of *N*,*N*-disubstituted 5-aminothiophene-2-carboxaldehydes by nucleophilic aromatic substitution in water. *Synlett* **1998**, *4*, 383–384.

[S8] Aldalbahi, A.; Periyasami, G.; Alrehaili, A. Synthesis of high molar extinction coefficient push–pull tricyanofuran-based disperse dyes: Biological activity and dyeing performance. *New. J. Chem.* **2021**, *45*, 2208–2216.

[S9] Meier, H.; Petermann, R. NIR Absorbing Squaraines by Extension of the Conjugation with (Aminothiazolyl)ethenyl Groups. *Helv. Chim. Acta* **2004**, *87*, 1109–1118.

[S10] Sicé, J. Preparation and Reactions of 2-Methoxythiophene. *J. Am. Chem. Soc.* **1953**, *75*(15), 3697–3700.

[S11] Al-Mousawi, S. M.; Moustafa, M. S.; Elnagdi, M. H. Reassignment of the Structures of Products Produced by Reactions of the Product Believed To Be 2-(1-Phenyl-2-Thiocyanatoethylidene)-malononitrile with Electrophiles. *Molecules* **2011**, *16*, 3456–3468.

[S12] Fulmer, G. R.; Miller, A. J. M.; Sherden, N. H.; Gottlieb, H. E.; Nudelman, A.; Stoltz, B. M.; Bercaw, J. E.; Goldberg, K. I. NMR Chemical Shifts of Trace Impurities: Common Laboratory Solvents, Organics, and Gases in Deuterated Solvents Relevant to the Organometallic Chemist. *Organometallics* **2010**, *29*, 2176–2179.

[S13] Kuball, H.-G.; Stolte, M. Electro-Optical Absorption Spectroscopy. In *Comprehensive Chiroptical Spectroscopy: Instrumentation, Methodologies, and Theoretical Simulations, Vol. 1;* Nina Berova, N.; Polavarapu, P. L.; Nakanishi, K.; Woody, R. W., Eds.; John Wiley & Sons, Inc, **2012**; pp 525-540.

[S14] Baumann, W. *Physical Methods of Chemistry, Vol. 3B*; Rossiter, B. W.; Hamilton, J. F.; Eds.; Wiley, New York, **1989**; p 45.

[S15] Würthner, F.; Yao, S.; Schilling, J.; Wortmann, R.; Redi-Abshiro, M.; Mecher, E.; Gallego-Gomez, F.; Meerholz, K. ATOP Dyes. Optimization of a Multifunctional Merocyanine Chromophore for High Refractive Index Modulation in Photorefractive Materials. *J. Am. Chem. Soc.* **2001**, *123*, 2810–2824.

[S16] Cardona, C. M.; Li, W.; Kaifer, A. E.; Stockdale, D.; Bazan, G. C. Electrochemical Considerations for Determining Absolute Frontier Orbital Energy Levels of Conjugated Polymers for Solar Cell Applications. *Adv. Mater.* **2011**, *23*, 2367–2371.

[S17] Connelly, N. G.; Geiger, W. E. Chemical Redox Agents for Organometallic Chemistry. *Chem. Rev.* **1996**, *96*, 877–910.

[S18] Hansen, W. N.; Hansen, G. J. Absolute half-cell potential: A simple direct measurement. *Phys. Rev. A* **1987**, *36*(3), 1396–1402.

[S19] Kabsch, W. XDS. *Acta Crystallogr. D* **2010**, *66*, 125–132.

[S20] Sheldrick, G. M. SHELXT – Integrated space-group and crystal-structure determination. *Acta Crystallogr. A* **2015**, *71*, 3–8.

[S21] Sheldrick, G. M. Crystal structure refinement with SHELXL. *Acta Crystallogr. C* **2015**, *71*, 3–8.

[S22] Frisch, M. J.; Trucks, G. W.; Schlegel, H. B.; G. E. Scuseria, G. E.; Robb, M. A.; J. R. Cheeseman, J. R.; Scalmani, G.; Barone, V.; Mennucci, B.; Petersson, G. A.; Nakatsuji, H.; Caricato, M.; Li, X.; Hratchian, H. P.; Izmaylov, A. F.; Bloino, J.; Zheng, G.; Sonnenberg, J. L.; Hada, M. ; Ehara, M.; Toyota, K.; Fukuda, R.; Hasegawa, J.; Ishida, M.; Nakajima, T.; Honda, Y. ; Kitao, O.; Nakai, H.; Vreven, T.; Montgomery Jr., J. A.; Peralta, J. E.; Ogliaro, F.; Bearpark, M.; Heyd, J. J.; Brothers, E.; Kudin, K. N.; Staroverov, V. N.; Keith, T.; Kobayashi, R.; Normand, J.; Raghavachari, K.; Rendell, A.; Burant, J. C.; Iyengar, S. S.; Tomasi, J.; Cossi, M.; Rega, N.; Millam, J. M.; Klene, M.; Knox, J. E.; Cross, J. B.; Bakken, V.; Adamo, C.; Jaramillo, J.; Gomperts, R.; Stratmann, R. E.; Yazyev, O.; Austin, A. J.; Cammi, R.; Pomelli, C.; Ochterski, J. W.; Martin, R. L.; Morokuma, K.; Zakrzewski, V. G.; Voth, G. A.; Salvador, P.; Dannenberg, J. J.; Dapprich, S.; Daniels, A. D.; Farkas, O.; Foresman, J. B.; Ortiz, J. V.; Cioslowski, J.; Fox, D. J. Gaussian 09, Revision D.01. Gaussian, Inc., Wallingford CT, 2013.

[S23] Yanai, T.; Tew, D. P.; Handy, N. C. A new hybrid exchange-correlation functional using the Coulomb-attenuating method (CAM-B3LYP). *Chem. Phys. Lett.* **2004**, *393*, 51–57.

[S24] Rassolov, V. A.; Ratner, M. A.; Pople, J. A.; Redfern, P. C.; Curtiss, L. A. 6-31G* basis set for third-row atoms. *J. Comput. Chem.* **2001**, *22*(9), 976–984.

[S25] a) Singh, U. C.; Kollman, P. A. An approach to computing electrostatic charges for molecules. *J. Comput. Chem.* **1984**, *5*(2), 129; b) Besler, B. H.; Merz Jr, K. M.; Kollman, P. A. Atomic charges derived from semiempirical methods. *J. Comput. Chem.* **1990**, *11*(4), 431.

[S26] Scholes, G. D.; Ghiggino, K. P. Electronic interactions and interchromophore excitation transfer. *J. Phys. Chem.* **1994**, *98*, 4580–4590.

[S27] Hestand, N. J.; Spano, F. C. Molecular Aggregate Photophysics beyond the Kasha Model: Novel Design Principles for Organic Materials. *Acc. Chem. Res.* **2017**, *50*, 341.

[S28] Te Velde, G.; Bickelhaupt, F. M.; Baerends, E. J.; Fonseca Guerra, C.; van Gisbergen, S. J. A.; Snijders, J. G.; Ziegler, T. Chemistry with ADF. *J. Comput. Chem.* **2001**, *22*, 931–967.

[S29] ADF 2013, SCM, Theoretical Chemistry, Vrije Universiteit, Amsterdam, The Netherlands, http://www.scm.com.

[S30] Perdew, J. P.; Burke, K.; Wang, Y. Generalized gradient approximation for the exchange-correlation hole of a many-electron system. *Phys. Rev. B* **1996**, *54*, 16533–16539.

[S31] Barbieri, P. L.; Fantin, P. A.; Jorge, F. E. Gaussian basis sets of triple and quadruple zeta valence quality for correlated wave functions. *Mol. Phys.* **2006**, *104*, 2945–2954.

[S32] Senthilkumar, K.; Crozema, F. C.; Bickelhaupt, F. M.; Siebbeles, L. D. A. Charge transport in columnar stacked triphenylenes: Effects of conformational fluctuations on charge transfer integrals and site energies. *J. Chem. Phys.* **2003**, *119*, 9809–9817.

[S33] Wen, S.-H.; Li, A.; Song, J.; Deng, W.-Q.; Han, L.-K.; Goddard, A. First-Principles Investigation of Anistropic Hole Mobilities in Organic Semiconductors. *J. Phys. Chem. B* **2009**, *113*, 8813–8819.

[S34] Becke, A. D. A new mixing of Hartree–Fock and local density-functional theories. *J. Chem. Phys.* **1993**, *98*(7), 5648–5652.

[S35] Lee, C.; Yang, W.; Parr, R. G. Development of the Colle-Salvetti correlation-energy formula into a functional of the electron density. *Phys. Rev. B* **1988**, *37*(2), 785–789.

[S36] Weigend, F.; Ahlrichs, R. Balanced basis sets of split valence, triple zeta valence and quadruple zeta valence quality for H to Rn: Design and assessment of accuracy. *Phys. Chem. Chem. Phys.* **2005**, *7*, 3297–3305.

[S37] Schwarze, M.; Tress, W.; Beyer, B.; Gao, F.; Scholz, R.; Poelking, C.; Ortstein, K.; Günther, A. A.; Kasemann, D.; Andrienko, D.; Leo, K. Band structure engineering in organic semiconductors. *Science* **2016**, *352*, 1446–1449.

[S38] Spackman, P. R.; Turner, M. J.; McKinnon, J. J.; Wolff, S. K.; Grimwood, D. J.; Jayatilaka, D.; Spackman, M. A. CrystalExplorer: a program for Hirshfeld surface analysis, visualization and qu-antitative analysis of molecular crystals. *J. Appl. Cryst.* **2021**, *54*, 1006–1011.

[S39] Mackenzie, C. F.; Spackman, P. R.; Jayatilaka, D.; Spackman, M. A. *CrystalExplorer* model energies and energy frameworks: extension to metal coordination compounds, organic salts, solvates and open-shell systems. *IUCrJ* **2017**, *4*, 575–587.

[S40] Schembri, T.; Kolb, L.; Stolte, M.; Würthner, F. Polarized, color-selective and semi-transparent organic photodiode of aligned merocyanine H-aggregates. *J. Mater. Chem. C* **2024**, *12*, 4948–4953.

[S41] Yoon, S.; Sim, K. M.; Chung, D. S. Prospects of colour selective organic photodiodes. *J. Mater. Chem. C* **2018**, *6*, 13084.

[S42] Reichardt, C.; Welton, T. *Solvents and Solvent Effects in Organic Chemistry, 4th Ed. App. A*; Wiley-VCH Verlag GmbH & Co. KGaA, 2010.

[S43] Kim, J. H.; Schembri, T.; Bialas, D.; Stolte, M.; Würthner, F. Slip-Stacked J-Aggregate Materials for Organic Solar Cells and Photodetectors. *Adv. Mater.* **2022**, *34*, 2104678.

[S44] Giavazzi, D.; Schumacher, M. F.; Grisanti, L.; Anzola, M.; Di Maiolo, F.; Zablocki, J.; Lützen, A.; Schiek, M.; Painelli, A. A marvel of chiral squaraine aggregates: chiroptical spectra beyond the exciton model. *J. Mater. Chem. C* **2023**, *11*, 8307–8321.

[S45] Kirchner, E.; Bialas, D.; Fennel, F.; Grüne, M.; Würthner, F. Defined Merocyanine Dye Stacks from a Dimer up to an Octamer by Spacer-Encoded Self-Assembly Approach. *J. Am. Chem. Soc.* **2019**, *141*, 7428–7438.

[S46] Herbert, B.; Walpuski, J.; Stolte, M.; Shoyama, K. Designing Organic π-Conjugated Molecules for Crystalline Solid Solutions: Adamantane-Substituted Naphthalenes. *ChemPlusChem* **2024**, e202300761.

[S47] He, T.; Stolte, M.; Burschka, C.; Hansen, N. H.; Musiol, T.; Kälblein, D.; Pflaum, J.; Tao, X.; Brill, J.; Würthner, F. Single-crystal field-effect transistors of new Cl_2-NDI polymorph processed by sublimation in air. *Nat. Commun.* **2015**, *6*, 5954.

[S48] Bridgman, P. W. Certain Physical Properties of Single Crystals of Tungsten, Antimony, Bismuth, Tellurium, Cadmium, Zinc, and Tin. *Proc. Am. Acad. Arts Sci.* **1925**, *60*(6), 305–383.

[S49] Lipsett, F. R. ON THE PRODUCTION OF SINGLE CRYSTALS OF NAPHTHALENE AND ANTHRACENE. *Can. J. Phys.* **1957**, *35*, 284–298.

[S50] Günther, C.; Karl, N.; Pflaum, J.; Strohmaier, R.; Gompf, B.; Eisenmenger, W.; Müller, M.; Müllen, K. LEED, STM, and TDS Studies of Ordered Thin Films of the Rhombus-Shaped Polycondensed Aromatic Hydrocarbon $C_{54}H_{22}$, on MoS_2, GeS, and Graphite. *Langmuir* **2005**, *21*(2), 656–665.

[S51] Qiu, Y.; Gao, H.; Cao, S.; Zhang, Y.; Wie, Y.; Wie, X.; Li, X.; Zhang, X.; Jiang, L.; Zhao, Z.; Wu, Y. Interface-Confined Assembly of Layered Molecular Crystal Arrays Toward Wearable UV-Radiation Monitor. *Adv. Funct. Mater.* **2024**, 2313990.

[S52] He, Q.; Basu, A.; Cha, H.; Daboczi, M.; Panidi, J.; Tan, L.; Hu, X.; Huang, C. C.; Ding, B.; White, A. J. P.; Kim, J.-S.; Durrant, J. R.; Anthopoulos, T. D.; Heeney, M. Ultra-Narrowband Near-Infrared Responsive J-Aggregates of Fused Quinoidal Tetracyanoindacenodithiophene. *Adv. Mater.* **2023**, *35*, 2209800.

[S53] Shoji, H.; Kobatake, S. Thermal bleaching reactions of photochromic diarylethenes with thiophene-*S,S*-dioxide for a light-starting irreversible thermosensor. *Chem. Commun.* **2013**, *49*, 2362.

[S54] Aldalbahi, A.; Periyasami, G.; Alrehaili, A. Synthesis of high molar extinction coefficient push–pull tricyanofuran-based disperse dyes: Biological activity and dyeing performance. *New. J. Chem.* **2021**, *45*, 2208–2216.

[S55] Meier, H.; Petermann, R. NIR Absorbing Squaraines by Extension of the Conjugation with (Aminothiazolyl)ethenyl Groups. *Helv. Chim. Acta* **2004**, *87*, 1109–1118.
